# Designed allosteric protein logic

**DOI:** 10.1038/s41421-023-00635-y

**Published:** 2024-01-16

**Authors:** Tjaša Plaper, Estera Merljak, Tina Fink, Tadej Satler, Ajasja Ljubetič, Duško Lainšček, Vid Jazbec, Mojca Benčina, Sintija Stevanoska, Sašo Džeroski, Roman Jerala

**Affiliations:** 1 Department of Synthetic Biology and Immunology, National Institute of Chemistry, Hajdrihova 19, SI-1000 Ljubljana, Slovenia; 2 Interdisciplinary doctoral study of biomedicine, Medical Faculty, University of Ljubljana, 1000 Ljubljana, Slovenia; 3 Centre for Technologies of Gene and Cell Therapy, Hajdrihova 19, SI-1000 Ljubljana, Slovenia; 4 Department of knowledge technologies, Jožef Stefan Institute, Jamova cesta 39, 1000 Ljubljana, Slovenia

**Keywords:** Protein folding, Cell signalling

## Abstract

The regulation of protein function by external or internal signals is one of the key features of living organisms. The ability to directly control the function of a selected protein would represent a valuable tool for regulating biological processes. Here, we present a generally applicable regulation of proteins called INSRTR, based on inserting a peptide into a loop of a target protein that retains its function. We demonstrate the versatility and robustness of coiled-coil-mediated regulation, which enables designs for either inactivation or activation of selected protein functions, and implementation of two-input logic functions with rapid response in mammalian cells. The selection of insertion positions in tested proteins was facilitated by using a predictive machine learning model. We showcase the robustness of the INSRTR strategy on proteins with diverse folds and biological functions, including enzymes, signaling mediators, DNA binders, transcriptional regulators, reporters, and antibody domains implemented as chimeric antigen receptors in T cells. Our findings highlight the potential of INSRTR as a powerful tool for precise control of protein function, advancing our understanding of biological processes and developing biotechnological and therapeutic interventions.

## Introduction

Regulation of protein function is a critical aspect of complex biological systems and involves various mechanisms, such as transcriptional regulation, posttranslational modification, multi-molecular complex formation, and allostery. While transcriptional regulation has been widely used in engineered systems due to the ease of construction of regulatory modules^1–5^, direct regulation of proteins has been more challenging to engineer due to the unique characteristics of different protein folds. Recently, several strategies have been introduced for direct regulation of protein function through allostery^6–9^, degradation based on degrons (CHOMP)^10^, split protease and coiled-coil (CC)-mediated reconstitution of split proteins (SPOC), allosteric regulation through the introduction of FKBP^11^, and the displacement of the bioactive peptide from the locked conformation (LOCKR)^12,13^. However, these systems often require extensive optimization or screening, and their application has been demonstrated on a small number of specific protein types and functions.

**Fig. 1:**
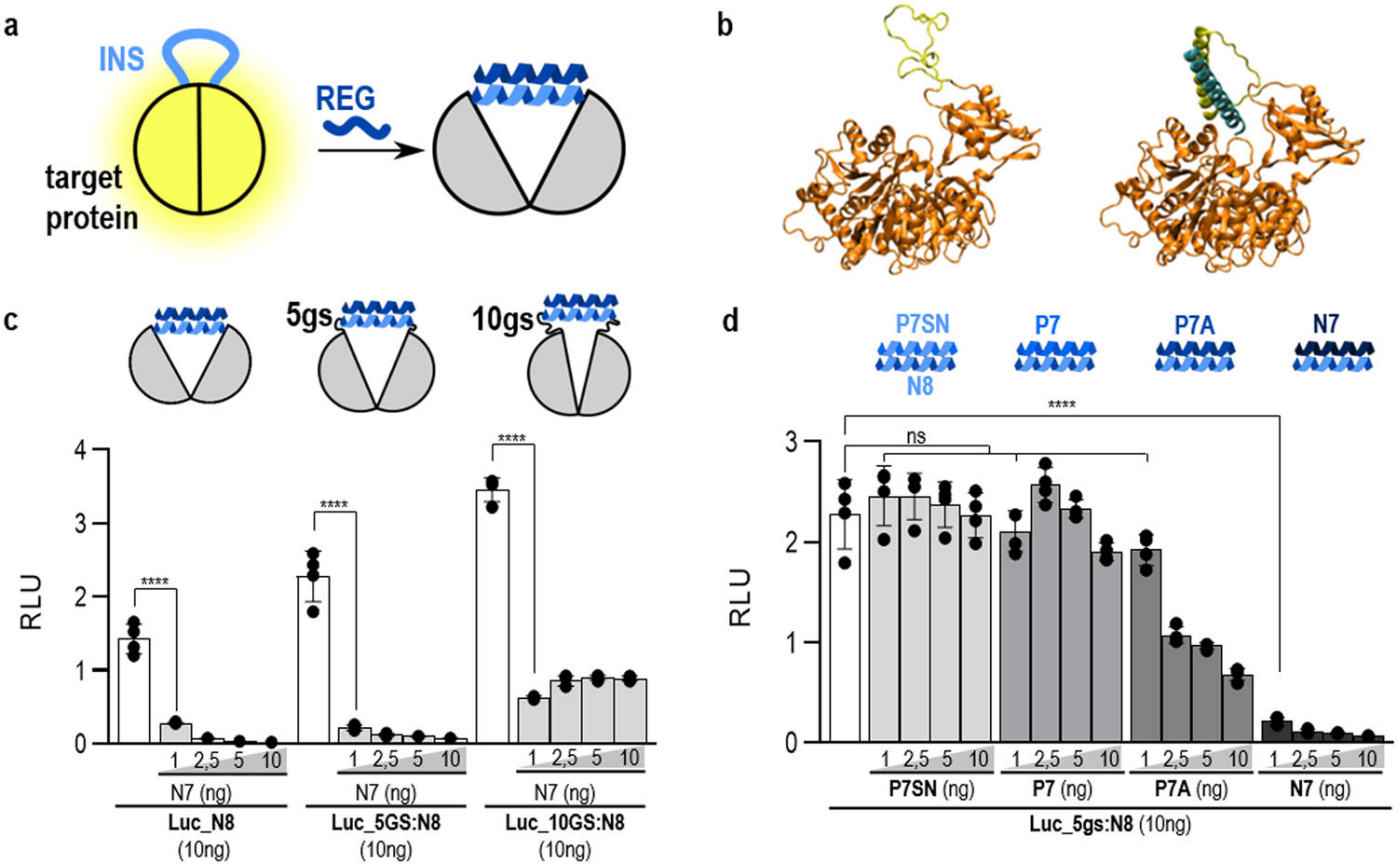
The principle of inserted peptide structure allosteric regulation (INSRTR) of protein function. **a** Scheme of the INRSTR principle, unstructured inserted peptide maintains protein structure and function, CC dimer formation is triggered by the regulatory peptide. INS — inserted peptide, REG — regulatory peptide. **b** Molecular model of INSRTR firefly luciferase that maintains its structure with insertion of the unstructured CC-forming peptide (inserted peptide) into the loop. The addition of a regulatory peptide leads to CC formation and thus structural changes in firefly luciferase leading to its inactivation. Both modeled by AlphaFold2; fLuc-firefly luciferase. **c** Peptide N8 was genetically fused into firefly luciferase with different lengths of connecting linker peptides on both sides of the peptide sequence. Linker regions allow for flexibility around peptides which affects firefly luciferase activity as well as the degree of inhibition with the addition of a complementary peptide. **d** The extent of inhibition depends on CC pair affinity, with the affinity rank N7:N8 > P7A:N8 > P7:N8 > P7N:N8. The values in (**b**, **c**) are the means of four biological replicates ± SD and representative of three independent experiments on plasmid transfected HEK293T cells. Significance was tested with one-way ANOVA with Dunnett’s multiple comparisons test between the INSRTR variant and the addition of a REG peptide; *P* values 0.1234 (ns), 0.0332 (*), 0.0021 (**), 0.0002 (***), < 0.0001 (****) (significance, confidence intervals, degrees of freedom, *F* and *P* values are listed in Supplementary Table S2).

To address this limitation, a broadly applicable and robust modular platform for the regulation of protein function is desired, which could be applied to diverse natural or engineered proteins and even implemented for logic circuits. Proteins tolerate a substantial degree of sequence and structure variability, particularly in solvent-exposed loops, and can often be genetically fused to other polypeptides while maintaining their function. Allosteric regulation^14,15^, has been introduced into individual proteins by mutations^16,17^, insertion of folded protein domains or domains regulated by small molecules^6,7,9,18^, however often with difficult-to-predict results. We reasoned that it might be possible to introduce a broadly applicable modular strategy of regulation, to disrupt only the local conformation of a protein at a site crucial for its function, by altering the conformation of a protein loop through CC dimer formation (Fig. 1a). In comparison to previously designed regulators, the inserted heterodimeric CC-based platform could have a unique ability to enable the construction of different types of protein logic functions, which may not be achievable for other inserted modules.

Here, we present a strategy based on peptide introduction into a permissible site of a host protein in a manner that should retain protein function. This is, in general, feasible because proteins often tolerate different loop lengths with divergent sequences. Upon binding of an appropriate binding partner of the inserted peptide, such as the complementary CC dimer-forming peptide, the inserted peptide adopts a helical conformation, expanding the distance between the inserted termini in the loop. This is sufficient to disturb the conformation at the site crucial for protein function, without the introduction of a large conformational or energetically expensive effect on the global fold. We have implemented this concept (named INSRTR for insertion of a peptide to regulate protein function) with designed CC dimer-forming peptides and we hypothesize this enables allosteric regulation of proteins. The broad applicability of this platform was demonstrated on ten different proteins comprising a range of protein folds, sizes, and functions. This includes enzymes, signaling kinases and mediators, DNA-binding proteins as transcriptional regulators, fluorescent proteins, and the single-chain variable fragment (scFv) of antibodies. For most of these proteins, designed regulation by inserted domains has not been reported before. Typically, several permissible insertion sites were identified for each tested protein, which retained their function and could be controlled in mammalian cells by a regulatory peptide delivered through several different modalities. To facilitate the selection of the insertion sites, a machine-learning algorithm was applied to identify the set of important descriptors. The scope of this platform was further extended by a genetic fusion with an intramolecular inhibitory peptide to invert the regulation from an OFF to an ON-switch. We additionally introduced regulation by small molecules that act on a chemically regulated split protease. Further extension of this strategy enabled the construction of the complete set of Boolean logic functions for the selected protein. The broad diversity of tested protein targets demonstrates a remarkable and robust potential to regulate biological processes and systems.

## Results

### The principle of inserted peptide for allosteric regulation of protein function (INSRTR)

The concept was explored in mammalian cells where its implementation could have an important impact on diverse applications. The insertion sites for each tested protein were initially selected based on the following considerations: (a) position within the solvent-exposed loop, (b) no direct involvement in the protein functional site, (c) preferentially variable length and sequence in orthologues within the target loops, (d) separation from the functional site by 1–4 nm and (e) proximity or structural connections between the insertion and functional site, which were later refined based on the experimental results and use of machine learning methods. To establish rules for this method that could be applied to other proteins, we tested the aforementioned considerations on firefly luciferase. Position 490 was selected, based on the 3D structure, separated 2 nm from the proposed active site^19^. Designed CC dimer peptide pairs, orthogonal to natural leucine zippers, with affinity in the nano- to the micromolar range, were used as allosteric regulators^20–22^. A CC dimer-forming peptide which is unstructured in the absence of its binding partner was inserted at a selected position (Fig. 1b, left). In the presence of the regulatory peptide, a CC dimer should form (Fig. 1b, right; Supplementary Fig. S1). The inserted peptide segment, therefore, adopts a helical conformation with a distance between its termini of ~4 nm. This disrupts the local structure of the protein, which could be relayed to the active site. Indeed, co-expression with the regulatory peptide resulted in the inhibition of luciferase activity (Fig. 1c). AlphaFold2 modeling and molecular dynamics simulation (MDS) of the luciferase model suggested that the formation of a CC dimer in the loop modified the conformation of the loop, while the rest of the host protein, including the geometry of the active site residues, remained essentially unperturbed (data not shown), which means no or low energy has to be compensated. Therefore, inhibition of the catalytic activity is the result of a subtle perturbation of the structure or/and dynamics of the active site. The in vitro modulation of the activity of INSRTR luciferase by the addition of synthetic peptides further demonstrates that binding of CC, and not an indirect effect on protein expression or degradation is crucial for the regulation of INSRTR-modified proteins (Supplementary Fig. S3a, b).

To optimize the ratio between active and inactive state in the absence or presence of a regulatory peptide, the length of the linker peptide between the CC-forming insert and an insertion site of the protein was varied. This revealed a five amino acid-residue linker as an optimal choice with up to 30-fold activity repression in the presence of the regulatory peptide (Fig. 1c). While the absence of a flexible linker strongly suppressed the activity of the active state, a longer linker, on the other hand, decreased inhibition, most likely because it decouples the conformational transition of the inserted peptide from the target protein conformation. The presence of a flexible linker adds to the importance of individual residues in the loop, which contributes to the robustness of INSRTR concerning the precise insertion site. Dimeric CC pairs have been designed in a wide range of stabilities^21,22^ and were shown to be orthogonal to the endogenous CC proteins in mammalian cells^20^; indeed, a comparison of four CC pairs revealed that the degree of inhibition was proportional to their affinities (Fig. 1d)^21,22^. Hence, the response of the allosteric switch could be tuned using a toolbox of designed CC heterodimers with a range of thermodynamic stabilities^21^.

### Design of protein activation and diverse delivery modalities of regulatory signals

**Fig. 2:**
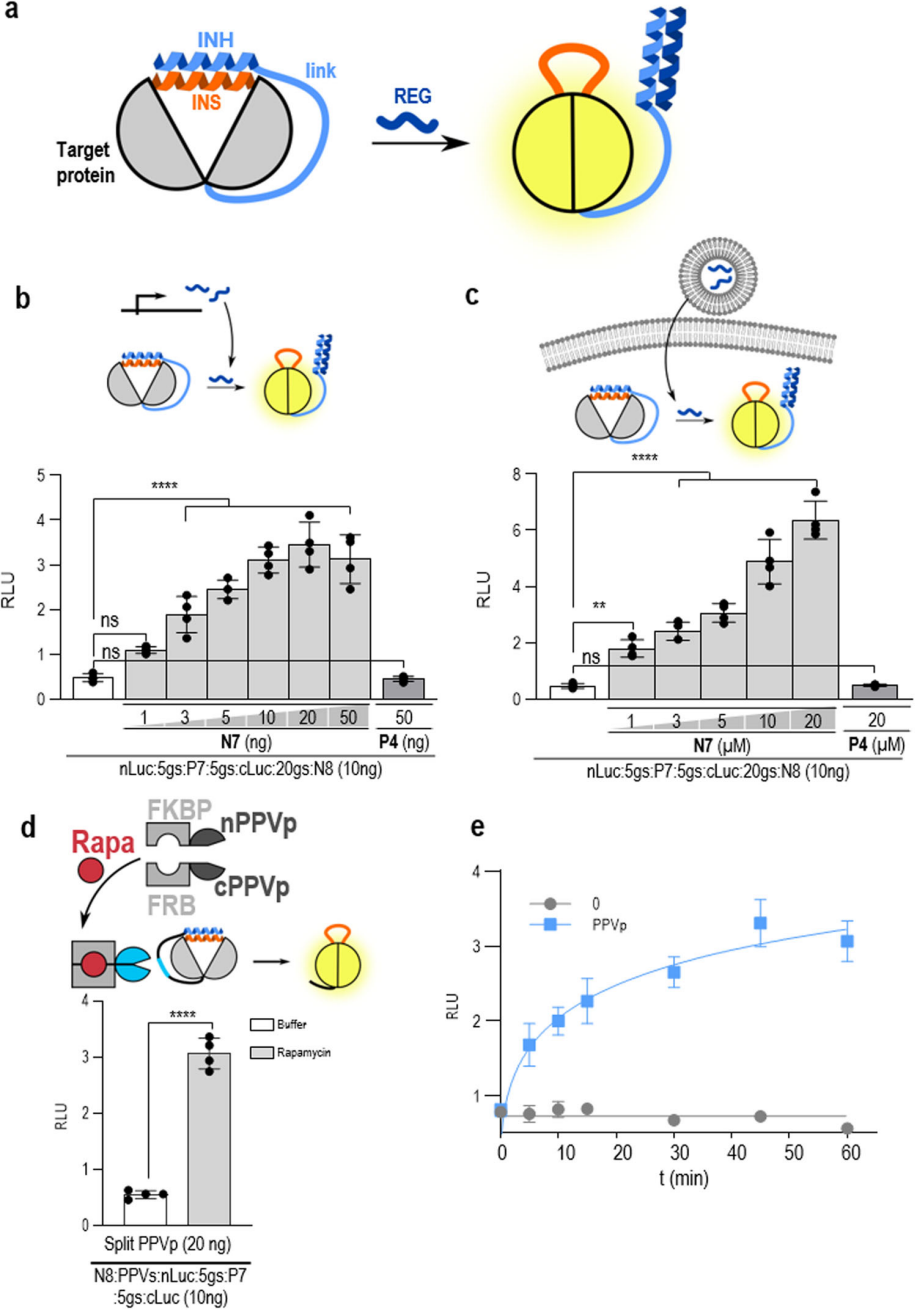
Construction of an ON-switch INSRTR with different triggers and kinetics of response in mammalian cells. **a** Schematic presentation of the inverted INSRTR (ON switch) design. The ON-INSRTR was designed by genetic fusion of a low-affinity CC peptide (inhibitory peptide — INH) to the C-terminus of the target protein with an inserted peptide in the protein loop (inserted peptide — INS). Due to the high intramolecular concentration, the inhibitory and inserted peptides form an intramolecular CC dimer despite low affinity, resulting in an inactivated target protein. The addition of the REG, with a high affinity for an inhibitory peptide, results in an unstructured inserted peptide, thus regaining target protein function. **b**, **c** Different N7 peptide delivery systems were evaluated for regulation of the ON-INSRTR system. The activity of ON-INSRTR firefly luciferase is upregulated with the (**b**) co-expression of REG peptide N7 or (**c**) an externally added peptide, forming the designed CC pair. The response is specific as the orthogonal CC-forming peptide P4 of the same length and similar composition does not affect firefly luciferase activity. **d** Coupling of chemical regulation to the INSRTR, through chemically activated protease regulation of ON-INSRTR switch, mediated by cleavage of the linker peptide between INH and the target protein. **e** Regulation of rapamycin-mediated reconstitution of PPV protease results in the fast kinetics of ON-INSRTR system activation. The values in (**b**–**d**) are the means of four biological replicates ± SD and representative of at least two independent experiments on transiently transfected HEK293T cells. Significance was tested with one-way ANOVA with Dunnett’s multiple comparisons test between the INSRTR variant and the addition of a REG peptide; *P* values 0.1234 (ns), 0.0332 (*), 0.0021 (**), 0.0002 (***), < 0.0001 (****) (significance, confidence intervals, degrees of freedom, *F* and *P* values are listed in Supplementary Table S2). INS-inserted peptide, INH-inhibitory peptide, REG-regulatory peptide, link-linker peptide, Rapa-rapamycin.

The INSRTR allows the inhibition of protein activity through the addition of a regulatory peptide. Often, a constitutively inactive protein is desired that could be activated by a selected signal. To design proteins that could be activated, an inverted, ON-INSRTR was constructed, where an inhibitory peptide with a weak affinity to the inserted peptide was genetically fused to the C-terminus of the host protein (Fig. 2a). This enabled the intramolecular binding of the inhibitory peptide to the inserted segment and a constitutively inactive state of a target protein. A regulatory peptide with a high affinity for the inhibitory peptide could therefore compete for binding and release the inserted peptide from an intramolecular dimer, thus restoring the activity of the host protein (Fig. 2a; Supplementary Fig. S2). This concept was demonstrated in firefly luciferase with an inserted P7 peptide and a C-terminal fusion of low-affinity N8 inhibitory peptide^22^. The formation of an intramolecular P7–N8 heterodimer resulted in an autoinhibited luciferase. The addition of a regulatory N7 peptide with a high affinity for N8 inhibitory peptide^22^ recovered luciferase activity (Fig. 2b, c). Further, several modes of regulation of ON-INSRTR were tested in mammalian cells, including the transcriptional regulation of a regulatory peptide (Fig. 2b) and an external delivery of the peptide to mammalian cells (Fig. 2c). An alternative option for the regulation of ON-INSRTR is the proteolytic cleavage of the linker between the host protein and the inhibitory peptide (Fig. 2d). For the latter, activation by the rapamycin-inducible heterodimerization between FKBP and FRB fused to the split plum pox virus protease (PPVp) was used^11^. In this setup rapamycin could activate the luciferase, where the specific cleavage site was inserted into the ON-INSRTR protein linking the C-terminal autoinhibitory CC segment (Fig. 2d). As a control, the use of P4 peptide as a regulatory peptide — which has a similar amino acid composition but a different electrostatic and hydrophobic motif than N8^22^ and therefore, does not bind to N8 — showed no effect on luciferase activity (Fig. 2b, c), in agreement with the orthogonality of the designed CC pairs.

Since the INSRTR relies on protein–protein interactions, it was expected that the addition of a regulatory peptide could trigger the response in mammalian cells much faster than transcriptional regulation. Indeed, luciferase activity was observed within 5 min of exposing living mammalian cells transfected with constructs for the ON-INSRTR and chemically regulated protease to rapamycin (Fig. 2e). In this manner, a small chemical trigger can be used to regulate the function of selected allosterically regulated proteins in cells, further supporting the proposed mechanism of INSRTR regulation.

### Construction of Boolean protein logic gates based on intramolecular interacting segments

**Fig. 3:**
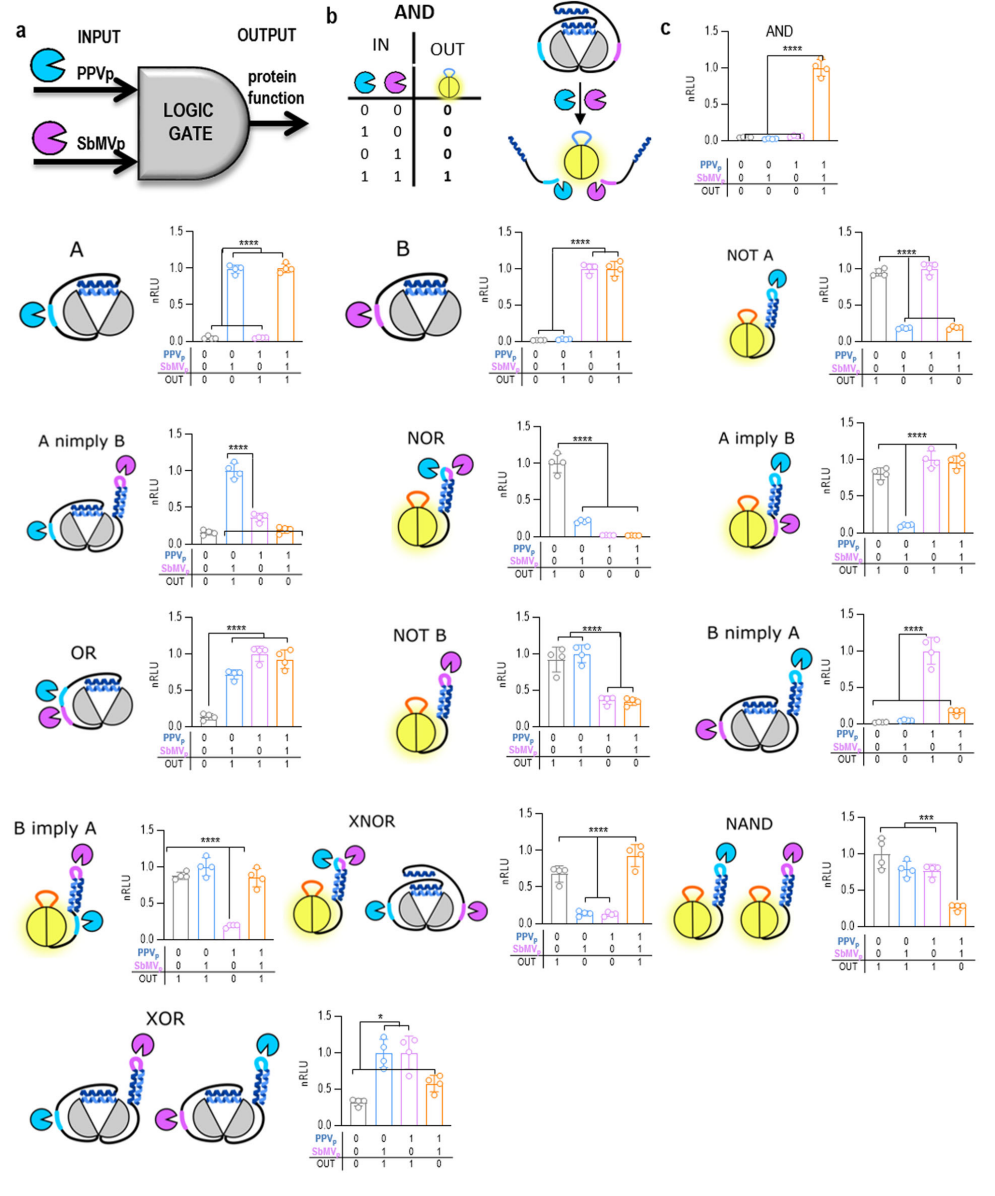
Construction of INSRTR protein logic gates based on intramolecular fusion with interacting and protease target segments. **a** Scheme of the implementation of INSRTR logic gates with protease inputs and allosterically activated protein function. **b** Schematic presentation of an AND logic gate. **c** Experimental testing of all nontrivial two-input Boolean logic functions by INSRTR in HEK293T cells, as regulated by a combination of PPV and SbMV protease inputs. The values in (**c**) are the means of four cell cultures ± SD and are representative of at least two independent experiments. Significance was tested with one-way ANOVA with Tukey’s comparison between indicated ON and OFF states. *P* values 0.1234 (ns), 0.0332 (*), 0.0021 (**), 0.0002 (***), < 0.0001 (****) (significance, confidence intervals, degrees of freedom, *F* and *P* values are listed in Supplementary Table S2).

The use of orthogonal and tunable CC modules for the regulation of protein function allowed us to further expand the concept of intra- and intermolecular interactions with regulatory CC segments and proteolysis to the construction of a full set of two input Boolean logic functions (Fig. 3a, b). This was accomplished by combining inhibitory peptides as extensions to both termini of the host protein and intramolecular interacting segments. The input signals were provided through orthogonal proteases, which cleave highly specific sites and whose activity can be regulated by small molecules. In the same manner, input signals could be also proteases specific to certain biological processes, such as caspases or viral proteases. We designed constructs with autoinhibitory CC peptides fused to both termini of firefly luciferase which represents an AND gate since cleavage by two proteases is required to remove the autoinhibition to generate an active output (Fig. 3b, c). By implementing designable CC peptides with appropriate affinities, and cleavage sites for orthogonal proteases at different positions and their combinations, we were able to implement all possible combinations of two-signal inputs with appropriate outputs (Fig. 3c; Supplementary Fig. S26). A distinct advantage of this strategy is that logic functions were genetically encoded within a single polypeptide chain.

### Selection of insertion sites based on machine learning algorithm

Selecting the optimal insertion site for allosteric regulation may involve as many as several tens of designs^16^ which need to be experimentally tested. The advantage of the INSRTR method is its robustness which results in a high success rate due to the addition of a flexible linker bordering the CC segment. Insertion site selection rules, as outlined above, are similar to the previously applied rules for insertion of light or rapamycin-responsive domains^9^. CC-based inserts are robust to the precise insertion site, as the insert is decoupled from the host protein fold due to flexible linkers, yet CC dimer structure formation introduces tension that is transduced to the active site. Nevertheless, we aimed to further facilitate the selection of the permissive insertion sites. We applied a machine learning algorithm to the experimental results on the effect of (the 47) peptide insertion sites in proteins with known 3D structure and their regulation by the regulatory peptide. We generated a large number of descriptors (see Supplementary File 1) which may play a role in the permissibility of sites for insertion and allosteric coupling to the functional site. The amount of experimental data (47 sites) is not sufficient to permit the use of deep neural networks for data analysis. We thus used Gradient Boosting Trees (GBT)^23^ to learn a predictive model for the outcome of peptide insertion. The model learnt by using the optimal parameter values has an accuracy of 66% and an area under receiver operator curve of 0.71 on unseen cases, as estimated by leave-one-out cross-validation (Supplementary Fig. S30). Besides the good predictive performance, the advantage of the specific machine learning approach used is its interpretability: it provides importance scores for the used descriptors based on physical reality, which enables understanding as well as identification of INSRTR sites for additional proteins. Indeed, the most important features are the solvent accessible surface of the loop and the distance to the active site.

The predictive model learned by the GBT algorithm is included in a publicly accessible online server (see Data availability). The server calculates the necessary descriptors used by the model and applies the model to make predictions. Based on these predictions, it suggests and ranks the sites appropriate for INSRTR for a selected protein 3D structural model, where users can upload an arbitrary PDB file. Even though the success rate for the proteins tested so far is good, machine learning will be improved further by using data on additional results of regulated proteins.

### Regulation of the activity of diverse protein types and functions in an OFF- or ON-INSRTR format

The most important and desired impact of INSRTR as a direct protein regulator platform would be the ability to control the function of a wide range of diverse proteins, which could open exciting possibilities to modulate biological systems, such as to temporally (in)activate transcription or epigenetic regulation, signaling pathway or excessive activation of therapeutic T cells.

**Fig. 4:**
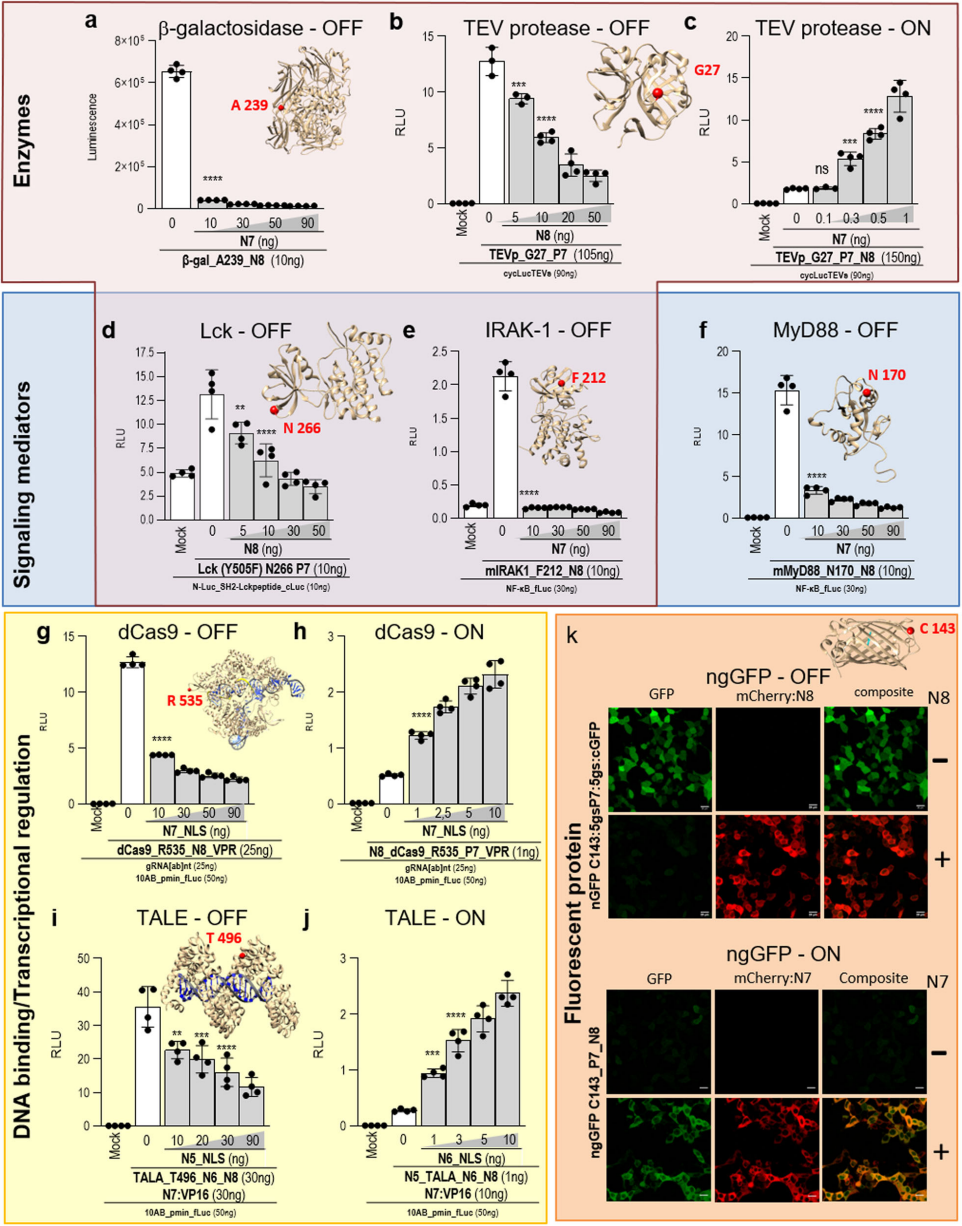
INSRTR regulation of diverse protein types and functions in an OFF or ON format. **a**–**k** Evaluation of INSRTR regulation on representative proteins from various protein groups, each tested for a corresponding specific protein functional assay. Enzymes: (**a**) β-galactosidase, (**b**, **c**) TEV protease, (**d**) Lck kinase; Signaling mediators: (**e**) IRAK1, (**f**) MyD88; DNA-binding-based transcriptional regulators: (**g**, **h**) dCas9, (**i**, **j**) TALE; and (**k**) Fluorescent protein GFP. Inserts are schematic presentations of allosterically regulated protein 3D structures with highlighted each peptide insertion site (red sphere). Mock represents transfection with reporter plasmids only. The values in (**a**–**k**) are the means of four biological replicates ± SD and representative of three independent experiments on transiently transfected HEK293T cells. Statistical significance was tested with one-way ANOVA with Dunnett’s multiple comparisons test between the INSRTR variant and the addition of a REG peptide; *P* values 0.1234 (ns), 0.0332 (*), 0.0021 (**), 0.0002 (***), < 0.0001 (****) (significance, confidence intervals, degrees of freedom, *F* and *P* values are listed in Supplementary Table S2).

To test and showcase the broad applicability of the INSRTR platform, we tested this system on ten different proteins/protein super-families with diverse biological functions, including enzymes (luciferase, protease, β-galactosidase, kinase), signaling proteins (signaling adaptor, kinase), transcriptional regulators based on DNA-binding domains (transcription activation-like effector (TALE) and dCas9/gRNA), fluorescent proteins, and antibodies. Among enzymes, in addition to luciferase, INSRTR was successfully implemented in a tobacco etch virus protease (TEVp), β-galactosidase, Lck, and IRAK1 protein kinases. For each enzyme several sites were tested for their applicability to INSRTR (Fig. 4a–e) and in each case at least one — but often up to three — sites were found to be amenable to the INSRTR platform (Supplementary Table S3). The activity of each protein was evaluated by the specific assay that was in most cases coupled to the corresponding luminescence output, such as proteolytic activation of the cyclic luciferase, reporter for transcription factor downstream of kinase or signaling adapter. For all tested enzymes, we successfully applied regulatory peptides to downregulate the activity (OFF) and, in most cases, also constructed an autoinhibited version that could be turned ON by the addition or co-expression of a regulatory peptide. An important group of proteins are mediators that regulate signal transduction in the immune response. This is achieved either through the protein kinase activity of Lck and IRAK1 kinase, the key mediators of T-cell activation and innate immunity, respectively, or as signaling adaptors recruiting other signaling components, such as MyD88. In the case of Lck and IRAK1 kinase, a peptide was inserted into the catalytic domain of the kinase. The addition of a regulatory peptide strongly inhibited the signaling function of the kinases (Fig. 4d, e). MyD88, a key innate immune signaling mediator, on the other hand, senses activation of Toll-like receptors and IL-1 receptors by binding to the membrane receptors through its Toll-interleukin receptor (TIR) domain. TIR domain in turn recruits downstream kinases by interaction through a death domain (DD)^24^. In the case of signaling adaptors that function through protein–protein interactions, signaling mediated through molecular assembly might be less sensitive to the CC-induced loop strain in comparison to the catalytic activity. Nevertheless, three INSRTR permissible sites within the TIR domain and one within a DD of MyD88 were identified, where the function of MyD88 was regulated by a peptide aimed to trigger disruption of the binding interface (Fig. 4f; Supplementary Figs. S13, S14). An additional biotechnologically important group of proteins are DNA-binding domains which act either through an RNA-mediated DNA recognition within the Cas9/gRNA complex^25^ (Fig. 4g, h; Supplementary Figs. S15-S17) or via DNA recognition by the protein domain (TALEs)^26^ (Fig. 4i, j; Supplementary Figs. S18-S20). These proteins can be designed to bind to almost any selected DNA sequence and represent versatile tools for genome editing, transcriptional and epigenetic regulation, and therefore the ability to regulate their activity is highly desirable. For both types of DNA-binding proteins, we successfully modulated DNA recognition by the regulatory peptide, demonstrated through the transcriptional output. We were able to prepare OFF as well as ON versions for both TALE or dCas9-mediated transcriptional activators (Fig. 4g–j). The green fluorescent protein is a versatile reporter for monitoring cells through microscopy, flow cytometry or other optical methods. Fluorescence of GFP has been regulated through several signals, e.g. calcium (Ca^2+^)^27^ or pH^28^ and here we demonstrate it could also be regulated through INSRTR. By the addition of a CC-forming peptide at position 143 (Fig. 4k, Supplementary Figs. S23–S25), we created a reporter.

### INSRTR for the regulation of chimeric antigen receptor (CAR) T cell response

**Fig. 5:**
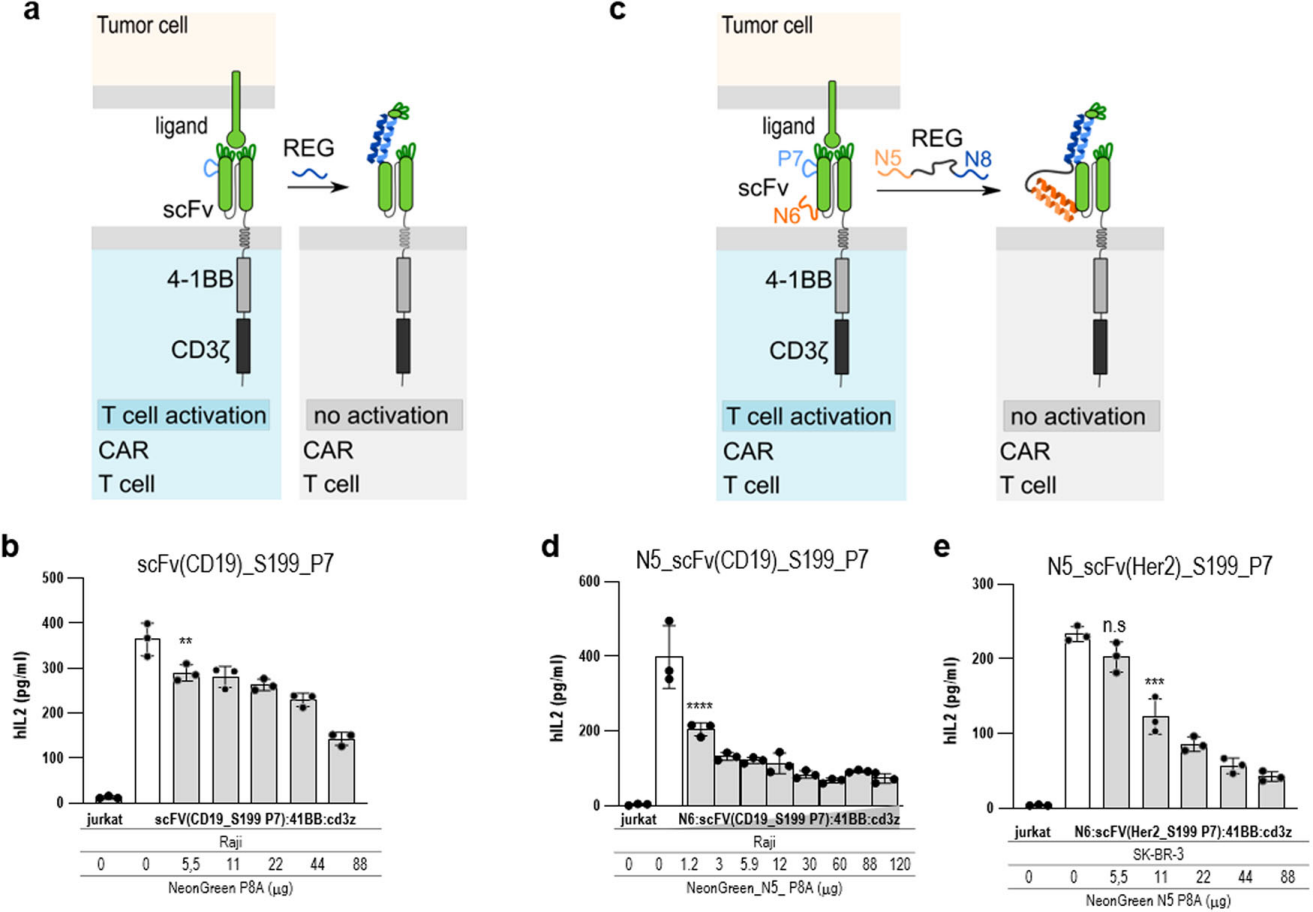
INSRTR regulation of CAR T cell activation. **a** Scheme of INSRTR-regulated CAR T receptor. P7 CC segment was inserted into a heavy chain of scFv at a loop neighboring CDR loops. **b** Antibody binding domain scFv CD19 N195 P7 in a CAR T receptor on cells activated by CD19-expressing Raji B cells. **c** Improved design of INSRTR CAR T receptor. An additional CC segment was fused to the N-terminus of the N195 P7 scFv(CD19) and a fusion peptide N5-P8A was used as a regulator. **d** Cells expressing N5_scFv(CD19)_N195_P7 were stimulated with Raji cells expressing CD19 antigen. **e** Antibody binding domain scFv Her2 S199 P7 in a CAR T receptor on cells activated by ErbB2-expressing SK-BR-3 cells. Statistical significance was tested with one-way ANOVA with Dunnett’s multiple comparisons test between the INSRTR variant and the addition of a REG peptide; *P* values 0.1234 (ns), 0.0332 (*), 0.0021 (**), 0.0002 (***), < 0.0001 (****) (significance, confidence intervals, degrees of freedom, F and *P* values are listed in Supplementary Table S2).

Antibodies play an important role in adaptive immunity and are widely used as therapeutics and reagents as they have been selected to detect or target almost any protein and many other molecules. Antibodies or their variable recognition domains are used for therapy alone or incorporated into different therapeutic devices. The ability to regulate the binding of antibodies to their targets, therefore, provides opportunities for research and applications. The variable domains of a single chain antibody (scFv) represent a targeting domain of CARs for the recognition of cancer-specific antigens and activation of therapeutic T cells as one of the important advances in cancer immunotherapy^29^. CAR T cells are typically used as a last resort therapy, as excessive activation of CAR T cells may lead to a cytokine storm that could have a lethal outcome^30^. Therefore, it would be desirable to have the option to rapidly and transiently desensitize CAR T cells rather than eliminate them in case of excessive activation. Several methods of regulation of scFv binding have been invented based on masking the binding site^31^ or through small ligands, which had to be optimized for each scFv domain separately through screening^32^. The INSRTR therefore provides a technology to regulate binding to targets. Insertion of a CC-forming peptide into the loop adjacent to the complementarity-determining regions (CDR) loops of the heavy chain of the CD19 recognition domain of scFv retained binding and activation of T cells in the presence of CD19-expressing target cells by INSRTR-CAR T cells (Fig. 5a). While the effect was significant, the addition of a regulatory peptide only partially suppressed the IL-2 production (Fig. 5b; Supplementary Fig. S21a, b). We aimed to improve the efficiency of CAR regulation by increasing the affinity of the regulatory peptide for scFv. INSRTR-CAR scFv (N195 P7) was modified with an additional fusion of an orthogonal CC-forming peptide (N6) to the N-terminus. The regulatory peptide comprised the N8 peptide, which binds to the inserted peptide (P7), and the N5 peptide, which is complementary to the N6, fused to the N-terminus of the scFv (Fig. 5c). In this manner, the regulatory peptide can bind to the scFv of the CAR through N5–N6 interaction, regardless if the scFv is already engaged with the antigen. The binding of the regulatory peptide to the ectodomain of CAR increases the local concentration of the N8 segment that competes for binding to the inserted peptide segment. Indeed, the addition of a regulatory peptide composed of N8 and N5 led to strong suppression of the IL-2 production by INSRTR CAR T cells (Fig. 5d; Supplementary Fig. S21c, d). To test the applicability of the INSRTR platform to other scFv for regulation of other CARs, we designed and tested a second, α-Her2 INSRTR-regulated scFv. Similar to the results on anti-CD19 scFv, the insertion between the D and E strands of the heavy chain, adjacent to the antigen-binding CDRs was found to be optimal. Insertion of a P7 peptide at position 199 of the α-Her2 scFv retains its function (Fig. 5e; Supplementary Fig. S21e, f), while the addition of a regulatory peptide strongly suppressed the IL-2 production. The INSRTR platform could therefore be implemented to inhibit therapeutically relevant CARs (Fig. 5; Supplementary Figs. S21, S22) and likely be applied for other applications employing antibodies or their fragments.

## Discussion

INSRTR represents a broadly applicable platform for the regulation of selected proteins based on the formation of CC dimers within the insertion sites. Designed CC dimers have several unique advantages. They enable the construction of rational activation (ON-INSRTR) or inactivation (OFF-INSRTR) of target proteins as well as the design of logic gates for the regulation of the selected protein function. This report presents the first two-input logic gate implementation of allosteric protein regulation. This is possible due to the well-understood and adjustable properties of designed coiled coils and their orthogonality in mammalian cells^20^. The designed CC dimers incorporate negative design principles, are orthogonal in mammalian cells and more robust concerning the oligomerization state than some extensively studied natural CCs, such as GCN4, making their response more predictable^20,22^. Selection of the insertion position demonstrated a high success rate, most likely due to the presence of flexible linkers which decrease sensitivity to a particular structure and residues at the insertion site and well-defined properties of CC dimers. Moreover, the use of machine learning algorithm further improves the predictability of this strategy and enables access to this technology to the broader scientific community.

Implementation of INSRTR into the ten diverse and unrelated proteins demonstrated the robustness of this platform (Figs. 4, 5). While the activity of engineered proteins varied across the selected position of insertion, we were able to prepare at least one variant for each protein with strong inhibition triggered by the regulatory peptide (Supplementary Table S3). Importantly, for many proteins tested in this study, the insertion of CC-based regulation has not been reported before. The use of chemically regulated proteases facilitates the regulation of diverse proteins by small molecules (Fig. 2; Supplementary Figs. S28, S29). INSRTR enabled rapid response in mammalian cells through direct control of protein function which can be extended to the construction of interaction networks and logic operations.

This platform requires a small genetic footprint and is expected to have low immunogenicity owing to the designable features of CC peptides, particularly at solvent-exposed positions^21,33,34^. Implementation of INSRTR requires low-effort engineering and has so far exhibited a high success rate (Supplementary Fig. S27 and Table S3). In several proteins, INSRTR permitted several insertion sites which could bypass interferences at particular loops. The regulation of INSRTR can be implemented through transcriptional activation, small molecules, or the addition of an external peptide. The ON or OFF adaptation of a target protein and intramolecular fusion with regulatory peptides offer the possibility to engineer complex regulatory circuits with a fast response.

We have not been able to determine high-resolution structure of any INSRTR protein, as the region of insertion was not well defined in the cryoelectron structure reconstruction of the β-galactosidase (data not shown) and proteins could not be crystalized, most likely due to the high mobility at the insertion site. Nevertheless, in vitro activation or inhibition of protein function strongly supports regulation mechanism based on allostery. In vitro regulation of INSRTR_Luc in cell lysates as well as CAR regulation by the extracellular addition of a regulatory peptide are the clearest indicators of allosteric regulation, which rules out the mechanism through the cellular degradation machinery. However, given the large diversity of investigated proteins, the contribution of other cellular processes to protein function could not be excluded. Further, achieving both activation and inhibition of the same protein confirms that the observed effect is not the result of steric interference, and the response was observed also on enzymes with small substrates and several insertion sites remote from the active site.

Insertion of folded domains^7^ for protein regulation, with proteins such as FKBP, DHFR, ubiquitin^8,16,35^, through light-inducible domains^36^ or alternate frame folding^37^ has already been reported. However, the effect of domain insertion often requires a very precise site of insertion and results were frequently unpredictable^38^ and may require an extensive library screening^16^, while the modular principle of INSRTR identified many new sites permissible for regulation. Importantly, the use of CC segments with a set of well-defined competing peptides and orientation provides a unique ability to introduce more complex types of regulation.

Some hub proteins that interact with many partners through several sites^17^ or intrinsically disordered proteins might be difficult to engineer, nevertheless, demonstration of regulation of MyD88 as a central signaling mediator of Toll-like receptor signaling involved in the multi-protein signaling complex has been successful.

It has been previously shown that CC interactions can be regulated by competitive binding, antibodies, metal ions^39,40^, phosphorylation, and proteolysis^11^; therefore, the INSRTR platform could be extended to be responsive to additional signals and processes. Besides mammalian cells, the design principles and mechanism should facilitate the application of INSRTR in diverse biological or in vitro systems. The broad range of protein structures and functions admissible for regulation by INSRTR opens up a range of possibilities for regulating biological processes with potential applications for biotechnology or therapy as demonstrated in regulation of cancer-targeting CAR T cells.

## Materials and methods

### MDS

To gain insight into the structural dynamics of INSRTR structures, we performed MDS with a luciferase protein model using CHARMM27 all-atom force field embedded in GROMACS simulation package^41^.

INSRTR models were prepared by inserting a loop into a wild-type protein fold (PDB ID: 1BA3) using MODELLER^42^. Protein models with the CC structure were generated by extending the loop using the GROMACS pull code. Umbrella pulling was applied between two residues representing the beginning and the end of a helix. Upon reaching the distance of 4 nm, the CC structure was modeled on the loop backbone. For all the structures — a solvated and electroneutral system was assembled, followed by energy minimization, and equilibration under NVT (Constant number of particles, Volume, and Temperature) and NPT (Constant number of particles, Pressure, and Temperature) conditions. Equilibrated structures of luciferase were simulated for 5 ns and final MD trajectories were analyzed using GROMACS and Chimera^41,43^.

### AlphaFold2 modeling

Protein models were built using publicly available scripts and the modeling algorithm AlphaFold2^44,45^. If AlphaFold2 was not able to generate a structure due to its size or the structure had the wrong pairing of CC, we prepared the models using MODELLER^42^.

### Training of INSRTR prediction model

Gradient boosting is an ensemble method that combines several weak learners, usually decision trees, to produce a powerful predictive model. Gradient Boosting for classification is implemented in Scikit-learn’s GradientBoostingClassifier^46^.

The GBT ensemble method builds several models with weak performance and combines them to achieve high predictive performance. We optimized the values of three parameters of GBT, namely learning_rate, min_samples_split (minimum number of samples required to split an internal node) and n_estimators (the number of models), by performing grid search, using internal leave-one-out cross-validation (LOOCV), and taking the majority vote on the best parameters chosen in each fold.

We trained a Gradient Boosting Classifier with the binary log-loss (“log-loss”) function used as the optimization criterion for binary classification. The tuning of the hyperparameters was done using LOOCV on 80% of the data, with 20% of it being kept as a separate testing set. Once the hyperparameters were set, LOOCV was done on the entire dataset to evaluate the performance of the tuned model.

Accuracy and Area Under the Curve (AUC) were used to measure the performance of the model. The accuracy of the model is 0.66. The AUC score is 0.71.

Features were ranked using the permutation feature importance algorithm as implemented in scikit-learn. The importance is defined as the decrease in a model score when a single feature value is randomly shuffled.

The INSRTR package uses the following libraries: python 3.10, pdb-tools^47^ 2.4.0, mdtraj^48^ 1.9.7, numpy^49^ 1.22.4, biopython^50^ 1.81, scikit-learn^46^ 1.2.2, pandas^51^ 1.5.3, alphafold^44^ 2.3.2, colabfold^45^ and py3dmol 2.0.1.post1.

### Plasmids and cell lines

All plasmids (listed in Supplementary Table S1) were constructed using the Gibson assembly method. The human embryonic kidney cell line, HEK293T (ATCC CRL-3216), was cultured in complete media (DMEM; 1 g/L glucose, 2 mM L-glutamine, 10% heat-inactivated FBS (fetal bovine serum; Gibco)) with 5% CO_2_ at 37 °C. We used plasmid pcDNA3 (Invitrogen) to express INSRTR proteins. *Renilla* luciferase (phRLTK, Promega) was used as a transfection control in the dual luciferase assay. Jurkat, Raji, and SK-BR-3 cell line were cultured in complete media (RPMI 1640 W/GLUTAMAX-I, Gibco, 10% heat-inactivated FBS (Gibco)) with 5% CO2 at 37 °C.

### INSRTR protein regulation

HEK293T cells were seeded in 96-well plates (Corning) at 2.5 × 10^4^ cells per well (0.1 mL). The next day, cells were transiently transfected with plasmids expressing split proteins (sequences are shown in Supplementary Table S1), and phRL-TK (Promega) constitutively expressing *Renilla* luciferase (5 ng per well, to normalize transfection efficiency) using the PEI transfection reagent. The total amount of DNA for each transfection was kept constant by adding appropriate amounts of the control plasmid pcDNA3 (Invitrogen). For CC-regulated protein activity, HEK293T cells were transfected with plasmids expressing mCherry with C’- terminally fused CC peptide under cytomegalovirus (CMV), and phRL-TK (Promega) constitutively expressing *Renilla* luciferase (5 ng per well) using PEI transfection reagent. The synthetic peptides were transfected into cells using a DOTAP transfection reagent according to the manufacturer’s instructions. Briefly, 24 h after plasmid transfection, peptides were diluted in Hepes buffer to the working concentration of 200 μM and then further diluted to the final concentrations used on cells. The DOTAP transfection reagent was added to the peptide solution and transferred to the medium after 15-min incubation. Six hours later, the media was removed, and the cells were lysed using 1× Passive lysis buffer.

The kinetics curves (Figs. 2e, 3d) were fit to Velocity as a function of the substrate (Nonlinear regression; allosteric sigmoidal) using GraphPad Prism 8 (Y=Vmax*X^h/(Khalf^h + X^h)).

Jurkat cells were electroporated using the Neon Electroporation System (Life Technologies). Before electroporation, cells were washed with PBS and resuspended in R buffer at a density of 3 × 10^7^ cells per mL. 100 μL cells were mixed with 10 μg of plasmid DNA encoding the INSRTR variant of CD19 or Her2 CAR and electroporated (1600V, 3 pulses, 10 ms). Immediately after pulsing, cells were transferred to one well of a 12-well plate containing a pre-warmed complete RPMI 1640 medium. The next day, 24 h post electroporation, cells were counted and seeded in 96-well plates with corresponding target cells (Raji for CD19 CAR and SK-BR-3 for Her2 CAR) at a ratio of Effector (Jurkat-CAR-T): Target (Raji/SK-BR-3) = 5:1 and increasing amounts of a protein with inhibitory CC segment were added. Stimulation with target cells was terminated after 24 h by the removal of media. Media was used for enzyme-linked immunosorbent assay (ELISA) detection of hIL-2, produced as a result of Jurkat CAR-T cell activation.

### Flow cytometry

Flow cytometry was used to determine expression levels of INSRTR-modified CAR T variants on Jurkat cells. Cells were washed two times in buffer (2% BSA in PBS) before cell surface stained with fluorescent-labeled antibody (anti-c-Myc 9B11 Alexa Flour 647 or anti-c-Myc 9B11 Alexa Flour 488). Cells were resuspended in 100 μL buffer containing antibody diluted 1:200 and incubated at 4 °C for 30 min. Cells were washed twice with PBS and analyzed on a Cytek Aurora running SpectroFlo software. Flow cytometry data were analyzed with FlowJo v 10.8.1 software.

To determine the effect of the addition of CC pair (NeonGreen_N5_P8A) protein on the expression of CAR on the cell surface, 1 × 10^6^ Jurkat cells expressing CAR (N6_scFc_CD19_195_P7) were washed in buffer (2% BSA in PBS) and incubated with different amounts of NeonGreen for 1 h at 4 °C. Cells were then stained with a fluorescent-labeled antibody (anti-c-Myc 9B11 Alexa Flour 647) for 30 min at 4 °C. Cells were washed twice with PBS and analyzed on a Cytek Aurora running SpectroFlo software. Flow cytometry data were analyzed using FlowJo v 10.8.1 software.

### Confocal microscopy

For confocal microscopy, HEK293T was transiently transfected with plasmids encoding INSRTR GFP and/or mCherry: CC. 48 h after transfection, cells were analyzed. Microscopic images were acquired using the Leica TCS SP5 inverted laser-scanning microscope on a Leica DMI 6000 CS module equipped with an HCX Plane-Apochromat lambda blue 63× oil-immersion objectives with NA 1.4 (Leica Microsystems, Wetzlar, Germany). A 488-nm laser line of a 100-mW argon laser with 10% laser power was used for GFP excitation, and the emitted light was detected between 500 and 540 nm. A 1-mW 543-nm HeNe laser was used for mCherry: CC excitation and emitted light was detected between 580 and 620 nm. The images were processed with LAS AF software (Leica Microsystems) and ImageJ software (National Institute of Mental Health, Bethesda, Maryland, USA).

### Dual luciferase assays

At the indicated time points, the cells were lysed in Passive Lysis 1× Buffer (Promega) and analyzed with a dual-luciferase reporter assay to determine the firefly luciferase and the *Renilla* luciferase activities (Orion II microplate reader, Berthold Technologies). Relative luciferase units (RLU) were calculated by normalizing the firefly luciferase value to the constitutive *Renilla* luciferase value in each sample. Normalized RLU (nRLU) values were calculated by normalizing the RLU values of each sample to the value of the indicated sample within the same experiment.

### ELISA

ELISA test was performed to determine secreted hIL2 from electroporated and stimulated Jurkat cells. High-binding half-well plates (Greiner) were used. Human IL-2 was measured using a standard ELISA assay according to the manufacturer’s protocol (hIL-2 ELISA Invitrogen 88-7025-88). In brief, plates were coated with primary antibody and incubated overnight (4 °C). Next day plates were washed with PBS + 0.05% Tween20 using an ELISA plate washer (Tecan). Next, plates were blocked for 1 h at RT with ELISA diluent (PBS + 3% FBS) solution. Afterwards, the plates were again washed. The serial dilution of hIL2 standard and 1:2 diluted samples were added and incubated at RT for 2 h. Next, plates were washed and afterward, a detection antibody was added. Plates were incubated for 1 h at RT. Next plates were washed and HRP-conjugated avidin was added and incubated for 30 min. After the addition of substrate (TMB solution) the reaction was stopped with 0.16 M sulfuric acid. The plates were read on a microplate reader at 450 nm, and again at 630 nm for correction by subtraction of the reading at 630 nm from that at 450 nm.

### Beta-galactosidase assay

HEK293T cells were transiently transfected with plasmids expressing β-galactosidase variants and N7 peptides using the PEI transfection reagent. Enzymatic activity of β-galactosidase was assessed with β-Gal Reporter Gene Assay, chemiluminescent (Roche, cat. no. 11758241001) according to the manufacturers’ instructions. Briefly, 24 h after transfection media was removed and cells were lysed with Lysis reagent. After 30 min incubation, the substrate was added for additional incubation of 30 min in the dark. Initiation of chemiluminescent reaction was achieved with automatic injection of Initiation solution with Orion II microplate reader (Berthold Technologies) followed by luminescence readout. Enzymatic activities of β-galactosidase variants were plotted against the standard curve of recombinant β-galactosidase from *Escherichia coli*.

### Immunoblotting

HEK293T cells were seeded in 6-well plates (Techno Plastic Products) at 2.5 × 10^5^ cells per well (1 mL). The next day, the cells were transiently transfected with plasmids expressing CCs and/or INSRTR luciferase. The total amount of DNA for each transfection was 2.5 µg. 48 h after transfection, the cells were washed with 1 mL PBS and lysed in 100 µL of lysis buffer (40 mM Tris-HCl, pH 8.0, 4 mM EDTA, 2% Triton X-100, 274 mM NaCl with a cocktail of protease inhibitors (Roche)). Cells were incubated on ice for 10 min and then centrifuged for 15 min at 17,400 rpm at 4 °C to remove cell debris. The total protein concentration in the supernatant was determined using a BCA assay. Proteins from the supernatant were separated on 15% SDS-PAGE gels (120 V, 60 min) and transferred to a nitrocellulose membrane (350 mA, 30 min). Membrane blocking, antibody binding, and membrane washing were performed with an iBind Flex Western device (Thermo Fisher) according to the manufacturer’s protocol. The primary antibodies were mouse anti-Myc (Cell signaling technologies; diluted 1:2000), rabbit anti-HA (Sigma H6908; diluted 1:2000), and mouse anti-β-actin (Cell Signaling 3700; diluted 1:2000). The secondary antibodies were HRP-conjugated goat anti-rabbit IgG (Abcam ab6721; diluted 1:3000) and HRP-conjugated goat anti-mouse IgG (Santa Cruz, sc2005; diluted 1:3000). The secondary antibodies were detected with Pico or Femto Western blotting detection reagent (Super Signal West Femto; Thermo Fisher) according to the manufacturer’s protocol.

### Protein purification

*E. coli* NiCo21 (DE3) strain (New England Biolabs) was transformed with constructs cloned in pET41a expression vector and grown at 37 °C overnight (160 r.p.m.) in Luria-Bertani (LB) medium containing kanamycin (50 μg/mL). Bacterial cultures were transferred to LB medium at OD of 0.1, grown at 37 °C until OD reached 0.6, and induced with 0.5 mM IPTG. After induction, the cultures were cultured for 4 additional hours at 30 °C. Cells were then harvested by centrifugation, pellets were frozen at –20 °C overnight. Harvested cells were resuspended and lysed on ice with a lysis buffer: 50 mM Tris buffer at pH 8, 150 mM NaCl, 0.5 mg/mL Lysozyme (Sigma-Aldrich), 15 U/mL Benzonase (Millipore), and CPI protease inhibitor mix (Sigma-Aldrich). Cell lysis was completed by ultrasonication on ice for 6 min, at intervals of 1 s pulse and 3 s pause (45% amplitude). Subsequently, cellular lysates were centrifuged at 16,000× *g* (4 °C) for 20 min, and respective soluble fractions were filtered through 0.2-μm filter units (Sartorius) and applied to Ni-NTA resin (Golden Biotechnology) previously equilibrated with buffer (50 mM Tris buffer at pH 8.0, 150 mM NaCl, 10 mM Imidazole). After washing with buffers A and B (50 mM Tris buffer at pH 8.0, 150 mM NaCl, 20 mM imidazole), the bound fraction was eluted with buffer C (50 mM Tris buffer at pH 8.0, 150 mM NaCl, 250 mM imidazole). Glycerol (10% v/v) was added to the eluted fractions. The samples were then concentrated (Millipore centrifugal unit 3,5 MWCO), characterized by SDS-PAGE, and shock-frozen in liquid nitrogen and stored at −80 °C.

### Chemical inducers

A/C Heterodimerizer (rapalog, AP21967; Clontech Laboratories, Inc., part of Takara Bio USA, Inc.) was dissolved in dimethyl sulfoxide (Sigma Aldrich) at 1 mM concentration. Before stimulation, a stock solution of rapalog was diluted in DMEM medium (Invitrogen) to the final 1 µM concentration and added to each well in 96-well plates. HEK293T cells were stimulated with rapalog 24 h post-transfection and transferred to CellInsight CX7 LZR High-Content Screening (HCS) Platform (Thermo Fisher) with incubation chamber set to 37 °C, 5% CO_2_ and 70% humidity. Cells were monitored for 24 h post-induction with picture acquisition at nine fields of interest within one well every 30 min by excitation with a 488 nm laser at 20× magnitude. Pictures were analysed with CellInsights’ provided HCS Studio and quantified, producing reported relative fluorescence units (RFU). RFU present an average of four well replicates.

Doxycycline (Sigma Aldrich) was dissolved in MQ at 5 mg/mL concentration. Before the experiment, doxycycline was further diluted in DMEM to the final concentration of 100 ng/mL used for the stimulation. HEK293T cells were stimulated with chemical inductors 24 h post-transfection and cultivated for an additional 24 h. Further cells were lysed and measured for luminescence on a Centro LB 963 microplate reader (Berthold Technologies).

### Statistical analysis

All statistical analyses were performed using GraphPad Prism 8. All experiments were independently performed in triplicate unless otherwise indicated. The detailed statistic is listed in Supplementary Table S2. All experiments showing representative data were repeated at least twice with similar results. Independent experiments refer to independent cell samples seeded, transfected, treated, and analyzed on different days.

### Supplementary information

Supplemental material

Supplementary File S1

Supplementary table and file legend

## Data Availability

The authors declare that the data supporting the findings of this study are available within the paper and Supplementary information files. All other data are available from the corresponding author upon reasonable request. Code and data for the INSRTR prediction are available at https://github.com/ajasja/INSRTR. The code can be run online as a Google Colab notebook at https://colab.research.google.com/github/ajasja/INSRTR/blob/main/INSRTR.ipynb.
