# Supplementary material for: Designed allosteric protein logic
Source: Cell Discov. 2024 Jan 16;10:8. doi: 10.1038/s41421-023-00635-y (PMC10791696; doi:10.1038/s41421-023-00635-y)
Supplement: Supplementary file 1 — Supplemental material [file 41421_2023_635_MOESM1_ESM.pdf]

Supplemental information

Designed allosteric protein logic

Tjaša Plaper[1,*], Estera Merljak[1,*], Tina Fink[1,*], Tadej Satler[1,2], Ajasja Ljubetič[1], Duško Lainšček[1], Vid Jazbec[1,2], Mojca Benčina[1,3], Sintija Stevanoska[4], Sašo Džeroski[4], and Roman Jerala[1,3, †, #]

[1]Department of Synthetic Biology and Immunology, National Institute of Chemistry, Hajdrihova 19, SI-1000 Ljubljana, Slovenia,

[2]Interdisciplinary doctoral study of biomedicine, Medical Faculty, University of Ljubljana, 1000 Ljubljana, Slovenia.

[3]Centre for Technologies of Gene and Cell Therapy, Hajdrihova 19, SI-1000 Ljubljana, Slovenia,

[4]Department of knowledge technologies, Jožef Stefan Institute, Jamova cesta 39, 1000 Ljubljana, Slovenia.

SUPPLEMENTAL FIGURES

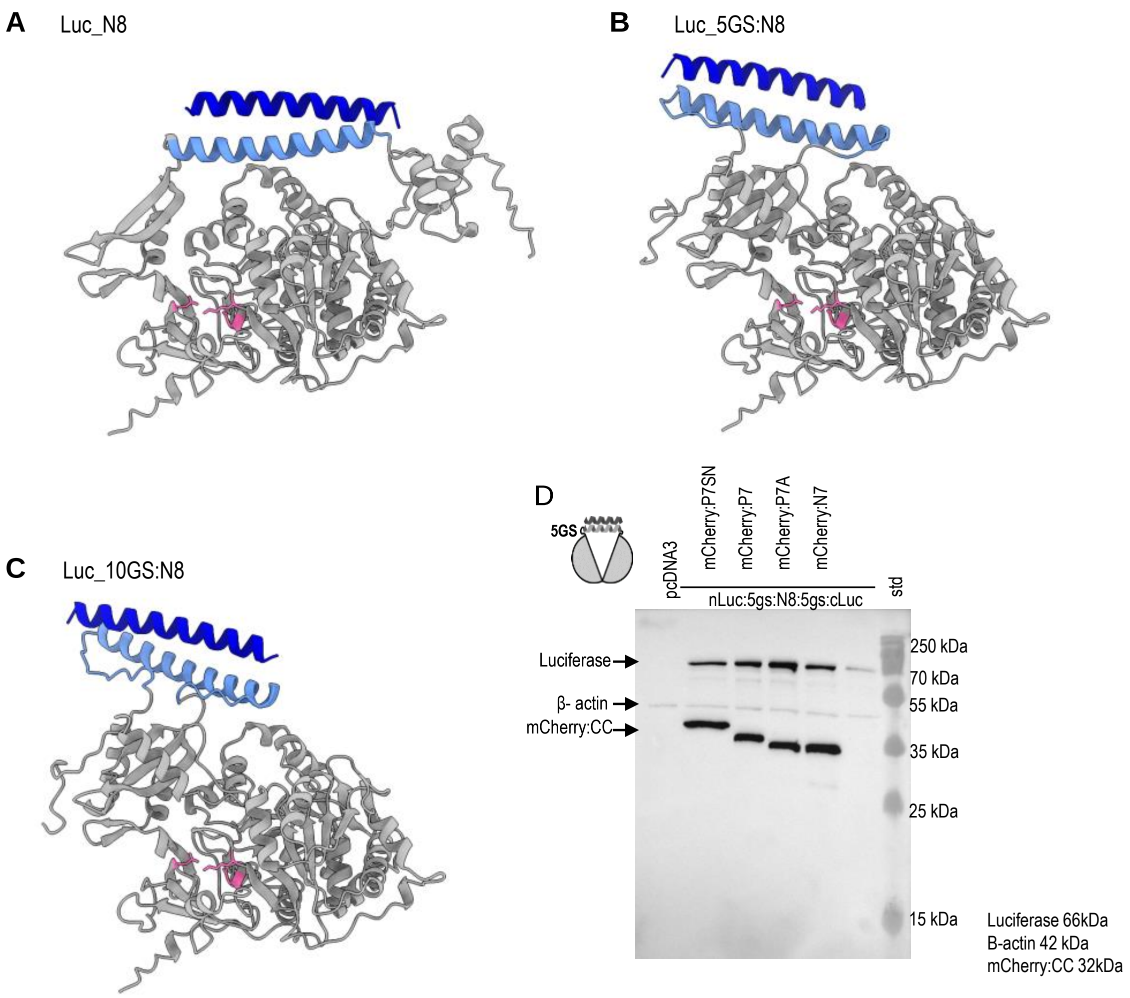

Supplementary Fig. S1 – a molecular model of INSRTR firefly luciferase (OFF switch). Firefly luciferase with unstructured inserted peptide (INS; light blue) maintains structure and function, and coiled-coil formation triggered by the regulatory peptide (REG; dark blue) deactivates luciferase activity. A) fLuc with INS peptide N8. B) fLuc with INS peptide N8 and 5GS linkers on either side. C) fLuc with INS peptide N8 and 10GS linkers on either side. The proposed active site (Lys204, Glu342, and Asp420) is shown in magenta. D) Western blot analysis of expression of INSRTR OFF luciferase and coiled-coil peptides. Coiled - coils were expressed as a fusion with mCherry protein for better expression.

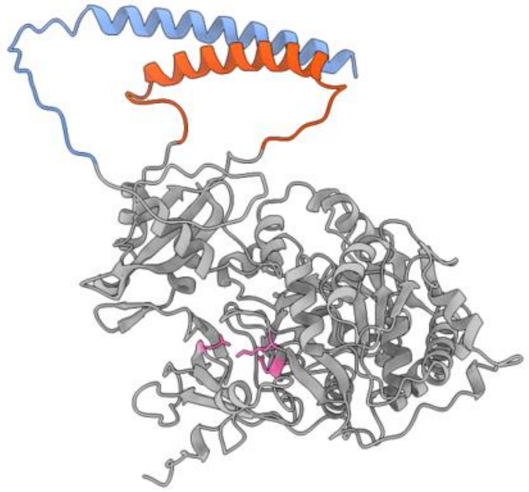

Supplementary Figure S2 – a molecular model of inverted INSRTR firefly luciferase (ON switch).
Firefly luciferase with unstructured inserted peptide (INS; orange) and inhibitory peptide (INH; light blue). The proposed active site (Lys204, Glu342, and Asp420) is shown in magenta.

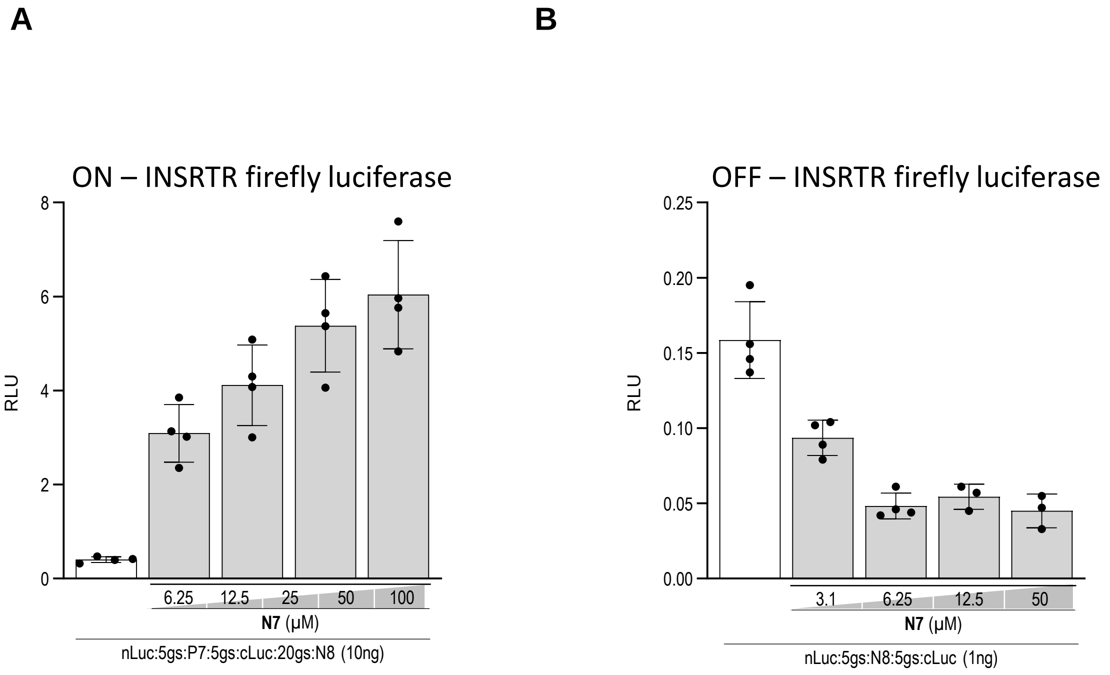

Supplementary figure S3 - *In vitro* regulation of both ON- and OFF-INSRTR firefly luciferase by a synthetic CC peptide. (A) ON – INSRTR firefly luciferase from cell lysate regains its function upon the addition of a peptide N7, **(B)** OFF – INSRTR firefly luciferase is inhibited upon the addition of a peptide N7.

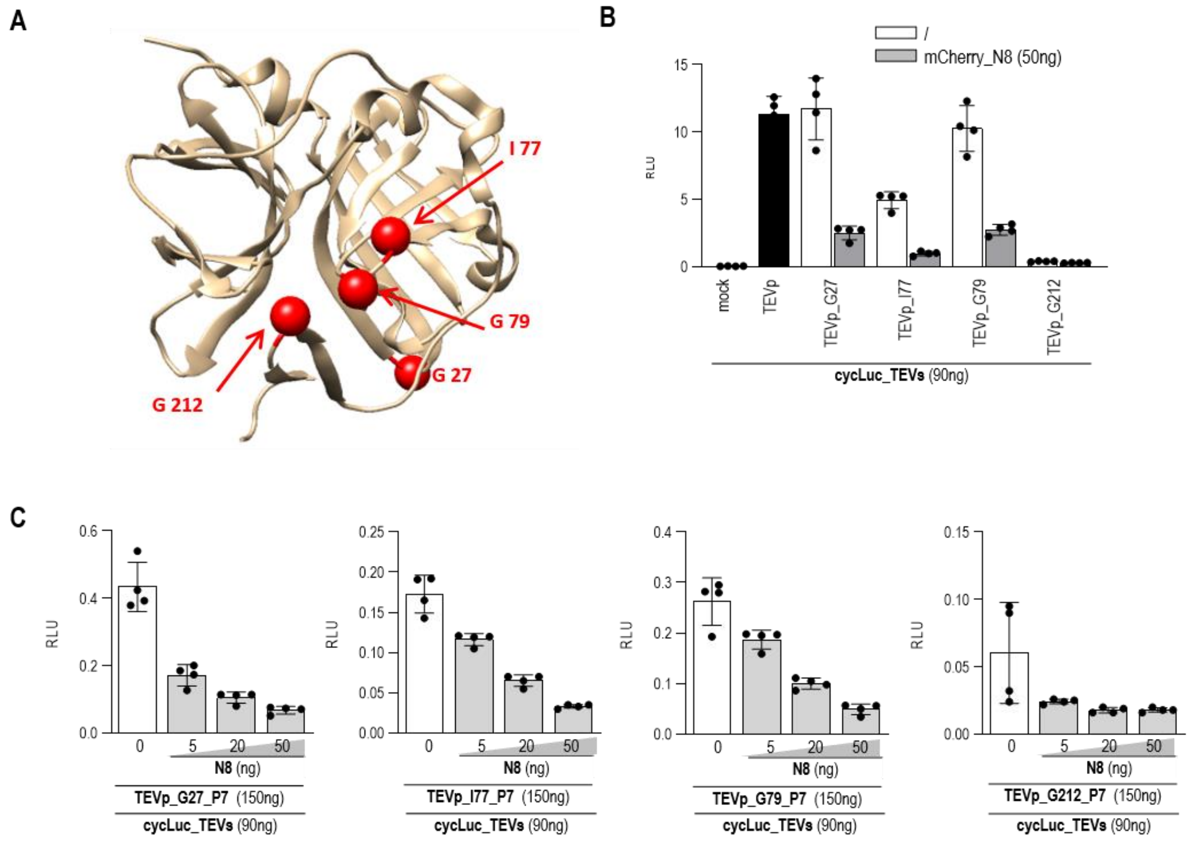

Supplementary Figure S4 -Characterization of TEV protease INSRTR variants.

A) Structure of TEVp with highlighted sites of P7 peptide (red). B) Activity of designed TEVp INSRTR versions in the absence or presence of 50 ng of plasmid encoding for mCherry_N8 C) Titration of plasmids encoding for N8 peptide on four TEVp INSRTR variants.

Values in A and C are the mean of four biological replicates ± (s.d.) and representative of three independent experiments on transiently transfected HEK293T cells.

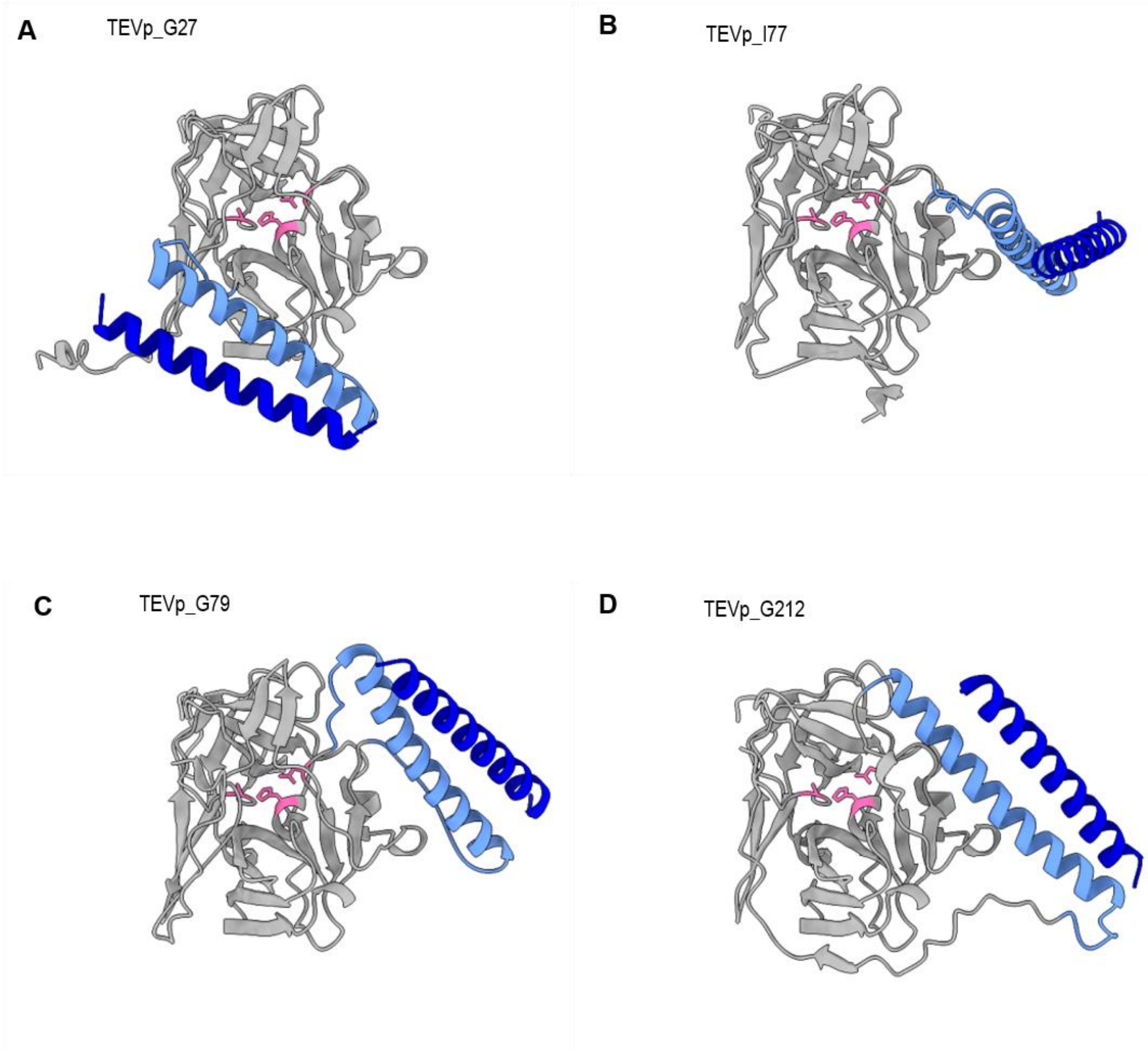

Supplementary Figure S5 – a molecular model of INSRTR TEVp variants.

TEVp with unstructured inserted peptide (INS; light blue) (the site of peptide insertion is indicated above the model) maintains structure and function, and coiled-coil formation triggered by the regulatory peptide (REG; dark blue) deactivates TEVp. The catalytic triad (His46, Asp81, and Cys151) is shown in magenta.

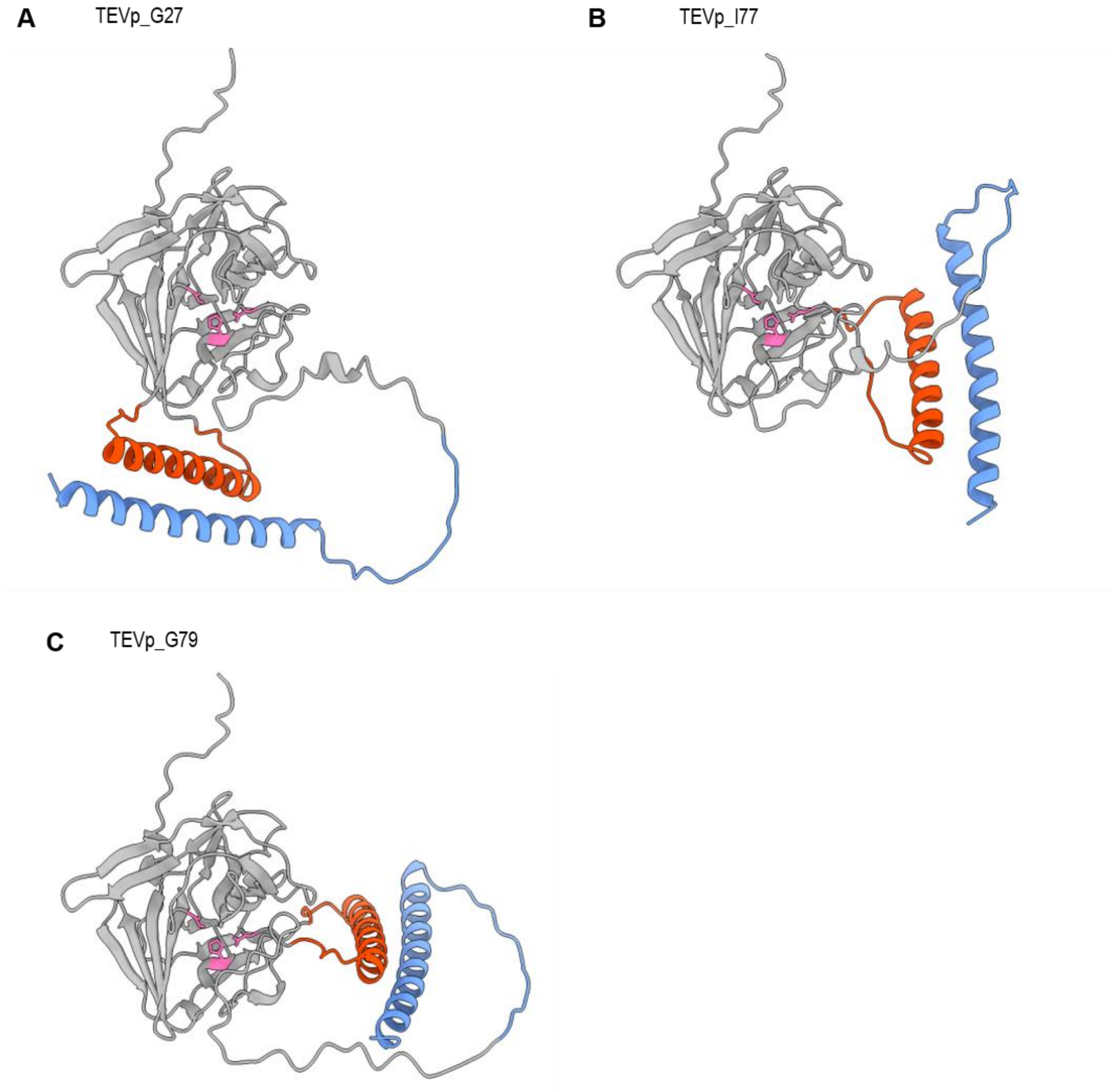

Supplementary Figure S6 – a molecular model of inverted INSRTR TEV protease.
TEV protease with unstructured inserted peptide (INS; orange) and inhibitory peptide (INH; light blue). The catalytic triad (His46, Asp81, and Cys151) is shown in magenta.

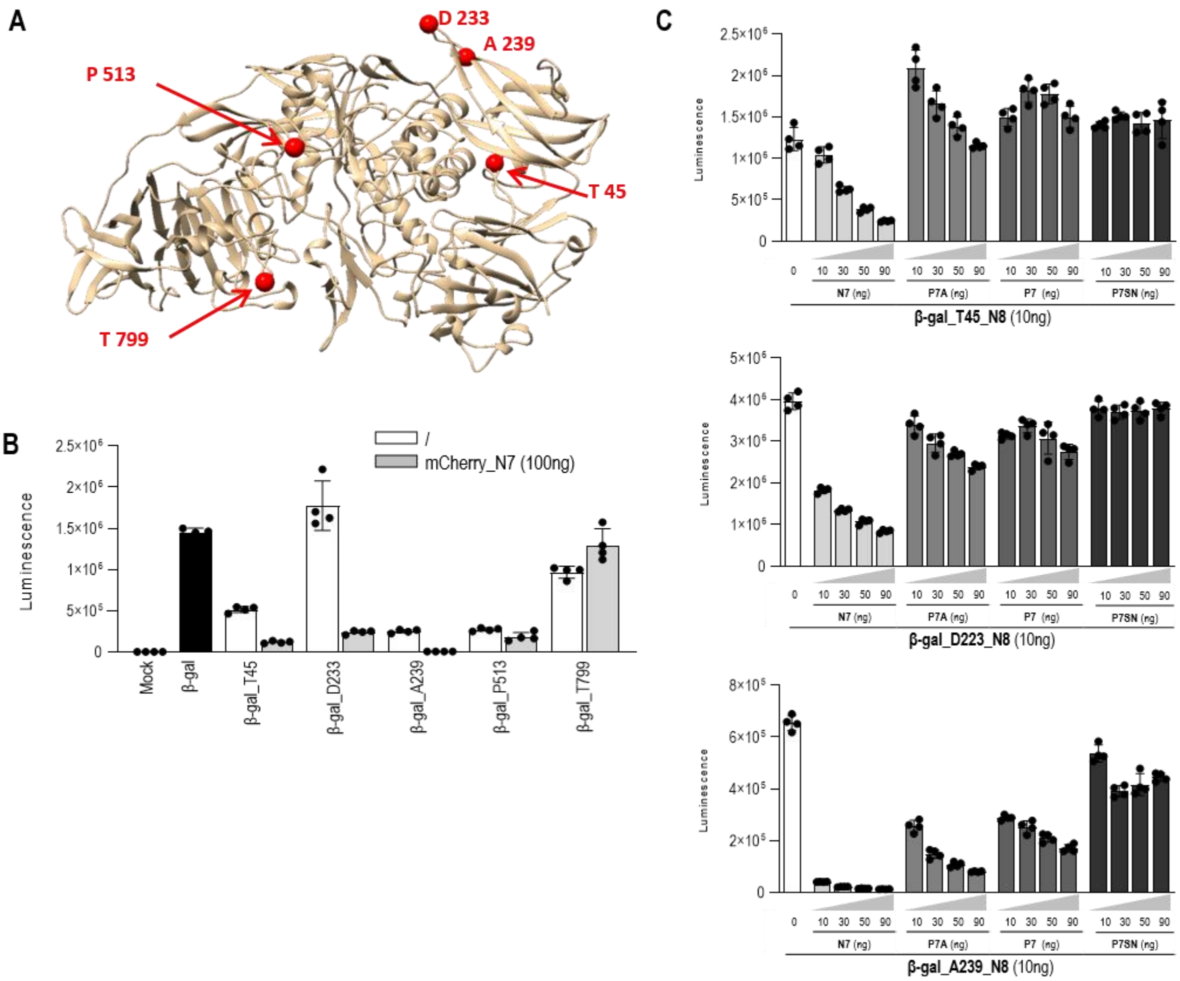

Supplementary Figure S7 – Characterization of β-galactosidase INSRTR variants.

A) Structure of β-galactosidase with highlighted sites of N8 peptide insertion (red). B) Activity of designed β-galactosidase INSRTR versions in the absence or presence of 100 ng of plasmid encoding for mCherry_N7. C) Titration of plasmids encoding different affinity variants of peptides: N7, N7A, P7, and P7SN on three β-galactosidase INSRTR variants.

Values in B and C are the mean of four biological replicates ± (s.d.) and representative of three independent experiments on transiently transfected HEK293T cells.

Values in B, C, E, F, and H are the mean of four biological replicates ± (s.d.) and representative of three independent experiments on transiently transfected HEK293T cells.

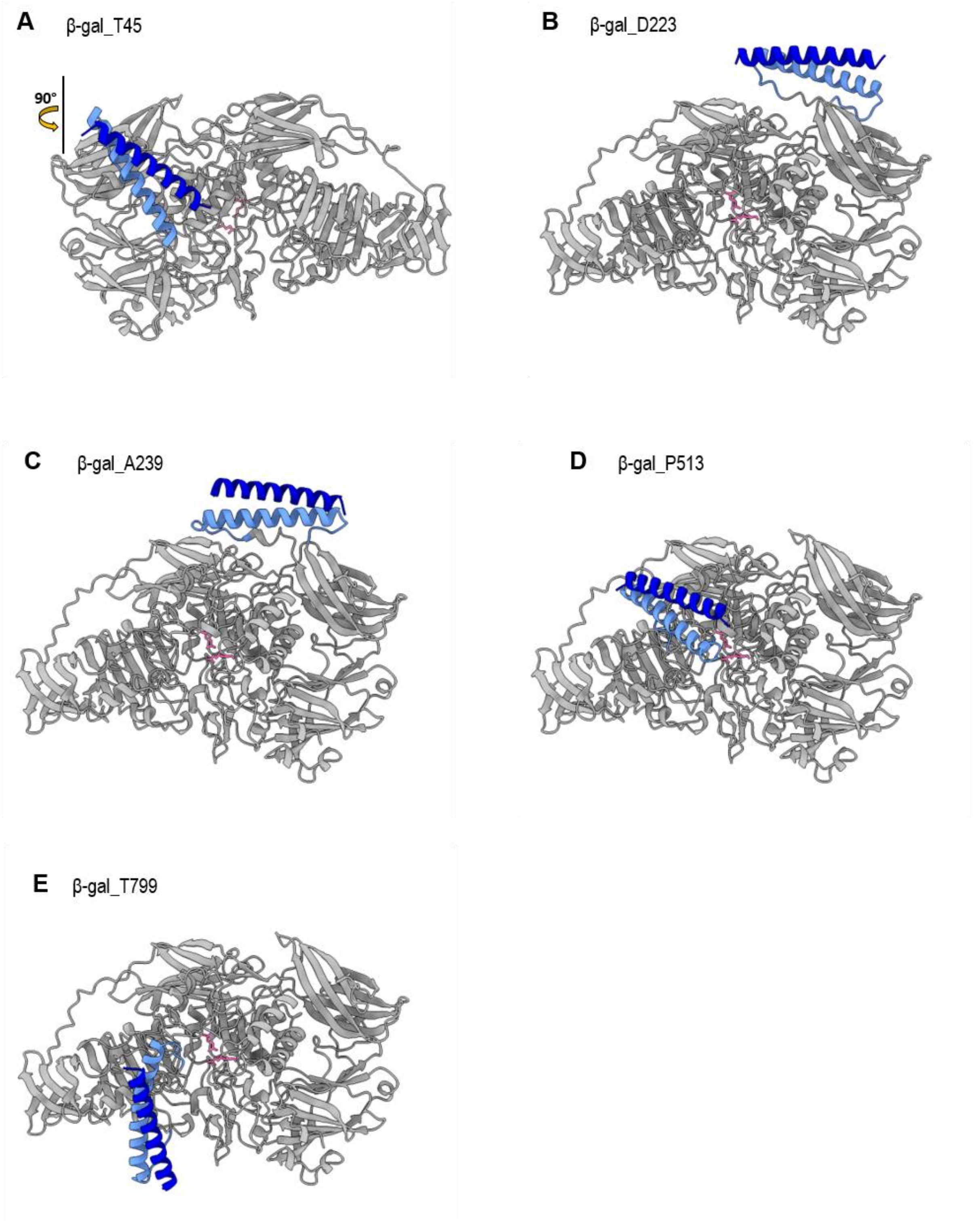

Supplementary Figure S8 – a molecular model of INSRTR β-galactosidase INSRTR variants. β-galactosidase with unstructured inserted peptide (INS; light blue) (the site of peptide insertion is indicated above the model) maintains structure and function, coiled-coil formation triggered by the regulatory peptide (REG; dark blue) deactivates β-galactosidase. The active site (Glu461 and Glu537) is shown in magenta.

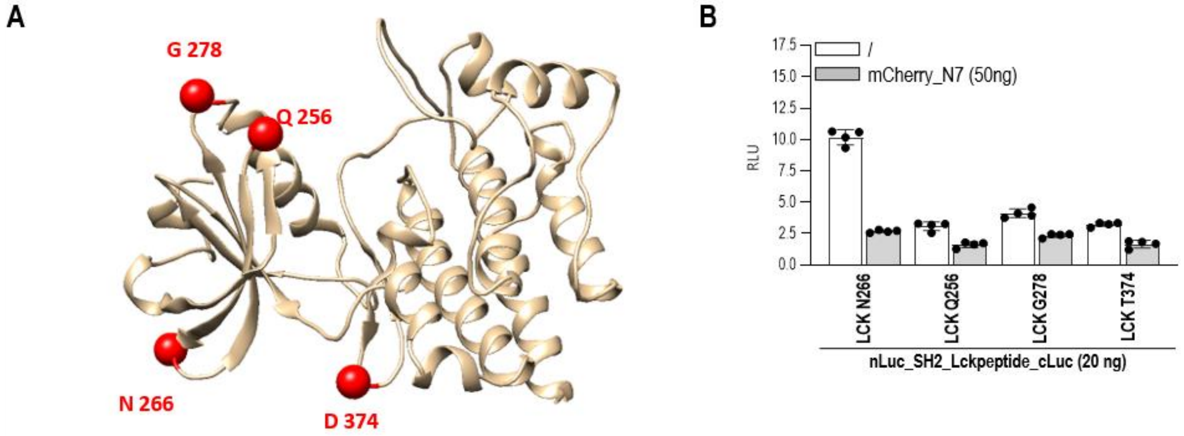

Supplementary Figure S9 – Characterization of Lck INSRTR variants.
A) Structure of Lck with highlighted sites of N8 peptide insertion (red). B) Activity of designed Lck INSRTR versions in the absence or presence of 50 ng of plasmid encoding for mCherry_N7.
Values in B are the mean of four biological replicates ± (s.d.) and representative of three independent experiments on transiently transfected HEK293T cells.

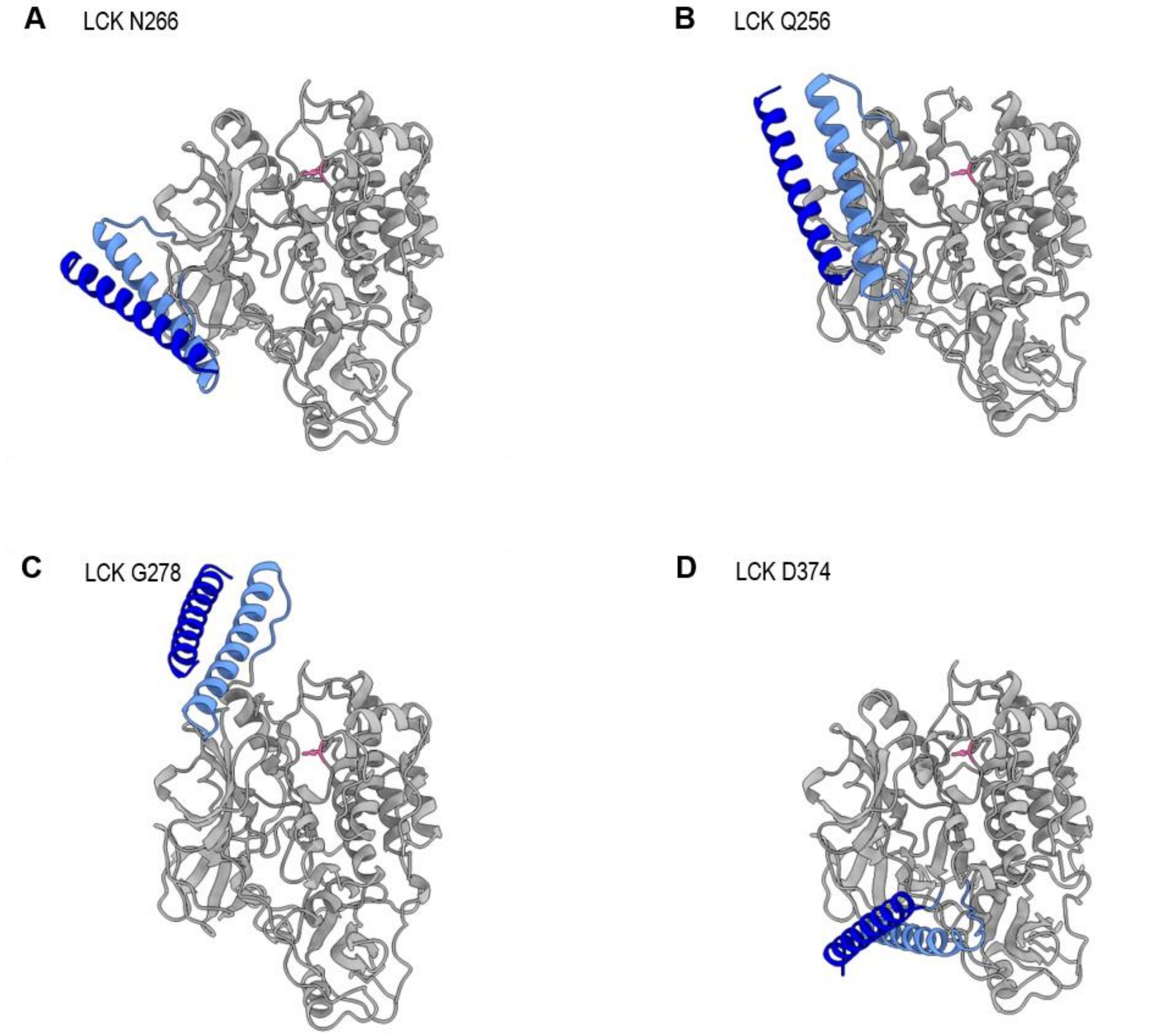

Supplementary Figure S10 – a molecular model of INSRTR Lck variants.
Lck kinase with unstructured inserted peptide (INS; light blue) (the site of peptide insertion is indicated above the model) maintains structure and function, and coiled-coil formation triggered by the regulatory peptide (REG; dark blue) deactivates Lck kinase. Active site Asp364 is shown in magenta.

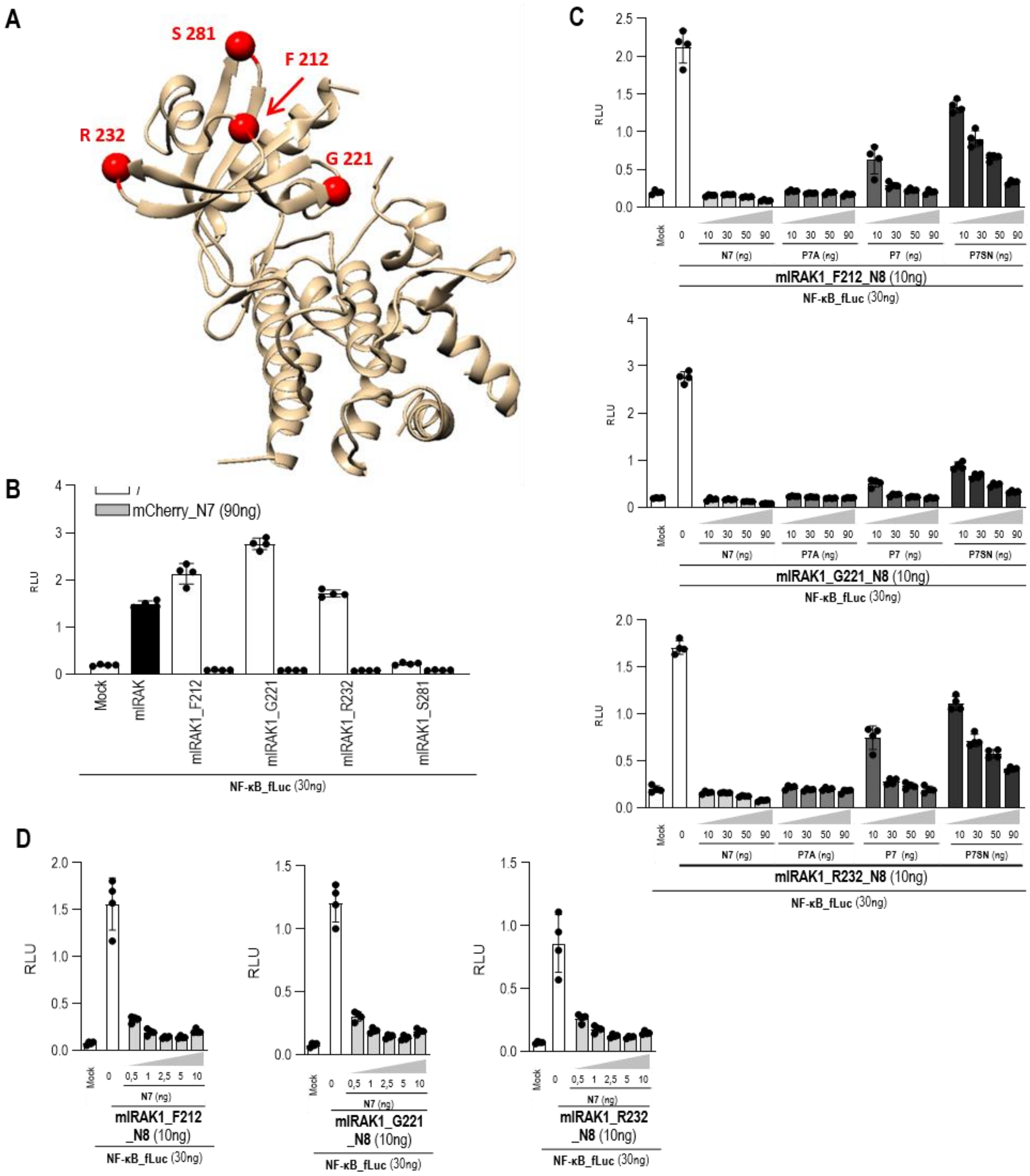

Supplementary Figure S11 - Characterization of mIRAK1 INSRTR variants.

A) Structure of hIRAK1 with highlighted sites of N8 peptide insertion (red). F212, G221, and R232 are conserved among human and mouse IRAK1. The site N281 in hIRAK1 is represented as S281 in mIRAK1. B) Activity of designed mIRAK1 INSRTR versions in the absence or presence of 90 ng of plasmid encoding for mCherry_N7. C) Titration of plasmids encoding different affinity variants of peptides: N7, N7A, P7, and P7SN on three mIRAK1 INSRTR variants. D) Titration with lower plasmid amounts encoding for N7 peptide on three mIRAK1 INSRTR variants.

Values in B, C, and D are the mean of four biological replicates ± (s.d.) and representative of three independent experiments on transiently transfected HEK293T cells.

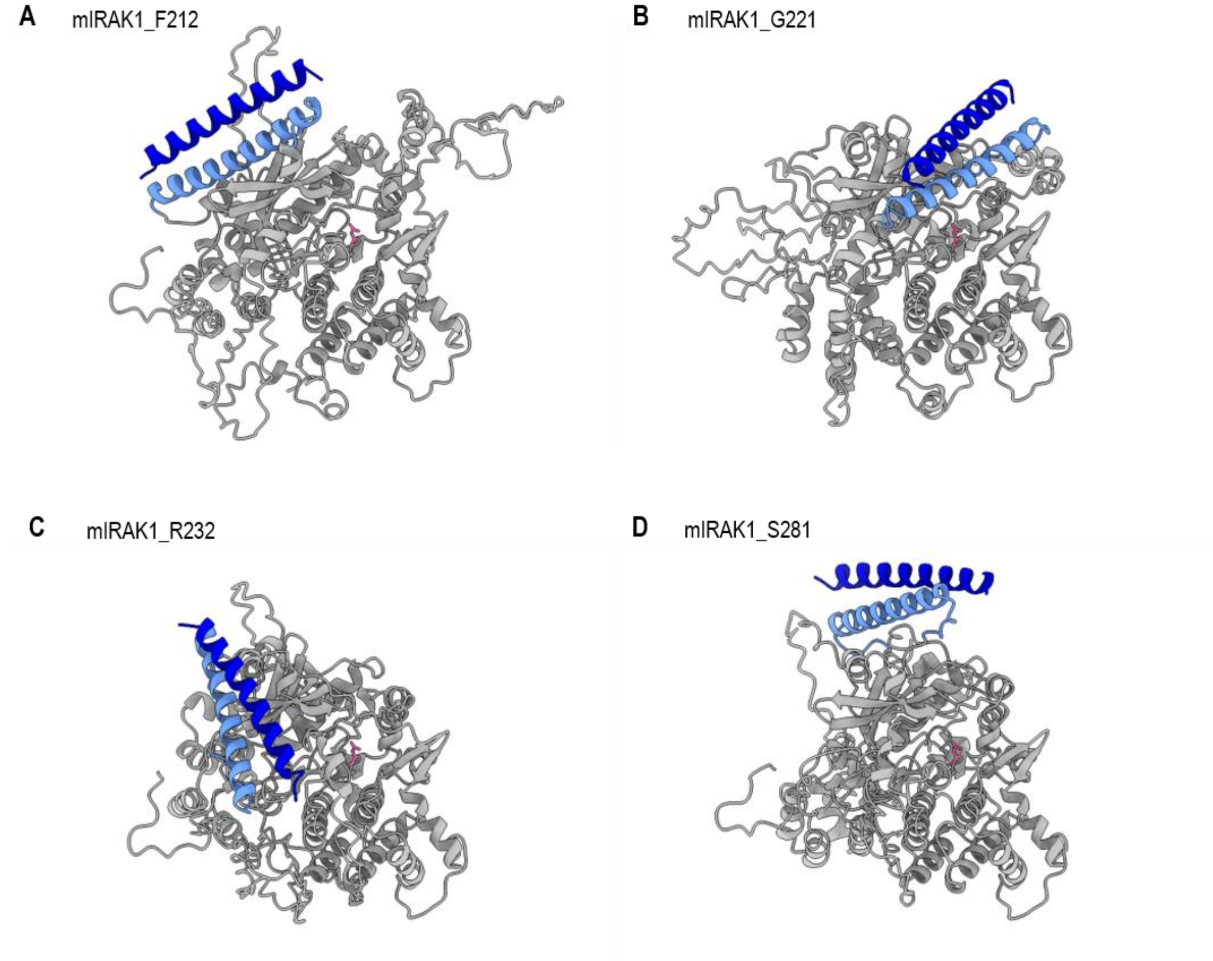

Supplementary Figure S12 – a molecular model of INSRTR mIRAK variants.
mIRAK with unstructured inserted peptide (INS; light blue) (the site of peptide insertion is indicated above the model) maintains structure and function, coiled-coil formation triggered by the regulatory peptide (REG; dark blue) deactivates mIRAK. Active site Asp340 is shown in magenta.

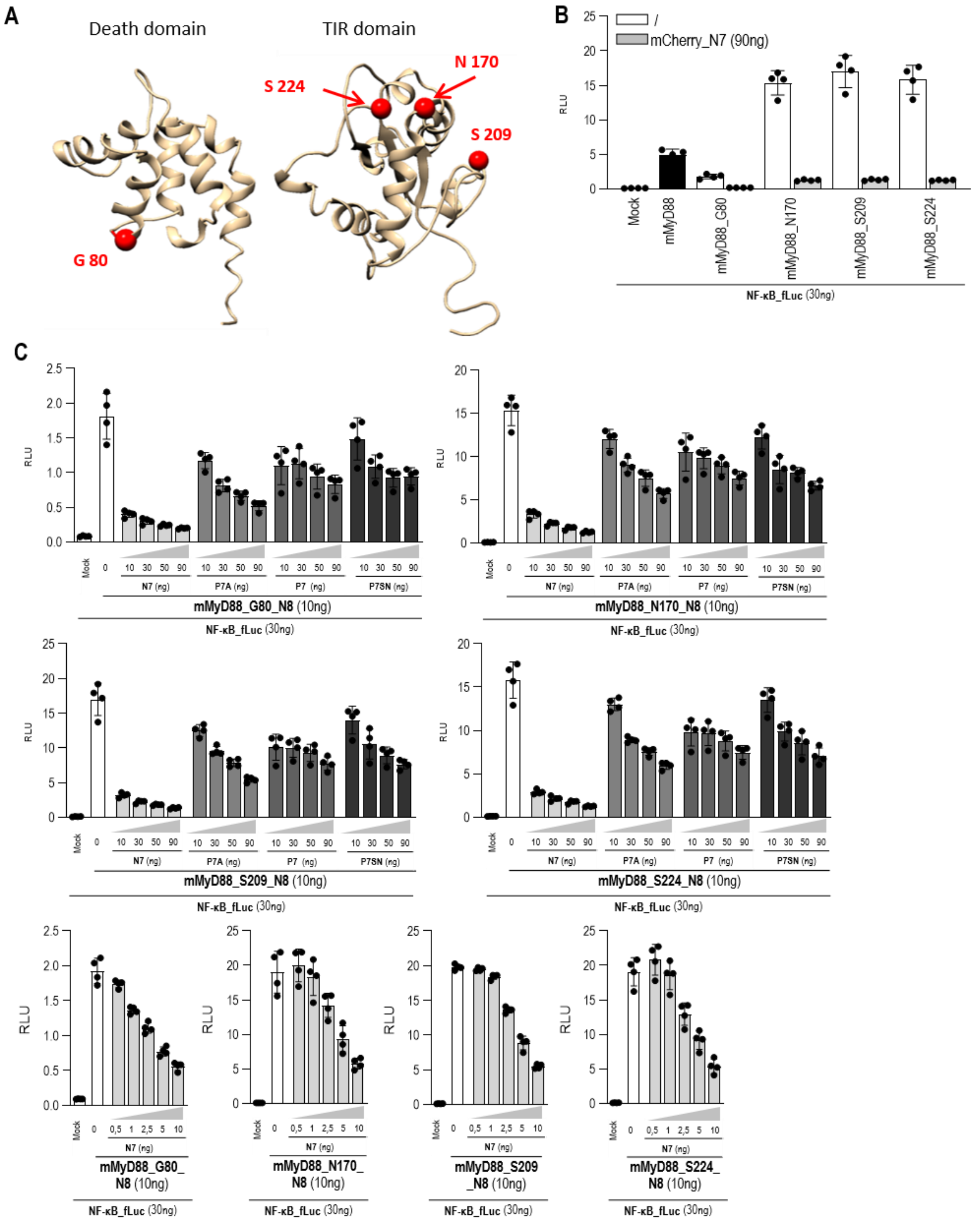

Supplementary Figure S13 – Characterization of mMyD88 INSRTR variants.
A) Structure of MyD88 death domain and TIR domain with highlighted sites of N8 peptide insertion (red). G80, S209, and S224 are conserved in human and mouse MyD88. The site S170 in hMyD88 is represented as N170 in mMyD88. B) Activity of designed mMyD88 INSRTR versions in the absence or presence of 90 ng of plasmid encoding for mCherry_N7. C) Titration of plasmids encoding different affinity variants of peptides: N7, N7A, P7, and P7SN on mMyD88 INSRTR variants. D) Titration with lower plasmid amounts encoding for N7 peptide on mMyD88 INSRTR variants.
Values in B, C, and D are the mean of four biological replicates ± (s.d.) and representative of three independent experiments on transiently transfected HEK293T cells.

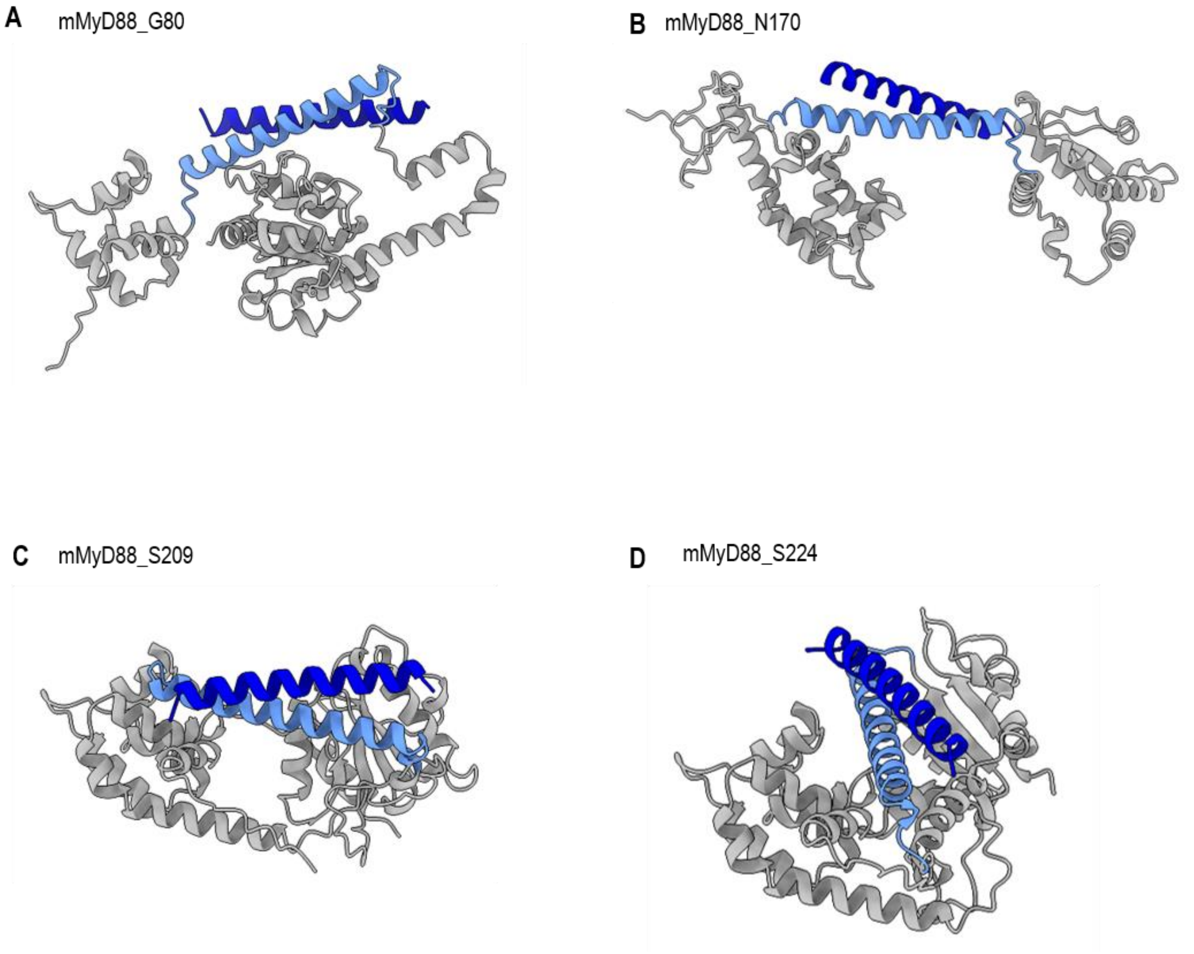

Supplementary Figure S14 – a molecular model of INSRTR MyD88 variants.

A-D) MyD88 with unstructured inserted peptide (INS; light blue) (the site of peptide insertion is indicated above the model) maintains structure and function, coiled-coil formation triggered by the regulatory peptide (REG; dark blue) deactivates MyD88.

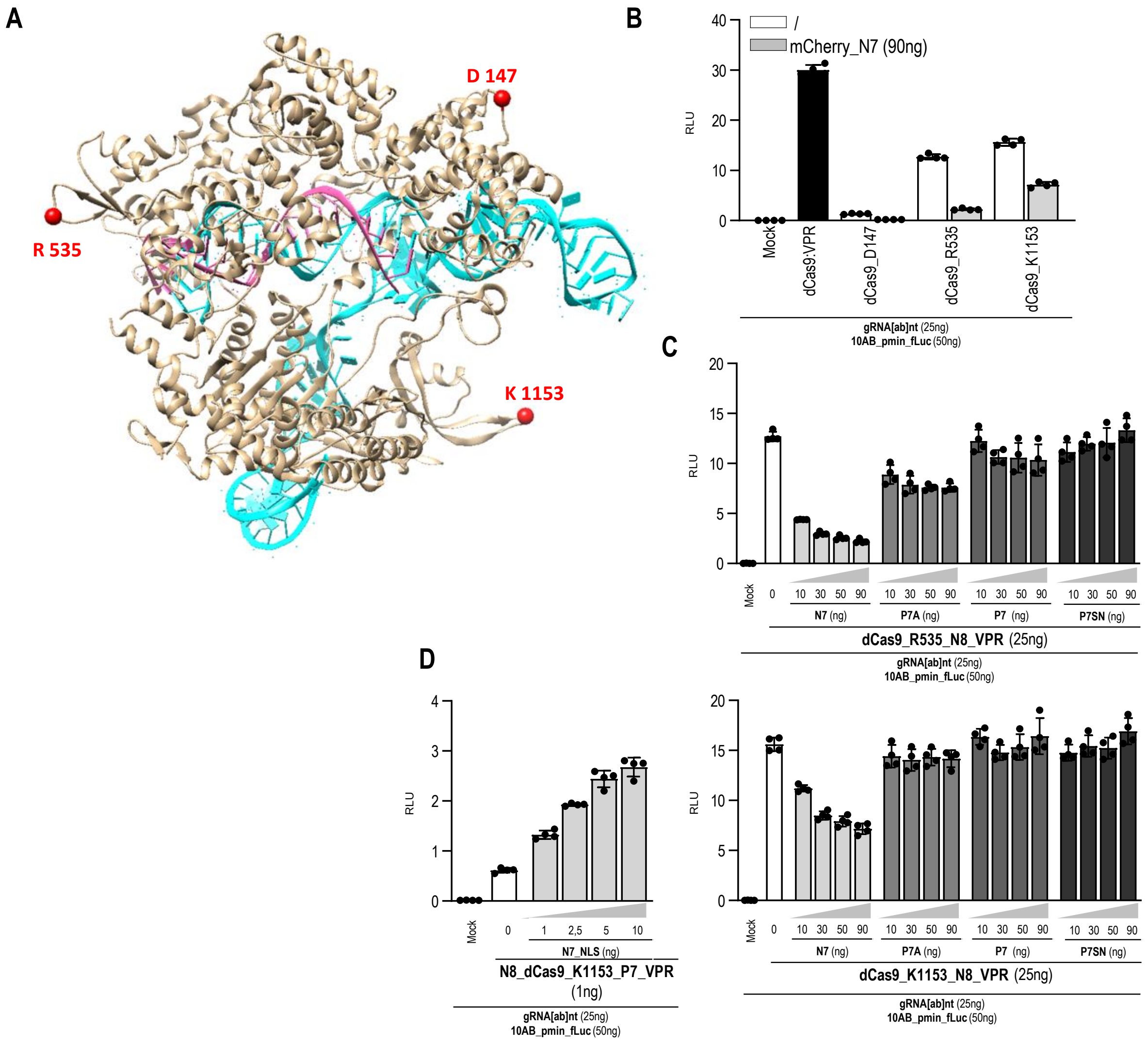

Supplementary Figure S15 - Characterization of dCas9 INSRTR variants.

A) Structure of dCas9 with highlighted sites of N8 peptide insertion (red). DNA is shown in cyan; gRNA is in magenta. B) Activity of designed dCas9 INSRTR versions in the absence or presence of 90 ng of plasmid encoding for mCherry_N7. C) Titration of plasmids encoding different affinity variants of peptides: N7, N7A, P7, and P7SN on two dCas9 INSRTR variants. D) Induction of dCas9_K1153 INSRTR with N7 peptide.

Values in B, C, and D are the mean of four biological replicates ± (s.d.) and representative of three independent experiments on transiently transfected HEK293T cells.

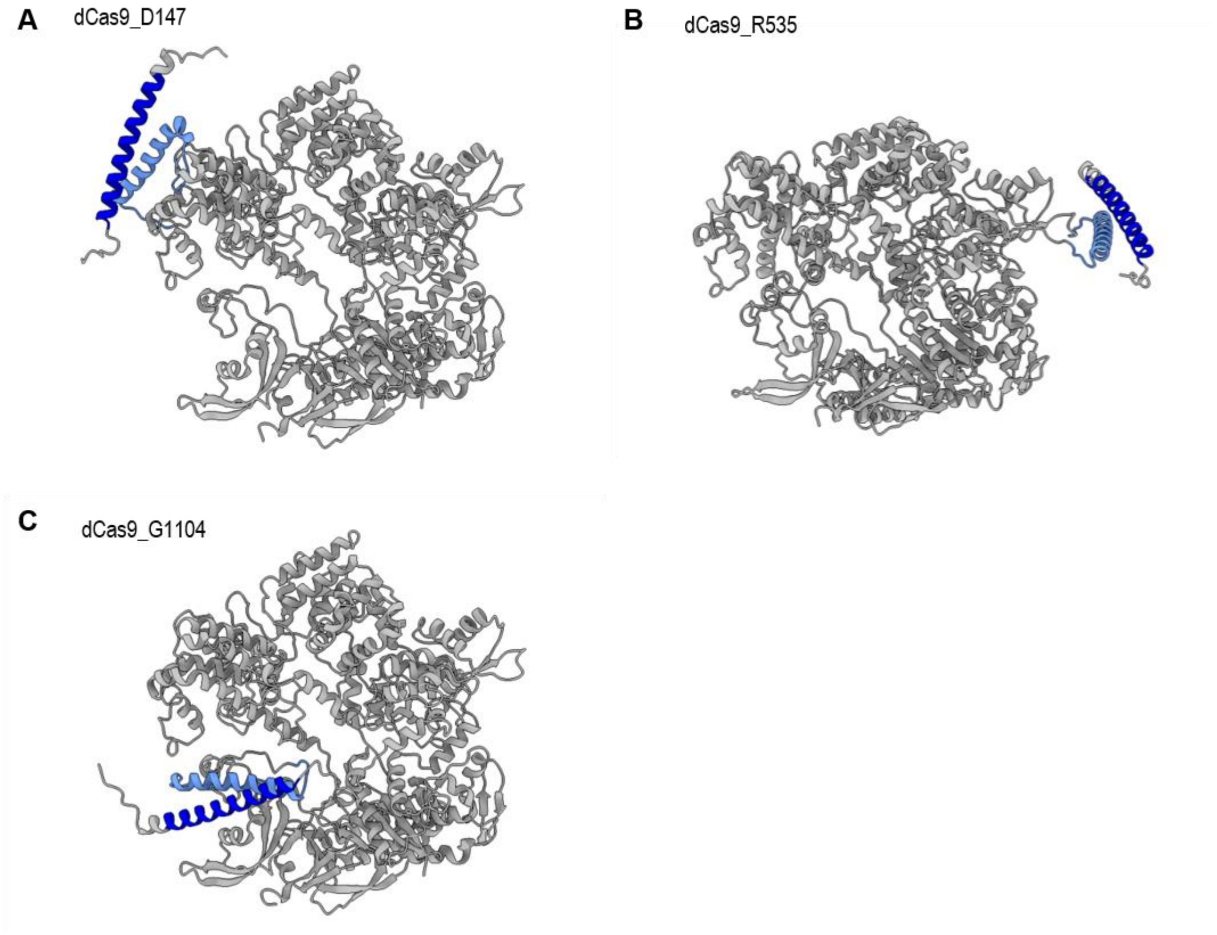

Supplementary Figure S16 – a molecular model of INSRTR dCas9 variants.
dCas9 with unstructured inserted peptide (INS; light blue) (the site of peptide insertion is indicated above the model) maintains structure and function, and coiled-coil formation triggered by the regulatory peptide (REG; dark blue) deactivates dCas9.

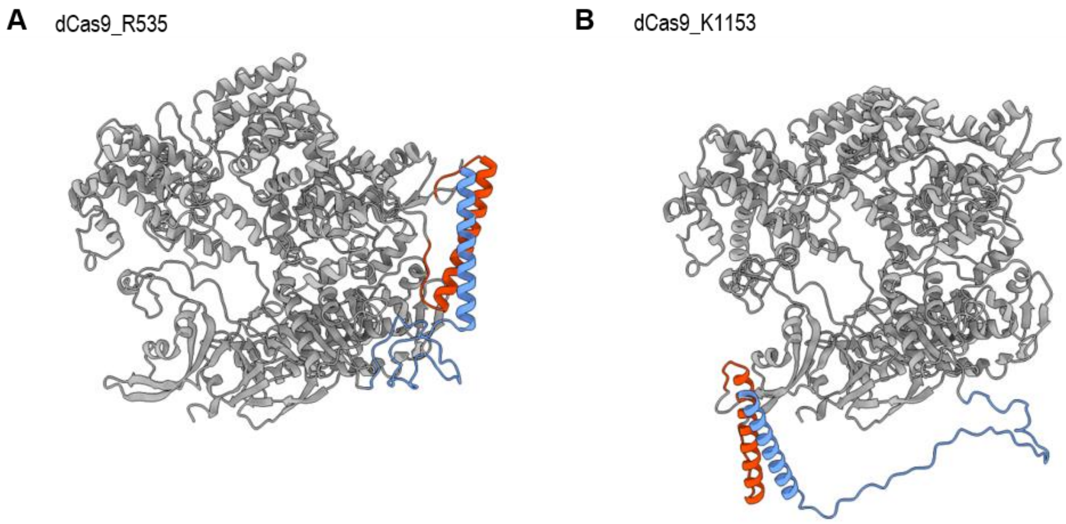

Supplementary Figure S17 – a molecular model of inverted INSRTR dCas9.
dCas9 with unstructured inserted peptide (INS; orange) (at AA position as indicated above the model) and inhibitory peptide (INH; light blue).

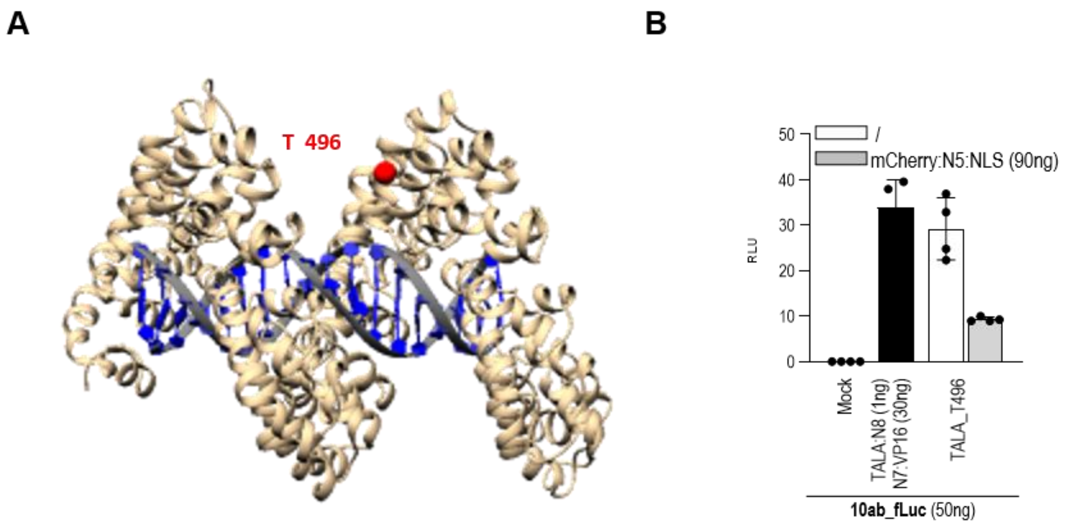

Supplementary Figure S18 – Characterization of TALE INSRTR variant.

A) Structure of TALE with highlighted site tested for N6 peptide insertion (red), DNA in blue. B) Activity of designed INSRTR variants of TALE in the absence or presence of 90 ng of plasmid encoding mCherry_N5_NLS.

Values in B are the mean of four biological replicates ± (s.d.) and representative of three independent experiments on transiently transfected HEK293T cells.

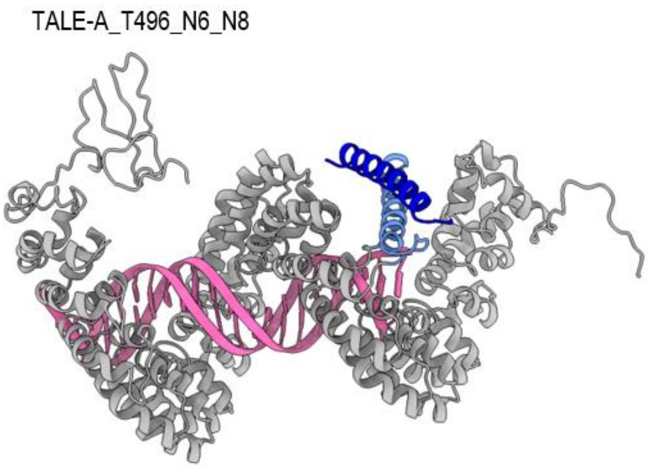

Supplementary Figure S19 – a molecular model of the INSRTR TALE variant.
TALE DNA binding domain with unstructured inserted peptide (INS; light blue) (the site of peptide insertion is indicated above the model) maintains structure and function, coiled-coil formation triggered by the regulatory peptide (REG; dark blue) deactivates TALE. N7:VP16 necessary for transcription activation was tethered to TALE via interaction with the N8 peptide at the C' terminal of TALE. DNA is shown in magenta.

N5_TALE-A_T496_N6_N8

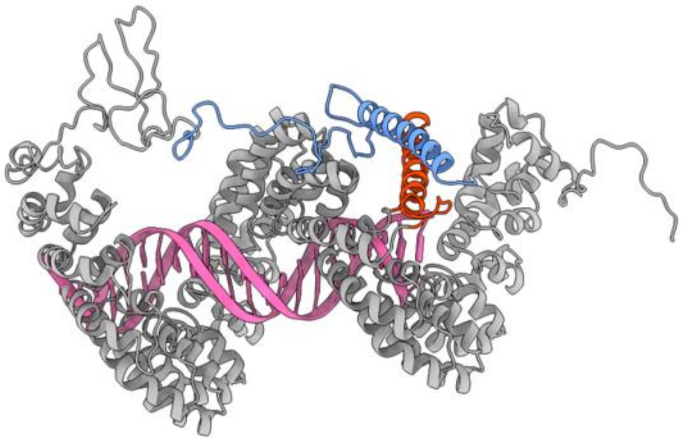

Supplementary Figure S20 – a molecular model of inverted INSRTR TALE.
TALE DNA binding domain with unstructured inserted peptide (INS; orange) and inhibitory peptide (INH; light blue). N7:VP16 necessary for transcription activation was tethered to TALE via interaction with the N8 peptide at the C' terminal of TALE. DNA is shown in magenta.

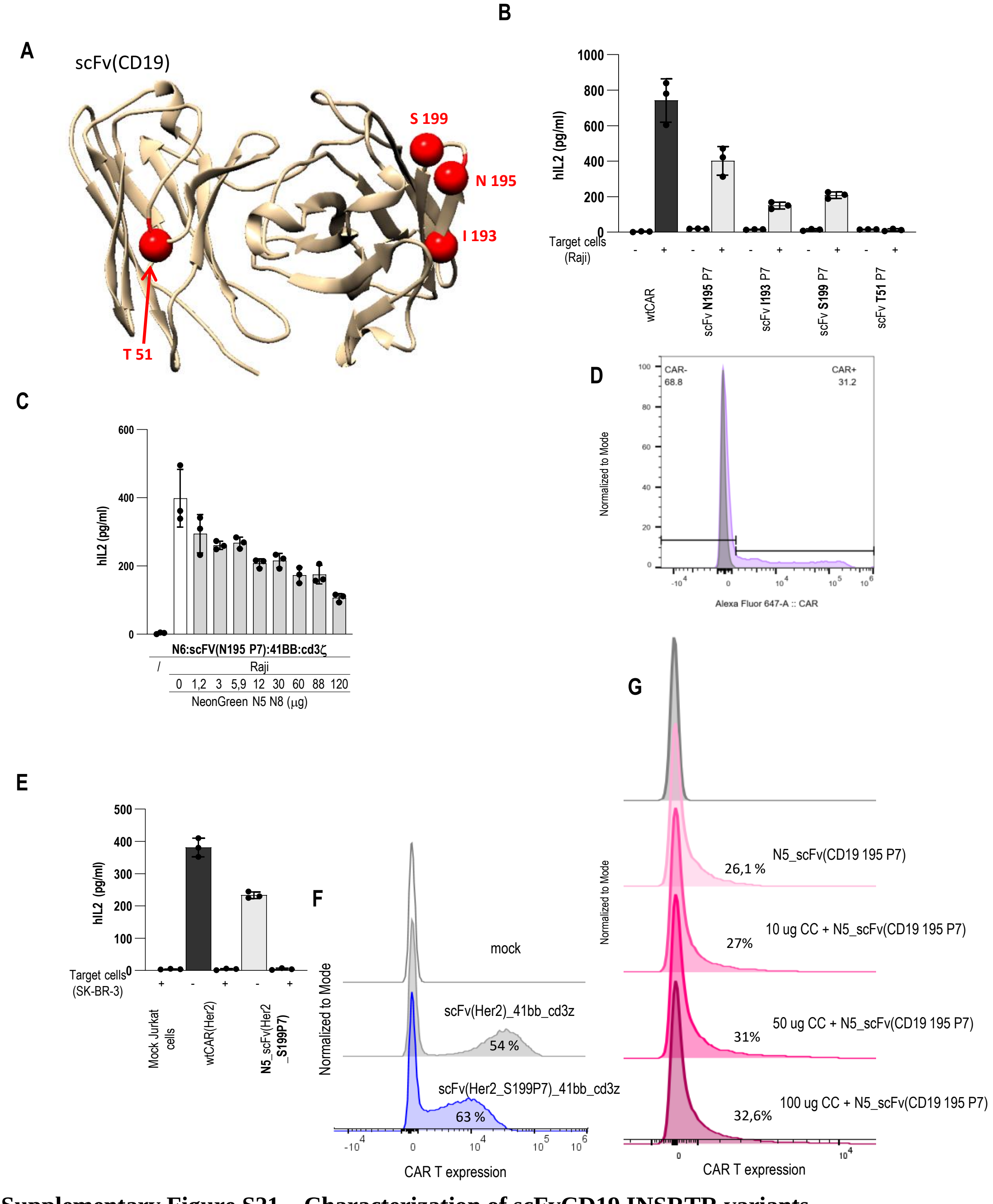

Supplementary Figure S21 – Characterization of scFvCD19 INSRTR variants.
A) Structure of scFvCD19 with highlighted sites tested for P7 peptide insertion (red). B) Activity of designed INSRTR variants of scFv CD19 CAR-T. C) Inhibition of α-CD19 CAR Jurkat T cell activation by the addition of B cells (Raji) by the addition of a coiled-coil forming peptide. Insertion into scFv at position 196 was responsive to the addition of a protein containing N8 coiled-coil forming peptide. D) Flow cytometry showing surface expression of α-CD19 INSRTR CAR-T. Cells were stained with 9B11 anti-Myc-tag AF647 antibody. E) Inhibition of α-Her2CAR Jurkat T cell activation by the addition of Her2 + SK-BR-3 cells by the addition of a coiled-coil forming peptide. Insertion of the P7 CC segment into scFv at position 199 was responsive to the addition of a protein containing N8 coiled-coil forming peptide. F) Flow cytometry showing surface expression of INSRTR α-Her2 CAR-T. Cells were stained with 9B11 anti-Myc-tag AF647 antibody. G) Flow cytometry analysis of surface

expression of CAR with or without the addition of CC peptide pair. Cells were incubated with NeonGreen_N5_P8A and afterwards stained with 9B11 anti-Myc-tag AF647 antibody.
Values in B, C and E are the mean of three biological replicates ± (s.d.) and representative of three independent experiments on transiently electroporated Jurkat cells.

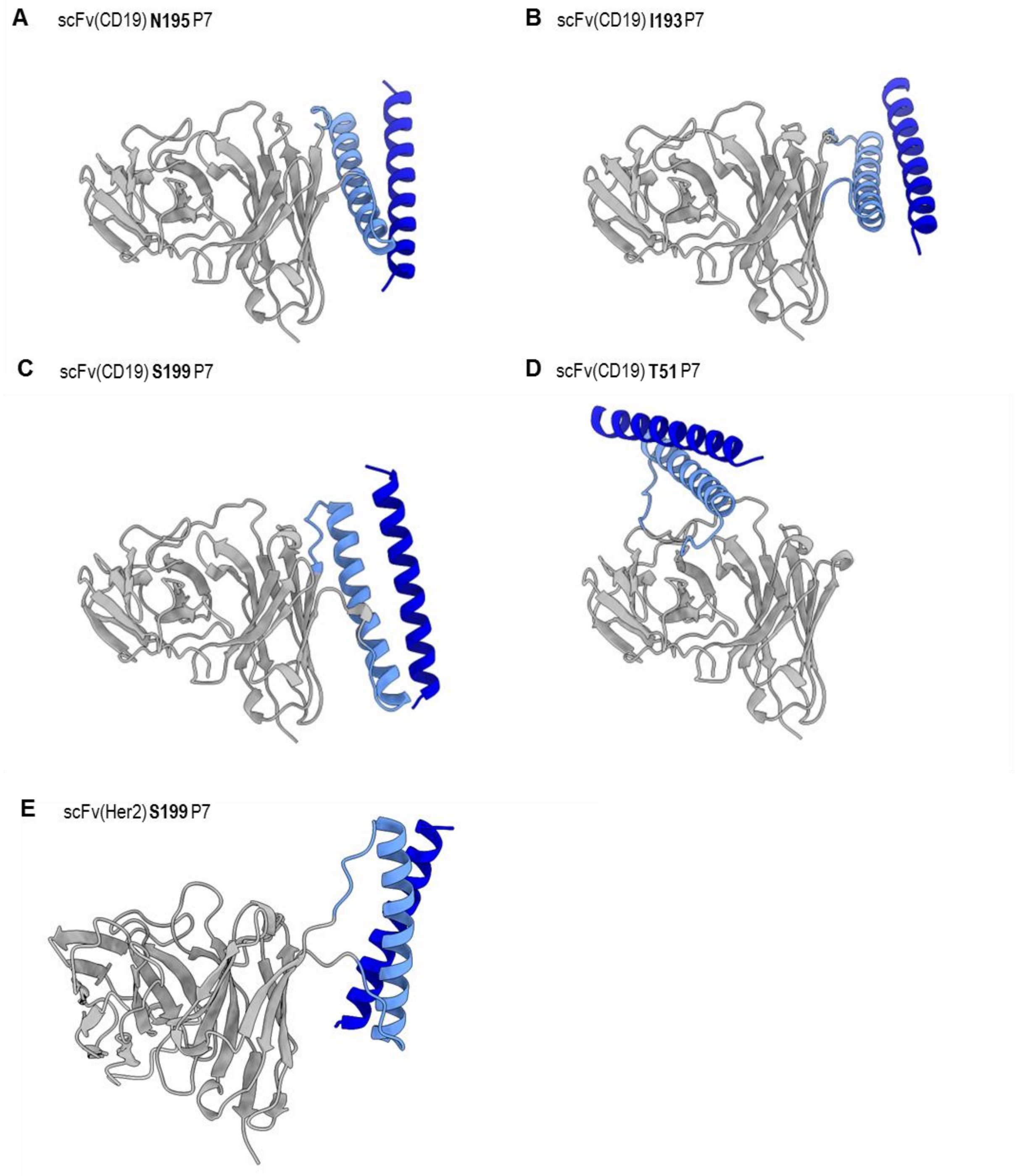

Supplementary Figure S22 – a molecular model of INSRTR scFv variants.
scFv with unstructured inserted peptide (INS; light blue) (the site of peptide insertion is indicated above the model) maintains structure and function, coiled-coil formation triggered by the regulatory peptide (REG; dark blue) deactivates scFv. A-D) scFv(CD19) INSRTR variants. E) scFv(Her2) INSRTR variant.

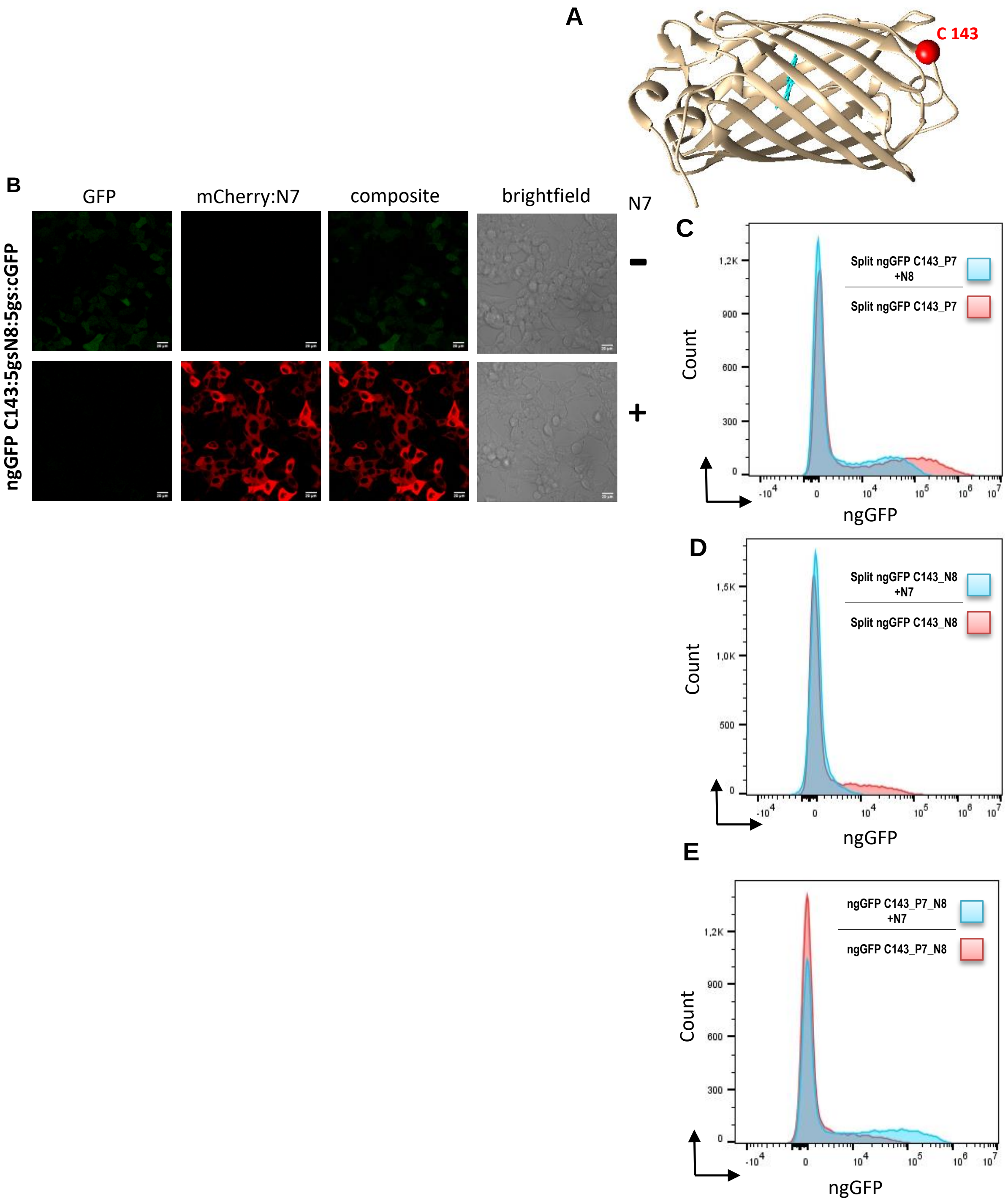

Supplementary Figure S23 - Characterization of ngGFP INSRTR variant (OFF switch) with confocal microscopy. Related to Figure 4.

A) Structure of ngGFP with the highlighted site of peptide insertion (red). B) ngGFP (50ng) with inserted peptide N8 at position C143; addition of mCherry:N7 (250 ng) deactivates ngGFP. C) Flow cytometry analysis of fluorescent protein ngGFP (400ng) with inserted peptide P7 at position C143; addition of N8 (1000ng) deactivates ngGFP. D) ngGFP (50ng) with inserted peptide N8 at position C143; addition of mCherry:N7 (250ng) deactivates ngGFP. E) ngGFP (50ng) with inserted peptide P7 and autoinhibited peptide N8 at position C143; addition of mCherry: N7 (250ng) activates ngGFP.

Note: peptide N8 has a higher helical propensity compared to P7. Insertion of N8 at position C143 thus reduces initial GFP fluorescence.

(E) Flow cytometry analysis of fluorescent protein GFP (400ng) with inserted peptide N8 at position C143; addition of N7 (1000ng) deactivates ngGFP.

A

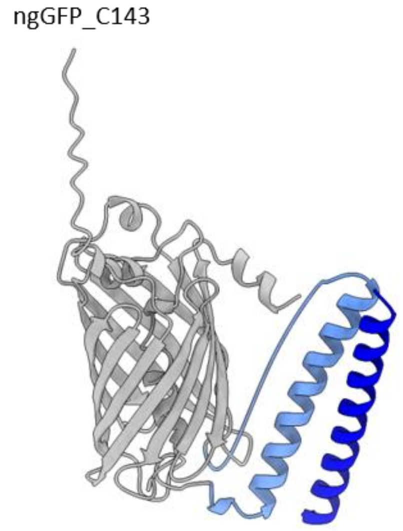

Supplementary Figure S24 – a molecular model of INSRTR GFP variants.

A) ngGFP with unstructured inserted peptide (INS; light blue) (the site of peptide insertion is indicated above the model) maintains structure and function, coiled-coil formation triggered by the regulatory peptide (REG; dark blue) deactivates GFP.

A ngGFP_C143

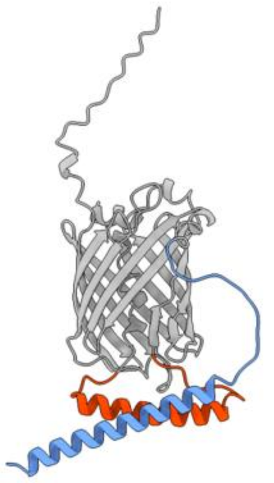

Supplementary Figure S25 – a molecular model of inverted INSRTR GFP.

ngGFP with unstructured inserted peptide (INS; orange) (the site of peptide insertion is indicated above the model) and inhibitory peptide (INH; light blue).

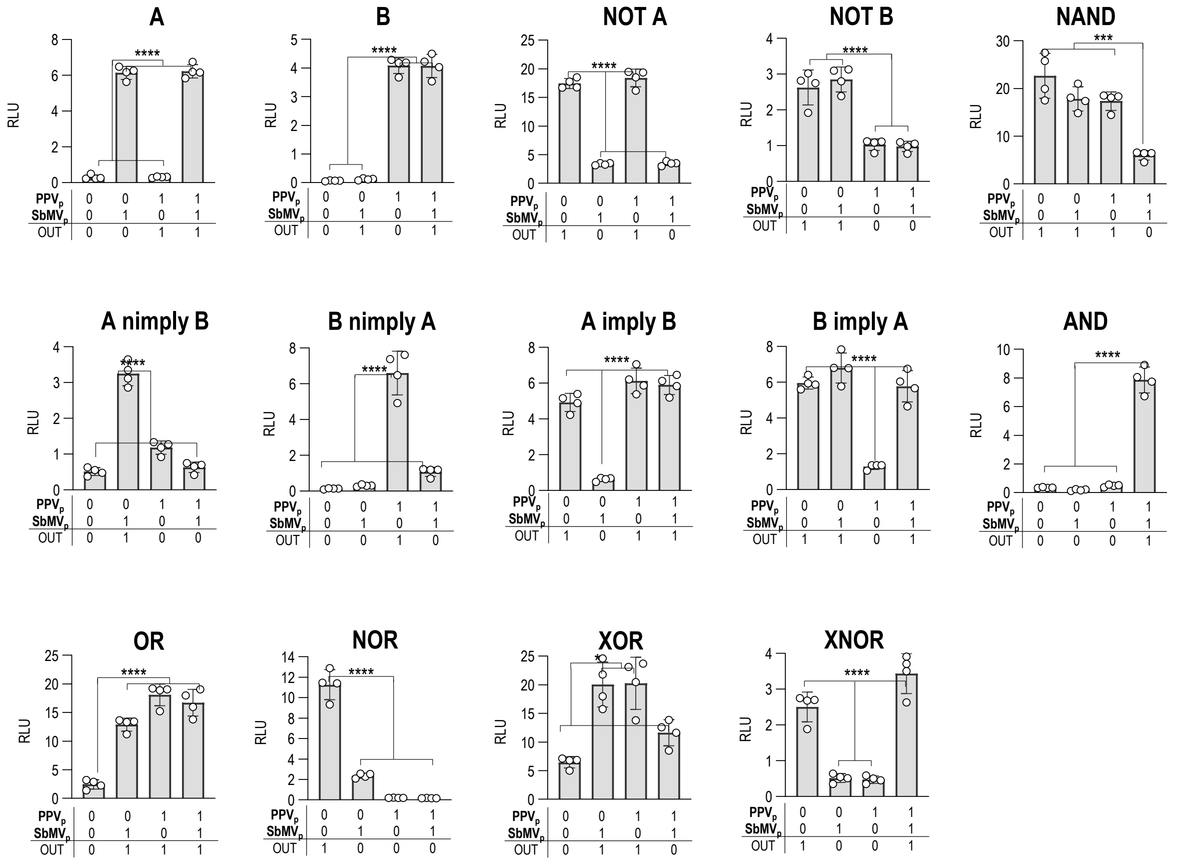

Supplementary Figure S26 - Construction of INSRTR protein logic gates based on intramolecular fusion with interacting and protease target segments. Related to Figure 3.

Non- normalized data was included for a clearer understanding of output signals.

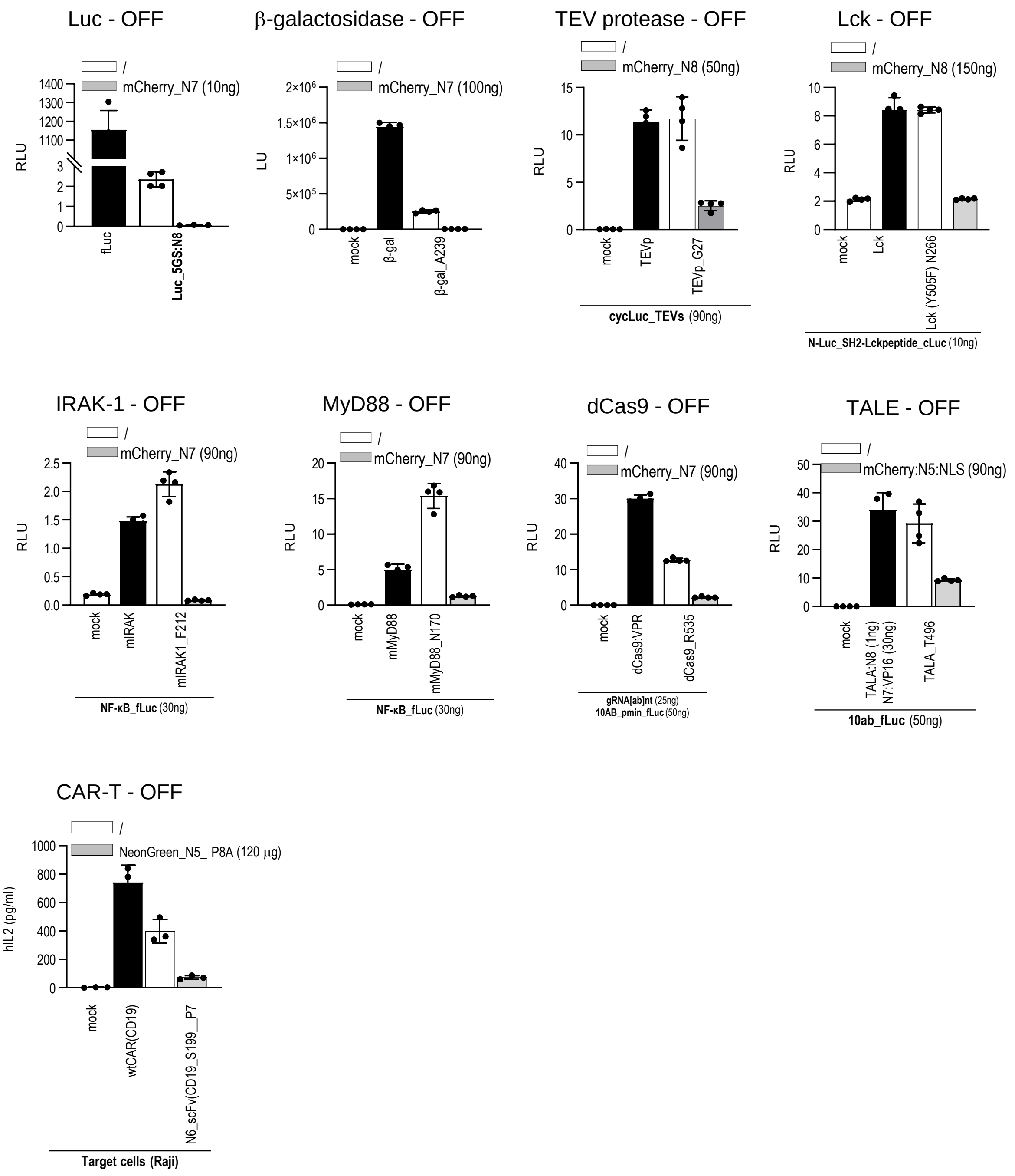

Supplementary Figure S27 - Activity comparison between INSRTR variants and native, non-engineered proteins. Related to Figure 4.

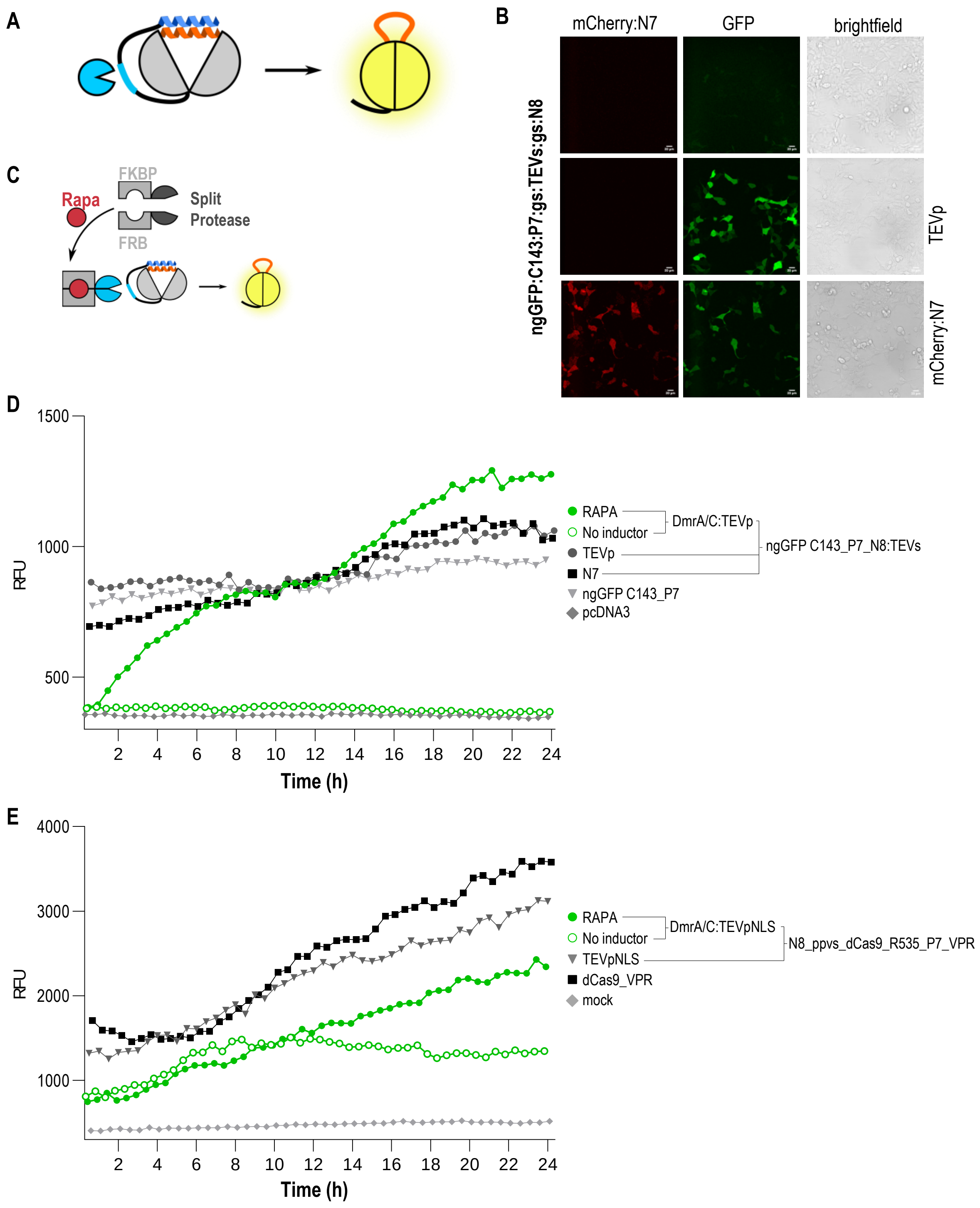

Supplementary Figure S28 - Induction of ON-INSRTR variants with rapalog.

A) Schematic representation of TEVp inducible ON-INSRTR (GFP) B) Co-expression of TEVp protease results in ON-INSRTR system activation. C) Schematic representation of r rapamycin-mediated reconstitution of split protease for induction of ON-INSRTR. D) Regulation of rapamycin-mediated reconstitution of TEVp protease results in the fast kinetics of ON-INSRTR system activation. E) Regulation of rapamycin-mediated reconstitution of PPVp protease results in the ON-INSRTR system activation. PPVp-mediated cleavage of inhibitory peptide N8 results in the activation of dCas, which enables the transcription of a fluorescent reporter protein mCitrine. Mock represents reporter plasmid encoding mCitrine and gRNA.

Experiments were made on transiently transfected HEK293T cells.

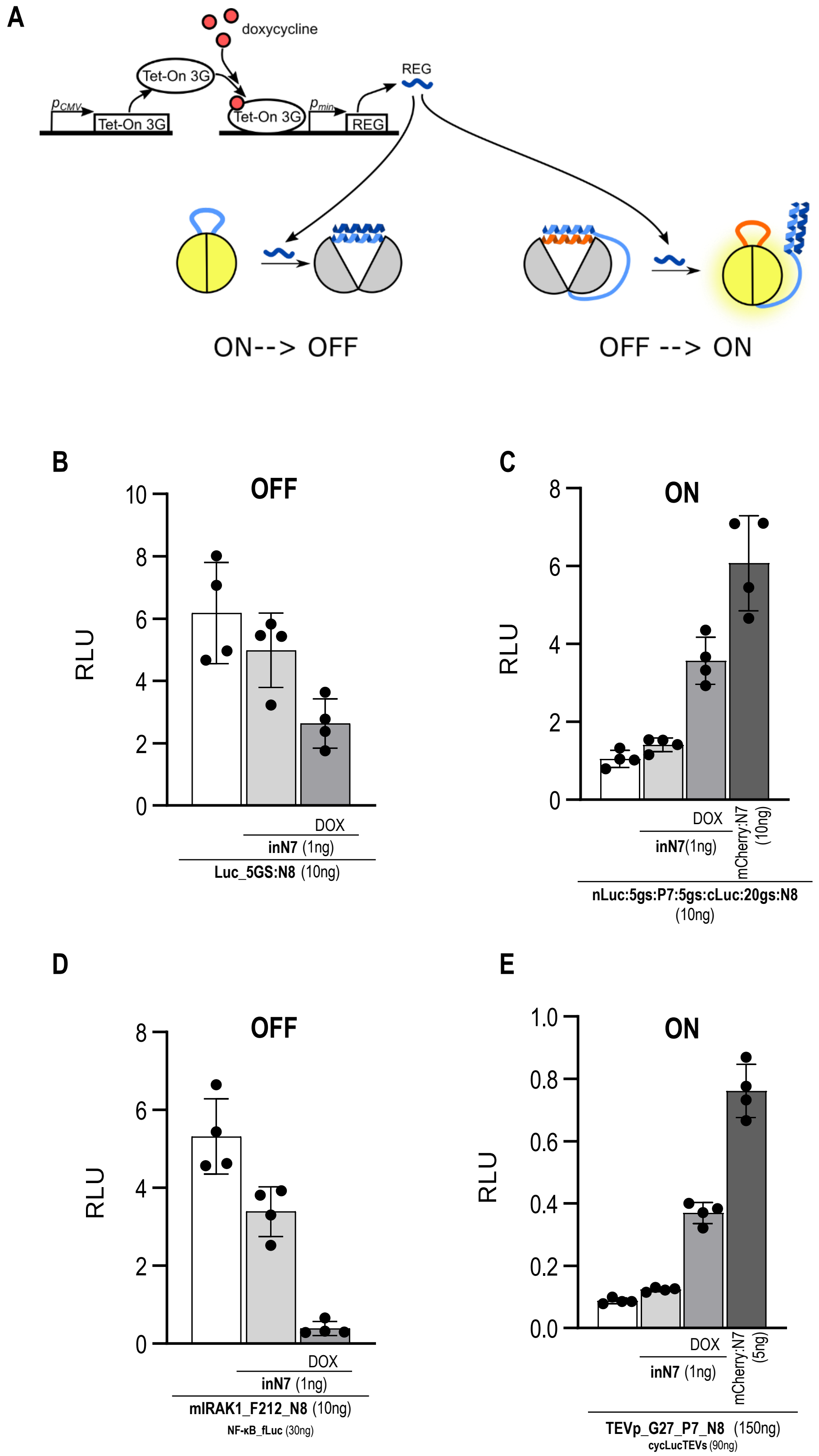

Supplementary Figure S29 - Induction of ON-INSRTR variants with chemical inductor doxycycline.

A) Schematic representation of doxycycline-inducible ON-INSRTR. Assembly is based on the Tet-On-3G system, where the addition of doxycycline leads to the activation of a transcription factor Tet-On-3G, which enables the transcription of regulatory protein REG under tet-on-3G promotor. Expression

of REG acts as an input signal that allows for inhibition of OFF-INSRTR or activation of ON-INSRTR variants. B-E) Doxycycline mediated expression of N7 (inN7) as REG presented for regulation of OFF-INSRTR B) and ON-INSRTR C) firefly luciferase, OFF-INSRTR mIRAK D) and ON-INSRTR TEVp E).

Experiments were made on transiently transfected HEK293T cells.

A

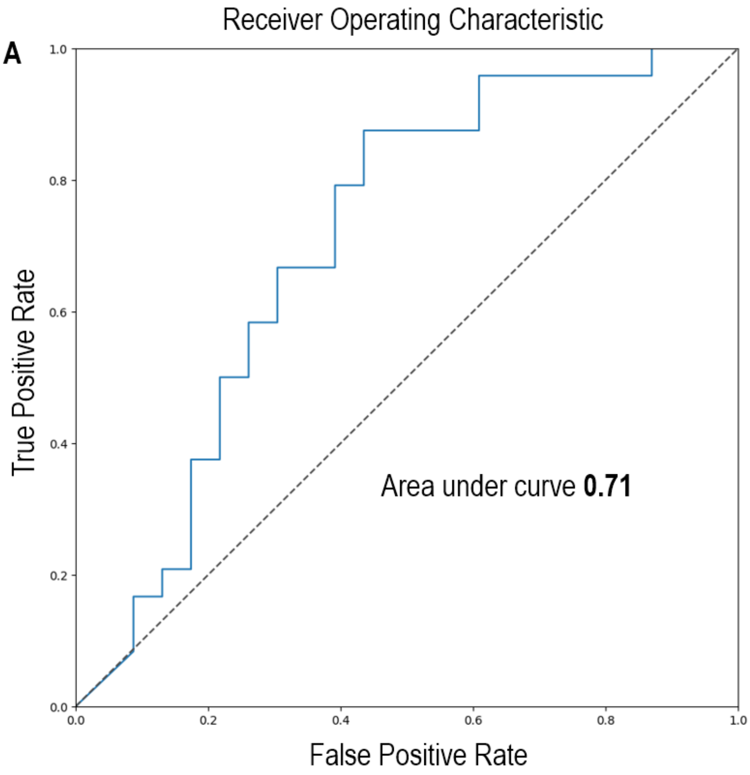

B

feature_name	importance
resi_active_site_num_E_max	0.291489
loop_sasa_A_per_res	0.172340
resi_active_site_num_H_avg	0.031915
loop_radius_gyration_A	0.031915
resi_burial_percent	0.029787
loop_seq_encoded	0.024468
loop_P_percent	0.009574
next_resi_type_encoded	0.008511
resi_loop_index0	0.000000

Supplementary Figure S30 - AUC curve for the insertion prediction model.
A) We have trained a Gradient Boosting Classifier that achieves 66% accuracy and 0.71 area under the receiver operator curve. The curve is shown in blue. The dashed yellow line shows a random classifier.
B) Most important features as identified by the permutation importance feature ranking algorithm. **resi_active_site_num_E_max --** Maximum number of extended (beta) residues between this residue and one of the active site residues, **loop_sasa_A_per_res --** Surface accessible area divided by the number of residues of the loop in A**2, **resi_active_site_num_H_avg --** Average number of helical residues between this residue and one of the active site residues, **loop_radius_gyration_A --** Radius of Gyration of the loop residues, **resi_burial_percent – (**1-SASA_residue)/SASA_residue_isolation, i.e. the percent of the residue surface covered by the rest of the protein, **loop_seq_encoded --** Aminoacid sequence of the loop in one letter code, encoded from 1 to 20, **loop_P_percent --** Percent of Proline residues in the loop, **next_resi_type – the** type of next residue encoded from 1 to 20, **resi_loop_index0 --** The zero-based index of the residue inside the loop. All calculated features are described in Supplementary File 1.

Supplementary table S1

Mutant name	Amino acid sequence
fLuc_K493_N8	MGSGEDAKNIKKGPAPFYPLEDGTAGEQLHKAMKRYALVPGTIAFTDAHIEVDITYAEYFEMSVRLAEAMKRYGLNTNHRIVVCSENSLQFFMPVLGALFIGVA<br>VAPANDIYNERELLNSMGISQPTVVFVSKKGLQKILNVQKKLPIIQKIIIMDSKTDYQGFQSMYTFVTSHLPPGFNEYDFVPESFDRDKTIALIMNSSGSTGLPKGV<br>ALPHRTACVRFSHARDPIFGNQIIPDTAILSVVPFHHGFGMFTTLGYLICGFRVVLMYRFEEELFLRSLQDYKIQSALLVPTLFSFFAKSTLIDKYDLSNLHEIASG<br>GAPLSKEVGEAVAKRFHLPGIRQGYGLTETTSAILITPEGDDKPGAVGKVVPFFEAKVVDLDTGKTLGVNQRGELCVRGPMIMSGYVNNPEATNALIDKDGWL<br>HSGDIAYWDEDEHFFIVDRLKSLIKYKGYQVAPAELESILLQHPNIFDAGVAGLPDDDAGELPAAVVVLEHGKYGKIAALKAENAALEAKIAALKAEIAALEAGTM<br>TEKEIVDYVASQVTTAKKLRGGVVFVDEVPKGLTGKLDARKIREILIKAKKGGKIAVNSGSGYPYDVPDYA*
	Firefly luciferase: black; amino acid point of insertion: red; N8 yellow; HA tag magenta
fLuc_K493_5gs:N8:5gs:	MGSGEDAKNIKKGPAPFYPLEDGTAGEQLHKAMKRYALVPGTIAFTDAHIEVDITYAEYFEMSVRLAEAMKRYGLNTNHRIVVCSENSLQFFMPVLGALFIGVA<br>VAPANDIYNERELLNSMGISQPTVVFVSKKGLQKILNVQKKLPIIQKIIIMDSKTDYQGFQSMYTFVTSHLPPGFNEYDFVPESFDRDKTIALIMNSSGSTGLPKGV<br>ALPHRTACVRFSHARDPIFGNQIIPDTAILSVVPFHHGFGMFTTLGYLICGFRVVLMYRFEEELFLRSLQDYKIQSALLVPTLFSFFAKSTLIDKYDLSNLHEIASG<br>GAPLSKEVGEAVAKRFHLPGIRQGYGLTETTSAILITPEGDDKPGAVGKVVPFFEAKVVDLDTGKTLGVNQRGELCVRGPMIMSGYVNNPEATNALIDKDGWL<br>HSGDIAYWDEDEHFFIVDRLKSLIKYKGYQVAPAELESILLQHPNIFDAGVAGLPDDDAGELPAAVVVLEHGKGSSGSYGKIAALKAENAALEAKIAALKAEIAAL<br>EAGGSGGSTMTEKEIVDYVASQVTTAKKLRGGVVFVDEVPKGLTGKLDARKIREILIKAKKGGKIAVNSGSGYPYDVPDYA*
	Firefly luciferase: black; amino acid point of insertion: red; N8 yellow; linker: gray; HA tag magenta
fLuc_K493_10gs:N8:10gs	MGSGEDAKNIKKGPAPFYPLEDGTAGEQLHKAMKRYALVPGTIAFTDAHIEVDITYAEYFEMSVRLAEAMKRYGLNTNHRIVVCSENSLQFFMPVLGALFIGVA<br>VAPANDIYNERELLNSMGISQPTVVFVSKKGLQKILNVQKKLPIIQKIIIMDSKTDYQGFQSMYTFVTSHLPPGFNEYDFVPESFDRDKTIALIMNSSGSTGLPKGV<br>ALPHRTACVRFSHARDPIFGNQIIPDTAILSVVPFHHGFGMFTTLGYLICGFRVVLMYRFEEELFLRSLQDYKIQSALLVPTLFSFFAKSTLIDKYDLSNLHEIASG<br>GAPLSKEVGEAVAKRFHLPGIRQGYGLTETTSAILITPEGDDKPGAVGKVVPFFEAKVVDLDTGKTLGVNQRGELCVRGPMIMSGYVNNPEATNALIDKDGWL<br>HSGDIAYWDEDEHFFIVDRLKSLIKYKGYQVAPAELESILLQHPNIFDAGVAGLPDDDAGELPAAVVVLEHGKGGSGGGSGGSYGKIAALKAENAALEAKIAALK<br>AEIAALEAGGGGSGGGSGGSTMTEKEIVDYVASQVTTAKKLRGGVVFVDEVPKGLTGKLDARKIREILIKAKKGGKIAVNSGSGYPYDVPDYA*
	Firefly luciferase: black; amino acid point of insertion: red; N8 yellow; linker: gray; HA tag magenta
fLuc_K493_P8A	MGSGEDAKNIKKGPAPFYPLEDGTAGEQLHKAMKRYALVPGTIAFTDAHIEVDITYAEYFEMSVRLAEAMKRYGLNTNHRIVVCSENSLQFFMPVLGALFIGVA<br>VAPANDIYNERELLNSMGISQPTVVFVSKKGLQKILNVQKKLPIIQKIIIMDSKTDYQGFQSMYTFVTSHLPPGFNEYDFVPESFDRDKTIALIMNSSGSTGLPKGV<br>ALPHRTACVRFSHARDPIFGNQIIPDTAILSVVPFHHGFGMFTTLGYLICGFRVVLMYRFEEELFLRSLQDYKIQSALLVPTLFSFFAKSTLIDKYDLSNLHEIASG<br>GAPLSKEVGEAVAKRFHLPGIRQGYGLTETTSAILITPEGDDKPGAVGKVVPFFEAKVVDLDTGKTLGVNQRGELCVRGPMIMSGYVNNPEATNALIDKDGWL<br>HSGDIAYWDEDEHFFIVDRLKSLIKYKGYQVAPAELESILLQHPNIFDAGVAGLPDDDAGELPAAVVVLEHGKYGKIAALKAENAALEAKIAALKAENAALEATMT<br>EKEIVDYVASQVTTAKKLRGGVVFVDEVPKGLTGKLDARKIREILIKAKKGGKIAVNSGSGYPYDVPDYA*
	Firefly luciferase: black; amino acid point of insertion: red; dark yellow P8A; linker: gray; HA tag magenta
fLuc_K493_P8	MGSGEDAKNIKKGPAPFYPLEDGTAGEQLHKAMKRYALVPGTIAFTDAHIEVDITYAEYFEMSVRLAEAMKRYGLNTNHRIVVCSENSLQFFMPVLGALFIGVA<br>VAPANDIYNERELLNSMGISQPTVVFVSKKGLQKILNVQKKLPIIQKIIIMDSKTDYQGFQSMYTFVTSHLPPGFNEYDFVPESFDRDKTIALIMNSSGSTGLPKGV<br>ALPHRTACVRFSHARDPIFGNQIIPDTAILSVVPFHHGFGMFTTLGYLICGFRVVLMYRFEEELFLRSLQDYKIQSALLVPTLFSFFAKSTLIDKYDLSNLHEIASG<br>GAPLSKEVGEAVAKRFHLPGIRQGYGLTETTSAILITPEGDDKPGAVGKVVPFFEAKVVDLDTGKTLGVNQRGELCVRGPMIMSGYVNNPEATNALIDKDGWL<br>HSGDIAYWDEDEHFFIVDRLKSLIKYKGYQVAPAELESILLQHPNIFDAGVAGLPDDDAGELPAAVVVLEHGKSPEDKIAQLKEENQQLEQKIQALKEENAALEY<br>TMTEKEIVDYVASQVTTAKKLRGGVVFVDEVPKGLTGKLDARKIREILIKAKKGGKIAVNSGSGYPYDVPDYA*
	Firefly luciferase: black; amino acid point of insertion: red; dark orange P8; linker: gray; HA tag magenta
fLuc_K493_10gs:P8:10gs	MGSGEDAKNIKKGPAPFYPLEDGTAGEQLHKAMKRYALVPGTIAFTDAHIEVDITYAEYFEMSVRLAEAMKRYGLNTNHRIVVCSENSLQFFMPVLGALFIGVA<br>VAPANDIYNERELLNSMGISQPTVVFVSKKGLQKILNVQKKLPIIQKIIIMDSKTDYQGFQSMYTFVTSHLPPGFNEYDFVPESFDRDKTIALIMNSSGSTGLPKGV<br>ALPHRTACVRFSHARDPIFGNQIIPDTAILSVVPFHHGFGMFTTLGYLICGFRVVLMYRFEEELFLRSLQDYKIQSALLVPTLFSFFAKSTLIDKYDLSNLHEIASG<br>GAPLSKEVGEAVAKRFHLPGIRQGYGLTETTSAILITPEGDDKPGAVGKVVPFFEAKVVDLDTGKTLGVNQRGELCVRGPMIMSGYVNNPEATNALIDKDGWL<br>HSGDIAYWDEDEHFFIVDRLKSLIKYKGYQVAPAELESILLQHPNIFDAGVAGLPDDDAGELPAAVVVLEHGKGGSGGGSGGSSPEDKIAQLKEENQQLEQKIQ<br>ALKEENAALEYGGGSGGGSGGSTMTEKEIVDYVASQVTTAKKLRGGVVFVDEVPKGLTGKLDARKIREILIKAKKGGKIAVNSGSGYPYDVPDYA*
	Firefly luciferase: black; amino acid point of insertion: red; dark orange P8; linker: gray; HA tag magenta
fLuc_K493_5gs:P7:5gs_gs:N8	MGSGEDAKNIKKGPAPFYPLEDGTAGEQLHKAMKRYALVPGTIAFTDAHIEVDITYAEYFEMSVRLAEAMKRYGLNTNHRIVVCSENSLQFFMPVLGALFIGVA<br>VAPANDIYNERELLNSMGISQPTVVFVSKKGLQKILNVQKKLPIIQKIIIMDSKTDYQGFQSMYTFVTSHLPPGFNEYDFVPESFDRDKTIALIMNSSGSTGLPKGV<br>ALPHRTACVRFSHARDPIFGNQIIPDTAILSVVPFHHGFGMFTTLGYLICGFRVVLMYRFEEELFLRSLQDYKIQSALLVPTLFSFFAKSTLIDKYDLSNLHEIASG<br>GAPLSKEVGEAVAKRFHLPGIRQGYGLTETTSAILITPEGDDKPGAVGKVVPFFEAKVVDLDTGKTLGVNQRGELCVRGPMIMSGYVNNPEATNALIDKDGWL<br>HSGDIAYWDEDEHFFIVDRLKSLIKYKGYQVAPAELESILLQHPNIFDAGVAGLPDDDAGELPAAVVVLEHGKGSSGSEIQALEEKNAQLKQEIAALEEKNQALK<br>YGGSGGSTMTEKEIVDYVASQVTTAKKLRGGVVFVDEVPKGLTGKLDARKIREILIKAKKGGKIAVNSGSGYPYDVPDYAGSPGGSPGGSGSPGGSPGGSKIA<br>ALKAENAALEAKIAALKAEIAALEAGY*
	Firefly luciferase: black; amino acid point of insertion: red; P7: green; linker: gray; N8 yellow ; HA tag magenta
TEVp_G27_5gs:P7:5gs	MGESLFKGPRDYNPISSTICHLTNESDGGSSGSEIQALEEKNAQLKQEIAALEEKNQALKYGGSGGSHTTSLYGIGFGPFIITNKHLFRRNNGTLLVQSLHGVFK<br>VKNTTTLQQHLIDGRDMIIIRMPKDFPPFPQKLKFREPQREERICLVTTNFQTKSMSSMVSDTSCTFPSSDGIFWKHWIQTKDGQCGSPLVSTRDGFIVGIHSAS<br>NFTNTNNYFTSVPKNFMELLTNQEAQQWVSGWRLNADSVLWGGHKVFMSKPEEPFQPVKEATQLMNEGGGLE*
	TEV protease: black; amino acid point of insertion: red; P7: green; linker: gray
TEVp_L72_5gs:P7:5gs	MGESLFKGPRDYNPISSTICHLTNESDGHTTSLYGIGFGPFIITNKHLFRRNNGTLLVQSLHGVFKVKNTTTLGSSGSEIQALEEKNAQLKQEIAALEEKNQALKY<br>GGSGGSQQHLIDGRDMIIIRMPKDFPPFPQKLKFREPQREERICLVTTNFQTKSMSSMVSDTSCTFPSSDGIFWKHWIQTKDGQCGSPLVSTRDGFIVGIHSAS<br>NFTNTNNYFTSVPKNFMELLTNQEAQQWVSGWRLNADSVLWGGHKVFMSKPEEPFQPVKEATQLMNEGGGLE*
	TEV protease: black; amino acid point of insertion: red; P7: green; linker: gray
TEVp_I77_5gs:P7:5gs	MGESLFKGPRDYNPISSTICHLTNESDGHTTSLYGIGFGPFIITNKHLFRRNNGTLLVQSLHGVFKVKNTTTLQQHLIGSSGSEIQALEEKNAQLKQEIAALEEKN<br>QALKYGGSGGSDGRDMIIIRMPKDFPPFPQKLKFREPQREERICLVTTNFQTKSMSSMVSDTSCTFPSSDGIFWKHWIQTKDGQCGSPLVSTRDGFIVGIHSA<br>SNFTNTNNYFTSVPKNFMELLTNQEAQQWVSGWRLNADSVLWGGHKVFMSKPEEPFQPVKEATQLMNEGGGLE*
	TEV protease: black; amino acid point of insertion: red; P7: green; linker: gray
TEVp_G79_5gs:P7:5gs	MGESLFKGPRDYNPISSTICHLTNESDGHTTSLYGIGFGPFIITNKHLFRRNNGTLLVQSLHGVFKVKNTTTLQQHLIDGGSSGSEIQALEEKNAQLKQEIAALEE<br>KNQALKYGGSGGSRDMIIIRMPKDFPPFPQKLKFREPQREERICLVTTNFQTKSMSSMVSDTSCTFPSSDGIFWKHWIQTKDGQCGSPLVSTRDGFIVGIHSA<br>SNFTNTNNYFTSVPKNFMELLTNQEAQQWVSGWRLNADSVLWGGHKVFMSKPEEPFQPVKEATQLMNEGGGLE*
	TEV protease: black; amino acid point of insertion: red; P7: green; linker: gray

TEVp_K147_5gs:P7:5gs	MGESLFKGPRDYNPISSTICHLTNESDGHTTSLYGIGFGPFIITNKHLFRRNNGTLLVQSLHGVFKVKNTTTLQQHLIDGRDMIIIRMPKDFPPFPQKLKFREPQREERICLVTTNFQTKSMSSMVSDTSCTFPSSDGIFWKHWIQTKGSSGSEIQALEEKNAQLKQEIAALEEKNQALKYGGSGGSDGQCGSPLVSTRDGFIVGIHSASNFTNTNNYFTSVPKNFMELLTNQEAQQWVSGWRLNADSVLWGGHKVFMSKPEEPFQPVKEATQLMNEGGGLE*
	TEV protease: black; amino acid point of insertion: red; P7: green; linker: gray
TEVp_F172_5gs:P7:5gs	MGESLFKGPRDYNPISSTICHLTNESDGHTTSLYGIGFGPFIITNKHLFRRNNGTLLVQSLHGVFKVKNTTTLQQHLIDGRDMIIIRMPKDFPPFPQKLKFREPQREERICLVTTNFQTKSMSSMVSDTSCTFPSSDGIFWKHWIQTKDGQCGSPLVSTRDGFIVGIHSASNFGSSGSEIQALEEKNAQLKQEIAALEEKNQALKYGGSGGSTNTNNYFTSVPKNFMELLTNQEAQQWVSGWRLNADSVLWGGHKVFMSKPEEPFQPVKEATQLMNEGGGLE*
	TEV protease: black; amino acid point of insertion: red; P7: green; linker: gray
TEVp_T175_5gs:P7:5gs	MGESLFKGPRDYNPISSTICHLTNESDGHTTSLYGIGFGPFIITNKHLFRRNNGTLLVQSLHGVFKVKNTTTLQQHLIDGRDMIIIRMPKDFPPFPQKLKFREPQREERICLVTTNFQTKSMSSMVSDTSCTFPSSDGIFWKHWIQTKDGQCGSPLVSTRDGFIVGIHSASNFTNTGSSGSEIQALEEKNAQLKQEIAALEEKNQALKYGGSGGSNNYFTSVPKNFMELLTNQEAQQWVSGWRLNADSVLWGGHKVFMSKPEEPFQPVKEATQLMNEGGGLE*
	TEV protease: black; amino acid point of insertion: red; P7: green; linker: gray
TEVp_K184_5gs:P7:5gs	MGESLFKGPRDYNPISSTICHLTNESDGHTTSLYGIGFGPFIITNKHLFRRNNGTLLVQSLHGVFKVKNTTTLQQHLIDGRDMIIIRMPKDFPPFPQKLKFREPQREERICLVTTNFQTKSMSSMVSDTSCTFPSSDGIFWKHWIQTKDGQCGSPLVSTRDGFIVGIHSASNFTNTNNYFTSVPKGSSGSEIQALEEKNAQLKQEIAALEEKNQALKYGGSGGSNFMELLTNQEAQQWVSGWRLNADSVLWGGHKVFMSKPEEPFQPVKEATQLMNEGGGLE*
	TEV protease: black; amino acid point of insertion: red; P7: green; linker: gray
TEVp_G212:5gs:P7:5gs	MGESLFKGPRDYNPISSTICHLTNESDGHTTSLYGIGFGPFIITNKHLFRRNNGTLLVQSLHGVFKVKNTTTLQQHLIDGRDMIIIRMPKDFPPFPQKLKFREPQREERICLVTTNFQTKSMSSMVSDTSCTFPSSDGIFWKHWIQTKDGQCGSPLVSTRDGFIVGIHSASNFTNTNNYFTSVPKNFMELLTNQEAQQWVSGWRLNADSVLWGGSSGSEIQALEEKNAQLKQEIAALEEKNQALKYGGSGGSGHKVFMSKPEEPFQPVKEATQLMNEGGGLE*
	TEV protease: black; amino acid point of insertion: red; P7: green; linker: gray
TEVp_I77_5gs:P7:5gs_gs:N8	MGESLFKGPRDYNPISSTICHLTNESDGHTTSLYGIGFGPFIITNKHLFRRNNGTLLVQSLHGVFKVKNTTTLQQHLIGSSGSEIQALEEKNAQLKQEIAALEEKNQALKYGGSGGSDGRDMIIIRMPKDFPPFPQKLKFREPQREERICLVTTNFQTKSMSSMVSDTSCTFPSSDGIFWKHWIQTKDGQCGSPLVSTRDGFIVGIHSASNFTNTNNYFTSVPKNFMELLTNQEAQQWVSGWRLNADSVLWGGHKVFMSKPEEPFQPVKEATQLMNEGGGLEGSPGGSPGGSGSPGGSPGGSKIAALKAENAALEAKIAALKAEIAALEAGY*
	TEV protease: black; amino acid point of insertion: red; P7: green; linker: gray; N8 yellow
TEVp_G27_5gs:P7:5gs_gs:N8	MGESLFKGPRDYNPISSTICHLTNESDGGSSGSEIQALEEKNAQLKQEIAALEEKNQALKYGGSGGSHTTSLYGIGFGPFIITNKHLFRRNNGTLLVQSLHGVFKVKNTTTLQQHLIDGRDMIIIRMPKDFPPFPQKLKFREPQREERICLVTTNFQTKSMSSMVSDTSCTFPSSDGIFWKHWIQTKDGQCGSPLVSTRDGFIVGIHSASNFTNTNNYFTSVPKNFMELLTNQEAQQWVSGWRLNADSVLWGGHKVFMSKPEEPFQPVKEATQLMNEGGGLEGSPGGSPGGSGSPGGSPGGSKIAALKAENAALEAKIAALKAEIAALEAGY*
	TEV protease: black; amino acid point of insertion: red; P7: green; linker: gray; N8 yellow
TEVp_G79_5gs:P7:5gs_gs:N8	MGESLFKGPRDYNPISSTICHLTNESDGHTTSLYGIGFGPFIITNKHLFRRNNGTLLVQSLHGVFKVKNTTTLQQHLIDGGSSGSEIQALEEKNAQLKQEIAALEEKNQALKYGGSGGSRDMIIIRMPKDFPPFPQKLKFREPQREERICLVTTNFQTKSMSSMVSDTSCTFPSSDGIFWKHWIQTKDGQCGSPLVSTRDGFIVGIHSASNFTNTNNYFTSVPKNFMELLTNQEAQQWVSGWRLNADSVLWGGHKVFMSKPEEPFQPVKEATQLMNEGGGLEGSPGGSPGGSGSPGGSPGGSKIAALKAENAALEAKIAALKAEIAALEAGY*
	TEV protease: black; amino acid point of insertion: red; P7: green; linker: gray; N8 yellow
TALE A_T496:5gs:N6:5gs_N8	MHHHHHHDYKDHDGDYKDHDIDYKDDDDKMAPKKKRKVGIHRGVPMVDLRTLGYSQQQQEKIKPKVRSTVAQHHEALVGHGFTHAHIVALSQHPAALGTVAVKYQDMIAALPEATHEAIVGVGKQWSGARALEALLTVAGELRGPPLQLDTGQLLKIAKRGGVTAVEAVHAWRNALTGAPLNLTPDQVVAIASNGGGKQALETVQRLLPVLCQDHGLTPEQVVAIASNGGGKQALETVQRLLPVLCQAHGLTPDQVVAIASNIGGKQALETVQRLLPVLCQAHGLTPAQVVAIASHDGGKQALETVQRLLPVLCQDHGLTPDQVVAIASNGGGKQALETVQRLLPVLCQDHGLTPEQVVAIANNNGGKQALETVQRLLPVLCQAHGLTPDQVVAIASHDGGKQALETVQRLLPVLCQAHGLTPAQVVAIASNGGGKQALETVQRLLPVLCQDHGLTPDQVVAIANNNGGKQALETVQRLLPVLCQDHGLTPEQVVAIASHDGGKQALETVQRLLPVLCQAHGLTGSSGSKIAALKAEIAALEAENAALEAKIAALKAGYGSSGSPDQVVAIASNGGGKQALETVQRLLPVLCQAHGLTPAQVVAIASHDGGKQALETVQRLLPVLCQDHGLTPDQVVAIASHDGGKQALETVQRLLPVLCQDHGLTPEQVVAIASHDGGKQALETVQRLLPVLCQAHGLTPDQVVAIANNNGGKQALETVQRLLPVLCQAHGLTPAQVVAIASHDGGKQALETVQRLLPVLCQDHGLTPEQVVAIASNGGGRPALESIVAQLSRPDPALAALTNDHLVALACLGGRPALDAVKKGLPHAPALIKRTNRRIPERTSHRVAGSDPKKKRKVGGSGGGSGGSYKIAALKAENAALEAKIAALKAEIAALEAGC*
	TALA DNA binging domain: black; amino acid point of insertion: red; N6: Blue; N8 yellow linker: gray
N5:gs_TALE A_T496:5gs:N6:5gs_N8	MGEIAALEAKIAALKAKNAALKAEIAALEAGSPGGSPGGSGSPGGSPGGSGSPGGSPGGSGSPGGSPGGSHHHHHHDYKDHDGDYKDHDIDYKDDDDKMAPKKKRKVGIHRGVPMVDLRTLGYSQQQQEKIKPKVRSTVAQHHEALVGHGFTHAHIVALSQHPAALGTVAVKYQDMIAALPEATHEAIVGVGKQWSGARALEALLTVAGELRGPPLQLDTGQLLKIAKRGGVTAVEAVHAWRNALTGAPLNLTPDQVVAIASNGGGKQALETVQRLLPVLCQDHGLTPEQVVAIASNGGGKQALETVQRLLPVLCQAHGLTPDQVVAIASNIGGKQALETVQRLLPVLCQAHGLTPAQVVAIASHDGGKQALETVQRLLPVLCQDHGLTPDQVVAIASNGGGKQALETVQRLLPVLCQDHGLTPEQVVAIANNNGGKQALETVQRLLPVLCQAHGLTPDQVVAIASHDGGKQALETVQRLLPVLCQAHGLTPAQVVAIASNGGGKQALETVQRLLPVLCQDHGLTPDQVVAIANNNGGKQALETVQRLLPVLCQDHGLTPEQVVAIASHDGGKQALETVQRLLPVLCQAHGLTGSSGSKIAALKAEIAALEAENAALEAKIAALKAGYGSSGSPDQVVAIASNGGGKQALETVQRLLPVLCQAHGLTPAQVVAIASHDGGKQALETVQRLLPVLCQDHGLTPDQVVAIASHDGGKQALETVQRLLPVLCQDHGLTPEQVVAIASHDGGKQALETVQRLLPVLCQAHGLTPDQVVAIANNNGGKQALETVQRLLPVLCQAHGLTPAQVVAIASHDGGKQALETVQRLLPVLCQDHGLTPEQVVAIASNGGGRPALESIVAQLSRPDPALAALTNDHLVALACLGGRPALDAVKKGLPHAPALIKRTNRRIPERTSHRVAGSDPKKKRKVGGSGGGSGGSYKIAALKAENAALEAKIAALKAEIAALEAGC*
	TALA DNA binging domain: black; amino acid point of insertion: red; N6: Blue; N8 yellow; linker: gray; N5 purple; Nuclear Localisatin Sequence: orange
P5A:gs_TALE A_T496:5gs:N6:5gs_N8	MYGENAALEAKIAALKAKNAALKAEIAALEAGGSPGGSPGGSGSPGGSPGGSGSPGGSPGGSGSPGGSPGGSHHHHHHDYKDHDGDYKDHDIDYKDDDDKMAPKKKRKVGIHRGVPMVDLRTLGYSQQQQEKIKPKVRSTVAQHHEALVGHGFTHAHIVALSQHPAALGTVAVKYQDMIAALPEATHEAIVGVGKQWSGARALEALLTVAGELRGPPLQLDTGQLLKIAKRGGVTAVEAVHAWRNALTGAPLNLTPDQVVAIASNGGGKQALETVQRLLPVLCQDHGLTPEQVVAIASNGGGKQALETVQRLLPVLCQAHGLTPDQVVAIASNIGGKQALETVQRLLPVLCQAHGLTPAQVVAIASHDGGKQALETVQRLLPVLCQDHGLTPDQVVAIASNGGGKQALETVQRLLPVLCQDHGLTPEQVVAIANNNGGKQALETVQRLLPVLCQAHGLTPDQVVAIASHDGGKQALETVQRLLPVLCQAHGLTPAQVVAIASNGGGKQALETVQRLLPVLCQDHGLTPDQVVAIANNNGGKQALETVQRLLPVLCQDHGLTPEQVVAIASHDGGKQALETVQRLLPVLCQAHGLTGSSGSKIAALKAEIAALEAENAALEAKIAALKAGYGSSGSPDQVVAIASNGGGKQALETVQRLLPVLCQAHGLTPAQVVAIASHDGGKQALETVQRLLPVLCQDHGLTPDQVVAIASHDGGKQALETVQRLLPVLCQDHGLTPEQVVAIASHDGGKQALETVQRLLPVLCQAHGLTPDQVVAIANNNGGKQALETVQRLLPVLCQAHGLTPAQVVAIASHDGGKQALETVQRLLPVLCQDHGLTPEQVVAIASNGGGRPALESIVAQLSRPDPALAALTNDHLVALACLGGRPALDAVKKGLPHAPALIKRTNRRIPERTSHRVAGSDPKKKRKVGGSGGGSGGSYKIAALKAENAALEAKIAALKAEIAALEAGC*
	TALA DNA binging domain: black; amino acid point of insertion: red; N6: Blue; N8 yellow; linker: gray; P5A dark green; Nuclear Localisatin Sequence: orange
N7:gs:VP16	MGEIAALEAKNAALKAEIAALEAKIAALKAGYGGSGGGSGGSDPKKKRKVAPPTDVSLGDELHLDGEDVAMAHADALDDFDLDMLGDGDSPGPGFTPHDSAPYGALDMADFEFEQMFTDALGIDEYGGDTYRYI*

	N7: dark blue; linker: gray; Nuclear Localisatin Sequence: orange
mCherry:gs:N5:<br>NLS	MEQKLISEEDLMVSKGEEDNMAIIKEFMRFKVHMEGSVNGHEFEIEGEGEGRPYEGTQTAKLKVTKGGPLPFAWDILSPQFMYGSKAYVKHPADIPDYLKLSF<br>PEGFKWERVMNFEDGGVVTVTQDSSLQDGEFIYKVKLRGTNFPSDGPVMQKKTMGWEASSERMYPEDGALKGEIKQRLKLKDGGHYDAEVKTTYKAKKPV<br>QLPGAYNVNIKLDITSHNEDYTIVEQYERAEGRHSTGGMDELYKGGSGGGSGGSGEIAALEAKIAALKAKNAALKAEIAALEADPKKKRKV*
	Myc tag: brown; mCherry: black; N5: violet; linker: gray; Nuclear Localisatin Sequence: orange
mCherry:gs:N6:<br>NLS	MEQKLISEEDLMVSKGEEDNMAIIKEFMRFKVHMEGSVNGHEFEIEGEGEGRPYEGTQTAKLKVTKGGPLPFAWDILSPQFMYGSKAYVKHPADIPDYLKLSF<br>PEGFKWERVMNFEDGGVVTVTQDSSLQDGEFIYKVKLRGTNFPSDGPVMQKKTMGWEASSERMYPEDGALKGEIKQRLKLKDGGHYDAEVKTTYKAKKPV<br>QLPGAYNVNIKLDITSHNEDYTIVEQYERAEGRHSTGGMDELYKGGSGGGSGGSGKIAALKAEIAALEAENAALEAKIAALKAGYDPKKKRKV*
	Myc tag: brown; mCherry: black; N6: Blue ; linker: gray; Nuclear Localisatin Sequence: orange
dCas:VPR_S55<br>_5gs:N8:5gs	MDKKYSIGLAIGTNSVGWAVITDEYKVPSKKFKVLGNTDRHSIKKNLIGALLFDSGSSGSYGKIAALKAENAALEAKIAALKAEIAALEAGGSGGSGETAEATRLK<br>RTARRRYTRRKNRICYLQEIFSNEMAKVDDSFFHRLEESFLVEEDKKHERHPIFGNIVDEVAYHEKYPTIYHLRKKLVDSTDKADLRLIYLALAHMIKFRGHFLIE<br>GDLNPDNSDVDKLFIQLVQTYNQLFEENPINASGVDAKAILSARLSKSRRLENLIAQLPGEKKNGLFGNLIALSLGLTPNFKSNFDLAEDAKLQLSKDTYDDDLD<br>NLLAQIGDQYADLFLAAKNLSDAILLSDILRVNTEITKAPLSASMIKRYDEHHQDLTLLKALVRQQLPEKYKEIFFDQSKNGYAGYIDGGASQEEFYKFIKPILEKM<br>DGTEELLVKLNREDLLRKQRTFDNGSIPHQIHLGELHAILRRQEDFYPFLKDNREKIEKILTFRIPYYVGPLARGNSRFAWMTRKSEETITPWNFEEVVDKGASA<br>QSFIERMTNFDKNLPNEKVLPKHSLLYEYFTVYNELTKVKYVTEGMRKPAFLSGEQKKAIVDLLFKTNRKVTVKQLKEDYFKKIECFDSVEISGVEDRFNASLGT<br>YHDLLKIIKDKDFLDNEENEDILEDIVLTLTLFEDREMIEERLKTYAHLFDDKVMKQLKRRRYTGWGRLSRKLINGIRDKQSGKTILDFLKSDGFANRNFMQLIHD<br>DSLTFKEDIQKAQVSGQGDSLHEHIANLAGSPAIKKGILQTVKVVDELVKVMGRHKPENIVIEMARENQTTQKGQKNSRERMKRIEEGIKELGSQILKEHPVENT<br>QLQNEKLYLYYLQNGRDMYVDQELDINRLSDYDVDAIVPQSFLKDDSIDNKVLTRSDKNRGKSDNVPSEEVVKKMKNYWRQLLNAKLITQRKFDNLTKAERGG<br>LSELDKAGFIKRQLVETRQITKHVAQILDSRMNTKYDENDKLIREVKVITLKSKLVSDFRKDFQFYKVREINNYHHAHDAYLNAVVGTALIKKYPKLESEFVYGDY<br>KVYDVRKMIAKSEQEIGKATAKYFFYSNIMNFFKTEITLANGEIRKRPLIETNGETGEIVWDKGRDFATVRKVLSMPQVNIVKKTEVQTGGFSKESILPKRNSDKLI<br>ARKKDWDPKKYGGFDSPTVAYSVLVVAKVEKGKSKKLKSVKELLGITIMERSSFEKNPIDFLEAKGYKEVKKDLIIKLPKYSLFELENGRKRMLASAGELQKGNE<br>LALPSKYVNFLYLASHYEKLKGSPEDNEQKQLFVEQHKHYLDEIIEQISEFSKRVILADANLDKVLSAYNKHRDKPIREQAENIIHLFTLTNLGAPAAFKYFDTTIDR<br>KRYTSTKEVLDATLIHQSITGLYETRIDLSQLGGDDPKKKRKVEASGSGRADALDDFDLDMLGSDALDDFDLDMLGSDALDDFDLDMLGSDALDDFDLDMLINT<br>SGGSGGGSGGSSQYLPDTDDRHRIEEKRKRTYETFKSIMKKSPFSGPTDPRPPPRRIAVPSRSSASVPKPAPQPYPFTSSLSTINYDEFPTMVFPSGQISQAS<br>ALAPAPPQVLPQAPAPAPAPAMVSALAQAPAPVPVLAPGPPQAVAPPAPKPTQAGEGTLSEALLQLQFDDEDLGALLGNSTDPAVFTDLASVDNSEFQQLLN<br>QGIPVAPHTTEPMLMEYPEAITRLVTGAQRPPDPAPAPLGAPGLPNGLLSGDEDFSSIADMDFSALLGSGSGSRDSREGMFLPKPEAGSAISDVFEGREVCQP<br>KRIRPFHPPGSPWANRPLPASLAPTPTGPVHEPVGSLTPAPVPQPLDPAPAVTPEASHLLEDPDEETSQAVKALREMADTVIPQKEEAAICGQMDLSHPPPRG<br>HLDELTTTLESMTEDLNLDSPLTPELNEILDTFLNDECLLHAMHISTGLSIFDTSLF*
	dCas: black; amino acid point of insertion: red; VPR transcription activation domain : light green; Nuclear Localisatin Sequence: orange ; N8 yellow;<br>linker: gray
dCas:VPR_D14<br>7_5gs:N8:5gs	MDKKYSIGLAIGTNSVGWAVITDEYKVPSKKFKVLGNTDRHSIKKNLIGALLFDSGETAEATRLKRTARRRYTRRKNRICYLQEIFSNEMAKVDDSFFHRLEESF<br>LVEEDKKHERHPIFGNIVDEVAYHEKYPTIYHLRKKLVDSTDGSSGSYGKIAALKAENAALEAKIAALKAEIAALEAGGSGGSKADLRLIYLALAHMIKFRGHFLIE<br>GDLNPDNSDVDKLFIQLVQTYNQLFEENPINASGVDAKAILSARLSKSRRLENLIAQLPGEKKNGLFGNLIALSLGLTPNFKSNFDLAEDAKLQLSKDTYDDDLD<br>NLLAQIGDQYADLFLAAKNLSDAILLSDILRVNTEITKAPLSASMIKRYDEHHQDLTLLKALVRQQLPEKYKEIFFDQSKNGYAGYIDGGASQEEFYKFIKPILEKM<br>DGTEELLVKLNREDLLRKQRTFDNGSIPHQIHLGELHAILRRQEDFYPFLKDNREKIEKILTFRIPYYVGPLARGNSRFAWMTRKSEETITPWNFEEVVDKGASA<br>QSFIERMTNFDKNLPNEKVLPKHSLLYEYFTVYNELTKVKYVTEGMRKPAFLSGEQKKAIVDLLFKTNRKVTVKQLKEDYFKKIECFDSVEISGVEDRFNASLGT<br>YHDLLKIIKDKDFLDNEENEDILEDIVLTLTLFEDREMIEERLKTYAHLFDDKVMKQLKRRRYTGWGRLSRKLINGIRDKQSGKTILDFLKSDGFANRNFMQLIHD<br>DSLTFKEDIQKAQVSGQGDSLHEHIANLAGSPAIKKGILQTVKVVDELVKVMGRHKPENIVIEMARENQTTQKGQKNSRERMKRIEEGIKELGSQILKEHPVENT<br>QLQNEKLYLYYLQNGRDMYVDQELDINRLSDYDVDAIVPQSFLKDDSIDNKVLTRSDKNRGKSDNVPSEEVVKKMKNYWRQLLNAKLITQRKFDNLTKAERGG<br>LSELDKAGFIKRQLVETRQITKHVAQILDSRMNTKYDENDKLIREVKVITLKSKLVSDFRKDFQFYKVREINNYHHAHDAYLNAVVGTALIKKYPKLESEFVYGDY<br>KVYDVRKMIAKSEQEIGKATAKYFFYSNIMNFFKTEITLANGEIRKRPLIETNGETGEIVWDKGRDFATVRKVLSMPQVNIVKKTEVQTGGFSKESILPKRNSDKLI<br>ARKKDWDPKKYGGFDSPTVAYSVLVVAKVEKGKSKKLKSVKELLGITIMERSSFEKNPIDFLEAKGYKEVKKDLIIKLPKYSLFELENGRKRMLASAGELQKGNE<br>LALPSKYVNFLYLASHYEKLKGSPEDNEQKQLFVEQHKHYLDEIIEQISEFSKRVILADANLDKVLSAYNKHRDKPIREQAENIIHLFTLTNLGAPAAFKYFDTTIDR<br>KRYTSTKEVLDATLIHQSITGLYETRIDLSQLGGDDPKKKRKVEASGSGRADALDDFDLDMLGSDALDDFDLDMLGSDALDDFDLDMLGSDALDDFDLDMLINT<br>SGGSGGGSGGSSQYLPDTDDRHRIEEKRKRTYETFKSIMKKSPFSGPTDPRPPPRRIAVPSRSSASVPKPAPQPYPFTSSLSTINYDEFPTMVFPSGQISQAS<br>ALAPAPPQVLPQAPAPAPAPAMVSALAQAPAPVPVLAPGPPQAVAPPAPKPTQAGEGTLSEALLQLQFDDEDLGALLGNSTDPAVFTDLASVDNSEFQQLLN<br>QGIPVAPHTTEPMLMEYPEAITRLVTGAQRPPDPAPAPLGAPGLPNGLLSGDEDFSSIADMDFSALLGSGSGSRDSREGMFLPKPEAGSAISDVFEGREVCQP<br>KRIRPFHPPGSPWANRPLPASLAPTPTGPVHEPVGSLTPAPVPQPLDPAPAVTPEASHLLEDPDEETSQAVKALREMADTVIPQKEEAAICGQMDLSHPPPRG<br>HLDELTTTLESMTEDLNLDSPLTPELNEILDTFLNDECLLHAMHISTGLSIFDTSLF*
	dCas: black; amino acid point of insertion: red; VPR transcription activation domain : light green ; Nuclear Localisatin Sequence: orange ; N8 yellow;<br>linker: gray
dCas:VPR_R53<br>5_5gs:N8:5gs	MDKKYSIGLAIGTNSVGWAVITDEYKVPSKKFKVLGNTDRHSIKKNLIGALLFDSGETAEATRLKRTARRRYTRRKNRICYLQEIFSNEMAKVDDSFFHRLEESF<br>LVEEDKKHERHPIFGNIVDEVAYHEKYPTIYHLRKKLVDSTDKADLRLIYLALAHMIKFRGHFLIEGDLNPDNSDVDKLFIQLVQTYNQLFEENPINASGVDAKAIL<br>SARLSKSRRLENLIAQLPGEKKNGLFGNLIALSLGLTPNFKSNFDLAEDAKLQLSKDTYDDDLDNLLAQIGDQYADLFLAAKNLSDAILLSDILRVNTEITKAPLSA<br>SMIKRYDEHHQDLTLLKALVRQQLPEKYKEIFFDQSKNGYAGYIDGGASQEEFYKFIKPILEKMDGTEELLVKLNREDLLRKQRTFDNGSIPHQIHLGELHAILRR<br>QEDFYPFLKDNREKIEKILTFRIPYYVGPLARGNSRFAWMTRKSEETITPWNFEEVVDKGASAQSFIERMTNFDKNLPNEKVLPKHSLLYEYFTVYNELTKVKYV<br>TEGMRGSSGSYGKIAALKAENAALEAKIAALKAEIAALEAGGSGGSKPAFLSGEQKKAIVDLLFKTNRKVTVKQLKEDYFKKIECFDSVEISGVEDRFNASLGTY<br>HDLLKIIKDKDFLDNEENEDILEDIVLTLTLFEDREMIEERLKTYAHLFDDKVMKQLKRRRYTGWGRLSRKLINGIRDKQSGKTILDFLKSDGFANRNFMQLIHDD<br>SLTFKEDIQKAQVSGQGDSLHEHIANLAGSPAIKKGILQTVKVVDELVKVMGRHKPENIVIEMARENQTTQKGQKNSRERMKRIEEGIKELGSQILKEHPVENTQ<br>LQNEKLYLYYLQNGRDMYVDQELDINRLSDYDVDAIVPQSFLKDDSIDNKVLTRSDKNRGKSDNVPSEEVVKKMKNYWRQLLNAKLITQRKFDNLTKAERGGL<br>SELDKAGFIKRQLVETRQITKHVAQILDSRMNTKYDENDKLIREVKVITLKSKLVSDFRKDFQFYKVREINNYHHAHDAYLNAVVGTALIKKYPKLESEFVYGDYK<br>VYDVRKMIAKSEQEIGKATAKYFFYSNIMNFFKTEITLANGEIRKRPLIETNGETGEIVWDKGRDFATVRKVLSMPQVNIVKKTEVQTGGFSKESILPKRNSDKLIA<br>RKKDWDPKKYGGFDSPTVAYSVLVVAKVEKGKSKKLKSVKELLGITIMERSSFEKNPIDFLEAKGYKEVKKDLIIKLPKYSLFELENGRKRMLASAGELQKGNEL<br>ALPSKYVNFLYLASHYEKLKGSPEDNEQKQLFVEQHKHYLDEIIEQISEFSKRVILADANLDKVLSAYNKHRDKPIREQAENIIHLFTLTNLGAPAAFKYFDTTIDR<br>KRYTSTKEVLDATLIHQSITGLYETRIDLSQLGGDDPKKKRKVEASGSGRADALDDFDLDMLGSDALDDFDLDMLGSDALDDFDLDMLGSDALDDFDLDMLINT<br>SGGSGGGSGGSSQYLPDTDDRHRIEEKRKRTYETFKSIMKKSPFSGPTDPRPPPRRIAVPSRSSASVPKPAPQPYPFTSSLSTINYDEFPTMVFPSGQISQAS<br>ALAPAPPQVLPQAPAPAPAPAMVSALAQAPAPVPVLAPGPPQAVAPPAPKPTQAGEGTLSEALLQLQFDDEDLGALLGNSTDPAVFTDLASVDNSEFQQLLN<br>QGIPVAPHTTEPMLMEYPEAITRLVTGAQRPPDPAPAPLGAPGLPNGLLSGDEDFSSIADMDFSALLGSGSGSRDSREGMFLPKPEAGSAISDVFEGREVCQP<br>KRIRPFHPPGSPWANRPLPASLAPTPTGPVHEPVGSLTPAPVPQPLDPAPAVTPEASHLLEDPDEETSQAVKALREMADTVIPQKEEAAICGQMDLSHPPPRG<br>HLDELTTTLESMTEDLNLDSPLTPELNEILDTFLNDECLLHAMHISTGLSIFDTSLF*
	dCas: black; amino acid point of insertion: red; VPR transcription activation domain : light green ; Nuclear Localisatin Sequence: orange ; N8 yellow;<br>linker: gray

dCas:VPR_G1104_5gs:N8:5gs	DKKYSIGLAIGTNSVGWAVITDEYKVPSKKFKVLGNTDRHSIKKNLIGALLFDSGETAEATRLKRTARRRYTRRKNRICYLQEIFSNEMAKVDDSFFHRLEESFLV<br>EEDKKHERHPIFGNIVDEVAYHEKYPTIYHLRKKLVDSTDKADLRLIYLALAHMIKFRGHFLIEGDLNPDNSDVDKLFIQLVQTYNQLFEENPINASGVDAKAILSA<br>RLSKSRRLENLIAQLPGEKKNGLFGNLIALSLGLTPNFKSNFDLAEDAKLQLSKDTYDDDLDNLLAQIGDQYADLFLAAKNLSDAILLSDILRVNTEITKAPLSASMI<br>KRYDEHHQDLTLLKALVRQQLPEKYKEIFFDQSKNGYAGYIDGGASQEEFYKFIKPILEKMDGTEELLVKLNREDLLRKQRTFDNGSIPHQIHLGELHAILRRQE<br>DFYPFLKDNREKIEKILTFRIPYYVGPLARGNSRFAWMTRKSEETITPWNFEEVVDKGASAQSFIERMTNFDKNLPNEKVLPKHSLLYEYFTVYNELTKVKYVTE<br>GMRKPAFLSGEQKKAIVDLLFKTNRKVTVKQLKEDYFKKIECFDSVEISGVEDRFNASLGTYHDLLKIIKDKDFLDNEENEDILEDIVLTLTLFEDREMIEERLKTY<br>AHLFDDKVMKQLKRRRYTGWGRLSRKLINGIRDKQSGKTILDFLKSDGFANRNFMQLIHDDSLTFKEDIQKAQVSGQGDSLHEHIANLAGSPAIKKGILQTVKVV<br>DELVKVMGRHKPENIVIEMARENQTTQKGQKNSRERMKRIEEGIKELGSQILKEHPVENTQLQNEKLYLYYLQNGRDMYVDQELDINRLSDYDVDAIVPQSFLK<br>DDSIDNKVLTRSDKNRGKSDNVPSEEVVKKMKNYWRQLLNAKLITQRKFDNLTKAERGGLSELDKAGFIKRQLVETRQITKHVAQILDSRMNTKYDENDKLIRE<br>VKVITLKSKLVSDFRKDFQFYKVREINNYHHAHDAYLNAVVGTALIKKYPKLESEFVYGDYKVYDVRKMIAKSEQEIGKATAKYFFYSNIMNFFKTEITLANGEIRK<br>RPLIETNGETGEIVWDKGRDFATVRKVLSMPQVNIVKKTEVQTGGGSSGSYGKIAALKAENAALEAKIAALKAEIAALEAGGSGGSFSKESILPKRNSDKLIARK<br>KDWDPKKYGGFDSPTVAYSVLVVAKVEKGKSKKLKSVKELLGITIMERSSFEKNPIDFLEAKGYKEVKKDLIIKLPKYSLFELENGRKRMLASAGELQKGNELAL<br>PSKYVNFLYLASHYEKLKGSPEDNEQKQLFVEQHKHYLDEIIEQISEFSKRVILADANLDKVLSAYNKHRDKPIREQAENIIHLFTLTNLGAPAAFKYFDTTIDRKR<br>YTSTKEVLDATLIHQSITGLYETRIDLSQLGGDDPKKKRKVEASGSGRADALDDFDLDMLGSDALDDFDLDMLGSDALDDFDLDMLGSDALDDFDLDMLINTSG<br>GSGGGSGGSSQYLPDTDDRHRIEEKRKRTYETFKSIMKKSPFSGPTDPRPPPRRIAVPSRSSASVPKPAPQPYPFTSSLSTINYDEFPTMVFPSGQISQASALA<br>PAPPQVLPQAPAPAPAPAMVSALAQAPAPVPVLAPGPPQAVAPPAPKPTQAGEGTLSEALLQLQFDDEDLGALLGNSTDPAVFTDLASVDNSEFQQLLNQGI<br>PVAPHTTEPMLMEYPEAITRLVTGAQRPPDPAPAPLGAPGLPNGLLSGDEDFSSIADMDFSALLGSGSGSRDSREGMFLPKPEAGSAISDVFEGREVCQPKRI<br>RPFHPPGSPWANRPLPASLAPTPTGPVHEPVGSLTPAPVPQPLDPAPAVTPEASHLLEDPDEETSQAVKALREMADTVIPQKEEAAICGQMDLSHPPPRGHL<br>DELTTTLESMTEDLNLDSPLTPELNEILDTFLNDECLLHAMHISTGLSIFDTSLF*
	dCas: black; amino acid point of insertion: red; VPR transcription activation domain : light green ; Nuclear Localisatin Sequence: orange ; N8 yellow; linker: gray
dCas:VPR_K1153_5gs:N8:5gs	DKKYSIGLAIGTNSVGWAVITDEYKVPSKKFKVLGNTDRHSIKKNLIGALLFDSGETAEATRLKRTARRRYTRRKNRICYLQEIFSNEMAKVDDSFFHRLEESFLV<br>EEDKKHERHPIFGNIVDEVAYHEKYPTIYHLRKKLVDSTDKADLRLIYLALAHMIKFRGHFLIEGDLNPDNSDVDKLFIQLVQTYNQLFEENPINASGVDAKAILSA<br>RLSKSRRLENLIAQLPGEKKNGLFGNLIALSLGLTPNFKSNFDLAEDAKLQLSKDTYDDDLDNLLAQIGDQYADLFLAAKNLSDAILLSDILRVNTEITKAPLSASMI<br>KRYDEHHQDLTLLKALVRQQLPEKYKEIFFDQSKNGYAGYIDGGASQEEFYKFIKPILEKMDGTEELLVKLNREDLLRKQRTFDNGSIPHQIHLGELHAILRRQE<br>DFYPFLKDNREKIEKILTFRIPYYVGPLARGNSRFAWMTRKSEETITPWNFEEVVDKGASAQSFIERMTNFDKNLPNEKVLPKHSLLYEYFTVYNELTKVKYVTE<br>GMRKPAFLSGEQKKAIVDLLFKTNRKVTVKQLKEDYFKKIECFDSVEISGVEDRFNASLGTYHDLLKIIKDKDFLDNEENEDILEDIVLTLTLFEDREMIEERLKTY<br>AHLFDDKVMKQLKRRRYTGWGRLSRKLINGIRDKQSGKTILDFLKSDGFANRNFMQLIHDDSLTFKEDIQKAQVSGQGDSLHEHIANLAGSPAIKKGILQTVKVV<br>DELVKVMGRHKPENIVIEMARENQTTQKGQKNSRERMKRIEEGIKELGSQILKEHPVENTQLQNEKLYLYYLQNGRDMYVDQELDINRLSDYDVDAIVPQSFLK<br>DDSIDNKVLTRSDKNRGKSDNVPSEEVVKKMKNYWRQLLNAKLITQRKFDNLTKAERGGLSELDKAGFIKRQLVETRQITKHVAQILDSRMNTKYDENDKLIRE<br>VKVITLKSKLVSDFRKDFQFYKVREINNYHHAHDAYLNAVVGTALIKKYPKLESEFVYGDYKVYDVRKMIAKSEQEIGKATAKYFFYSNIMNFFKTEITLANGEIRK<br>RPLIETNGETGEIVWDKGRDFATVRKVLSMPQVNIVKKTEVQTGGFSKESILPKRNSDKLIARKKDWDPKKYGGFDSPTVAYSVLVVAKVEKGKSKGSSGSYG<br>KIAALKAENAALEAKIAALKAEIAALEAGGSGGSKLKSVKELLGITIMERSSFEKNPIDFLEAKGYKEVKKDLIIKLPKYSLFELENGRKRMLASAGELQKGNELALP<br>SKYVNFLYLASHYEKLKGSPEDNEQKQLFVEQHKHYLDEIIEQISEFSKRVILADANLDKVLSAYNKHRDKPIREQAENIIHLFTLTNLGAPAAFKYFDTTIDRKRY<br>TSTKEVLDATLIHQSITGLYETRIDLSQLGGDDPKKKRKVEASGSGRADALDDFDLDMLGSDALDDFDLDMLGSDALDDFDLDMLGSDALDDFDLDMLINTSG<br>GSGGGSGGSSQYLPDTDDRHRIEEKRKRTYETFKSIMKKSPFSGPTDPRPPPRRIAVPSRSSASVPKPAPQPYPFTSSLSTINYDEFPTMVFPSGQISQASALA<br>PAPPQVLPQAPAPAPAPAMVSALAQAPAPVPVLAPGPPQAVAPPAPKPTQAGEGTLSEALLQLQFDDEDLGALLGNSTDPAVFTDLASVDNSEFQQLLNQGI<br>PVAPHTTEPMLMEYPEAITRLVTGAQRPPDPAPAPLGAPGLPNGLLSGDEDFSSIADMDFSALLGSGSGSRDSREGMFLPKPEAGSAISDVFEGREVCQPKRI<br>RPFHPPGSPWANRPLPASLAPTPTGPVHEPVGSLTPAPVPQPLDPAPAVTPEASHLLEDPDEETSQAVKALREMADTVIPQKEEAAICGQMDLSHPPPRGHL<br>DELTTTLESMTEDLNLDSPLTPELNEILDTFLNDECLLHAMHISTGLSIFDTSLF*
	dCas: black; amino acid point of insertion: red; VPR transcription activation domain : light green; orange: Nuclear Localisatin Sequence; N8 yellow; linker: gray
N8:gs40_dCas:VPR_R535_5gs:P7:5gs	MKIAALKAENAALEAKIAALKAEIAALEAGYGSPGGSPGGSGSPGGSPGGSGSPGGSPGGSGSPGGSPGGSDKKYSIGLAIGTNSVGWAVITDEYKVPSKKF<br>KVLGNTDRHSIKKNLIGALLFDSGETAEATRLKRTARRRYTRRKNRICYLQEIFSNEMAKVDDSFFHRLEESFLVEEDKKHERHPIFGNIVDEVAYHEKYPTIYHL<br>RKKLVDSTDKADLRLIYLALAHMIKFRGHFLIEGDLNPDNSDVDKLFIQLVQTYNQLFEENPINASGVDAKAILSARLSKSRRLENLIAQLPGEKKNGLFGNLIALS<br>LGLTPNFKSNFDLAEDAKLQLSKDTYDDDLDNLLAQIGDQYADLFLAAKNLSDAILLSDILRVNTEITKAPLSASMIKRYDEHHQDLTLLKALVRQQLPEKYKEIFF<br>DQSKNGYAGYIDGGASQEEFYKFIKPILEKMDGTEELLVKLNREDLLRKQRTFDNGSIPHQIHLGELHAILRRQEDFYPFLKDNREKIEKILTFRIPYYVGPLARG<br>NSRFAWMTRKSEETITPWNFEEVVDKGASAQSFIERMTNFDKNLPNEKVLPKHSLLYEYFTVYNELTKVKYVTEGMRGSSGSEIQALEEKNAQLKQEIAALEE<br>KNQALKYGGSGGSKPAFLSGEQKKAIVDLLFKTNRKVTVKQLKEDYFKKIECFDSVEISGVEDRFNASLGTYHDLLKIIKDKDFLDNEENEDILEDIVLTLTLFEDR<br>EMIEERLKTYAHLFDDKVMKQLKRRRYTGWGRLSRKLINGIRDKQSGKTILDFLKSDGFANRNFMQLIHDDSLTFKEDIQKAQVSGQGDSLHEHIANLAGSPAIK<br>KGILQTVKVVDELVKVMGRHKPENIVIEMARENQTTQKGQKNSRERMKRIEEGIKELGSQILKEHPVENTQLQNEKLYLYYLQNGRDMYVDQELDINRLSDYDV<br>DAIVPQSFLKDDSIDNKVLTRSDKNRGKSDNVPSEEVVKKMKNYWRQLLNAKLITQRKFDNLTKAERGGLSELDKAGFIKRQLVETRQITKHVAQILDSRMNTK<br>YDENDKLIREVKVITLKSKLVSDFRKDFQFYKVREINNYHHAHDAYLNAVVGTALIKKYPKLESEFVYGDYKVYDVRKMIAKSEQEIGKATAKYFFYSNIMNFFKT<br>EITLANGEIRKRPLIETNGETGEIVWDKGRDFATVRKVLSMPQVNIVKKTEVQTGGFSKESILPKRNSDKLIARKKDWDPKKYGGFDSPTVAYSVLVVAKVEKGK<br>SKKLKSVKELLGITIMERSSFEKNPIDFLEAKGYKEVKKDLIIKLPKYSLFELENGRKRMLASAGELQKGNELALPSKYVNFLYLASHYEKLKGSPEDNEQKQLFV<br>EQHKHYLDEIIEQISEFSKRVILADANLDKVLSAYNKHRDKPIREQAENIIHLFTLTNLGAPAAFKYFDTTIDRKRYTSTKEVLDATLIHQSITGLYETRIDLSQLGGD<br>DPKKKRKVEASGSGRADALDDFDLDMLGSDALDDFDLDMLGSDALDDFDLDMLGSDALDDFDLDMLINTSGGSGGGSGGSSQYLPDTDDRHRIEEKRKRTY<br>ETFKSIMKKSPFSGPTDPRPPPRRIAVPSRSSASVPKPAPQPYPFTSSLSTINYDEFPTMVFPSGQISQASALAPAPPQVLPQAPAPAPAPAMVSALAQAPAPV<br>PVLAPGPPQAVAPPAPKPTQAGEGTLSEALLQLQFDDEDLGALLGNSTDPAVFTDLASVDNSEFQQLLNQGIPVAPHTTEPMLMEYPEAITRLVTGAQRPPDP<br>APAPLGAPGLPNGLLSGDEDFSSIADMDFSALLGSGSGSRDSREGMFLPKPEAGSAISDVFEGREVCQPKRIRPFHPPGSPWANRPLPASLAPTPTGPVHEP<br>VGSLTPAPVPQPLDPAPAVTPEASHLLEDPDEETSQAVKALREMADTVIPQKEEAAICGQMDLSHPPPRGHLDELTTTLESMTEDLNLDSPLTPELNEILDTFLN<br>DECLLHAMHISTGLSIFDTSLF-
	dCas: black; amino acid point of insertion: red; VPR transcription activation domain : light green; Nuclear Localisatin Sequence: orange ; N8 yellow; linker: gray P7: green
N8:gs40_dCas:VPR_K1153_5gs:P7:5gs	MKIAALKAENAALEAKIAALKAEIAALEAGYGSPGGSPGGSGSPGGSPGGSGSPGGSPGGSGSPGGSPGGSDKKYSIGLAIGTNSVGWAVITDEYKVPSKKF<br>KVLGNTDRHSIKKNLIGALLFDSGETAEATRLKRTARRRYTRRKNRICYLQEIFSNEMAKVDDSFFHRLEESFLVEEDKKHERHPIFGNIVDEVAYHEKYPTIYHL<br>RKKLVDSTDKADLRLIYLALAHMIKFRGHFLIEGDLNPDNSDVDKLFIQLVQTYNQLFEENPINASGVDAKAILSARLSKSRRLENLIAQLPGEKKNGLFGNLIALS<br>LGLTPNFKSNFDLAEDAKLQLSKDTYDDDLDNLLAQIGDQYADLFLAAKNLSDAILLSDILRVNTEITKAPLSASMIKRYDEHHQDLTLLKALVRQQLPEKYKEIFF<br>DQSKNGYAGYIDGGASQEEFYKFIKPILEKMDGTEELLVKLNREDLLRKQRTFDNGSIPHQIHLGELHAILRRQEDFYPFLKDNREKIEKILTFRIPYYVGPLARG<br>NSRFAWMTRKSEETITPWNFEEVVDKGASAQSFIERMTNFDKNLPNEKVLPKHSLLYEYFTVYNELTKVKYVTEGMRKPAFLSGEQKKAIVDLLFKTNRKVTVK<br>QLKEDYFKKIECFDSVEISGVEDRFNASLGTYHDLLKIIKDKDFLDNEENEDILEDIVLTLTLFEDREMIEERLKTYAHLFDDKVMKQLKRRRYTGWGRLSRKLIN<br>GIRDKQSGKTILDFLKSDGFANRNFMQLIHDDSLTFKEDIQKAQVSGQGDSLHEHIANLAGSPAIKKGILQTVKVVDELVKVMGRHKPENIVIEMARENQTTQKG<br>QKNSRERMKRIEEGIKELGSQILKEHPVENTQLQNEKLYLYYLQNGRDMYVDQELDINRLSDYDVDAIVPQSFLKDDSIDNKVLTRSDKNRGKSDNVPSEEVVK<br>KMKNYWRQLLNAKLITQRKFDNLTKAERGGLSELDKAGFIKRQLVETRQITKHVAQILDSRMNTKYDENDKLIREVKVITLKSKLVSDFRKDFQFYKVREINNYH<br>HAHDAYLNAVVGTALIKKYPKLESEFVYGDYKVYDVRKMIAKSEQEIGKATAKYFFYSNIMNFFKTEITLANGEIRKRPLIETNGETGEIVWDKGRDFATVRKVLS<br>MPQVNIVKKTEVQTGGFSKESILPKRNSDKLIARKKDWDPKKYGGFDSPTVAYSVLVVAKVEKGKSKGSSGSEIQALEEKNAQLKQEIAALEEKNQALKYGGS<br>GGSKLKSVKELLGITIMERSSFEKNPIDFLEAKGYKEVKKDLIIKLPKYSLFELENGRKRMLASAGELQKGNELALPSKYVNFLYLASHYEKLKGSPEDNEQKQLF<br>VEQHKHYLDEIIEQISEFSKRVILADANLDKVLSAYNKHRDKPIREQAENIIHLFTLTNLGAPAAFKYFDTTIDRKRYTSTKEVLDATLIHQSITGLYETRIDLSQLGG<br>DDPKKKRKVEASGSGRADALDDFDLDMLGSDALDDFDLDMLGSDALDDFDLDMLGSDALDDFDLDMLINTSGGSGGGSGGSSQYLPDTDDRHRIEEKRKRT<br>YETFKSIMKKSPFSGPTDPRPPPRRIAVPSRSSASVPKPAPQPYPFTSSLSTINYDEFPTMVFPSGQISQASALAPAPPQVLPQAPAPAPAPAMVSALAQAPAP<br>VPVLAPGPPQAVAPPAPKPTQAGEGTLSEALLQLQFDDEDLGALLGNSTDPAVFTDLASVDNSEFQQLLNQGIPVAPHTTEPMLMEYPEAITRLVTGAQRPPD<br>PAPAPLGAPGLPNGLLSGDEDFSSIADMDFSALLGSGSGSRDSREGMFLPKPEAGSAISDVFEGREVCQPKRIRPFHPPGSPWANRPLPASLAPTPTGPVHE<br>PVGSLTPAPVPQPLDPAPAVTPEASHLLEDPDEETSQAVKALREMADTVIPQKEEAAICGQMDLSHPPPRGHLDELTTTLESMTEDLNLDSPLTPELNEILDTFL<br>NDECLLHAMHISTGLSIFDTSLF-

	dCas: black; amino acid point of insertion: red; VPR transcription activation domain : light green ; orange: Nuclear Localisatin Sequence; N8 yellow; linker: gray P7: green
N7:NLS	MEQKLISEEDLGEIAALEAKNAALKAEIAALEAKIAALKAGYDPKKKRKV*
	Myc tag: brown; N7: dark blue; linker: gray; Nuclear Localisatin Sequence: orange:
β-gal_L25_5gs:N8:5gs	MVLQRRDWENPGVTQLGSSGSYGKIAALKAENAALEAKIAALKAEIAALEAGGSGGNRLAAHPPFASWRNSEEARTDRPSQQLRSLNGEWRFAWFPAPEAV<br>PESWLECDLPEADTVVVPSNWQMHGYDAPIYTNVTYPITVNPPFVPTENPTGCYSLTFNVDESWLQEGQTRIIFDGVNSAFHLWCNGRWVGYGQDSRLPSE<br>FDLSAFLRAGENRLAVMVLRWSDGSYLEDQDMWRMSGIFRDVSLLHKPTTQISDFHVATRFNDDFSRAVLEAEVQMCGELRDYLRVTVSLWQGETQVASGT<br>APFGGEIIDERGGYADRVTLRLNVENPKLWSAEIPNLYRAVVELHTADGTLIEAEACDVGFREVRIENGLLLLNGKPLLIRGVNRHEHHPLHGQVMDEQTMVQD<br>ILLMKQNNFNAVRCSHYPNHPLWYTLCDRYGLYVVDEANIETHGMVPMNRLTDDPRWLPAMSERVTRMVQRDRNHPSVIIWSLGNESGHGANHDALYRWIK<br>SVDPSRPVQYEGGGADTTATDIICPMYARVDEDQPFPAVPKWSIKKWLSLPGETRPLILCEYAHAMGNSLGGFAKYWQAFRQYPRLQGGFVWDWVDQSLIK<br>YDENGNPWSAYGGDFGDTPNDRQFCMNGLVFADRTPHPALTEAKHQQQFFQFRLSGQTIEVTSEYLFRHSDNELLHWMVALDGKPLASGEVPLDVAPQGK<br>QLIELPELPQPESAGQLWLTVRVVQPNATAWSEAGHISAWQQWRLAENLSVTLPAASHAIPHLTTSEMDFCIELGNKRWQFNRQSGFLSQMWIGDKKQLLTP<br>LRDQFTRAPLDNDIGVSEATRIDPNAWVERWKAAGHYQAEAALLQCTADTLADAVLITTAHAWQHQGKTLFISRKTYRIDGSGQMAITVDVEVASDTPHPARIG<br>LNCQLAQVAERVNWLGLGPQENYPDRLTAACFDRWDLPLSDMYTPYVFPSENGLRCGTRELNYGPHQWRGDFQFNISRYSQQQLMETSHRHLLHAEEGT<br>WLNIDGFHMGIGGDDSWSPSVSAEFQLSAGRYHYQLVWCQKYPYDVPDYA-
	β-gal: black; amino acid point of insertion: red; N8 yellow; linker: gray
β-gal_A35_5gs:N8:5gs	MVLQRRDWENPGVTQLNRLAAHPPFAGSSGSYGKIAALKAENAALEAKIAALKAEIAALEAGGSGGSSWRNSEEARTDRPSQQLRSLNGEWRFAWFPAPEA<br>VPESWLECDLPEADTVVVPSNWQMHGYDAPIYTNVTYPITVNPPFVPTENPTGCYSLTFNVDESWLQEGQTRIIFDGVNSAFHLWCNGRWVGYGQDSRLPS<br>EFDLSAFLRAGENRLAVMVLRWSDGSYLEDQDMWRMSGIFRDVSLLHKPTTQISDFHVATRFNDDFSRAVLEAEVQMCGELRDYLRVTVSLWQGETQVASG<br>TAPFGGEIIDERGGYADRVTLRLNVENPKLWSAEIPNLYRAVVELHTADGTLIEAEACDVGFREVRIENGLLLLNGKPLLIRGVNRHEHHPLHGQVMDEQTMVQ<br>DILLMKQNNFNAVRCSHYPNHPLWYTLCDRYGLYVVDEANIETHGMVPMNRLTDDPRWLPAMSERVTRMVQRDRNHPSVIIWSLGNESGHGANHDALYRWI<br>KSVDPSRPVQYEGGGADTTATDIICPMYARVDEDQPFPAVPKWSIKKWLSLPGETRPLILCEYAHAMGNSLGGFAKYWQAFRQYPRLQGGFVWDWVDQSLI<br>KYDENGNPWSAYGGDFGDTPNDRQFCMNGLVFADRTPHPALTEAKHQQQFFQFRLSGQTIEVTSEYLFRHSDNELLHWMVALDGKPLASGEVPLDVAPQG<br>KQLIELPELPQPESAGQLWLTVRVVQPNATAWSEAGHISAWQQWRLAENLSVTLPAASHAIPHLTTSEMDFCIELGNKRWQFNRQSGFLSQMWIGDKKQLLT<br>PLRDQFTRAPLDNDIGVSEATRIDPNAWVERWKAAGHYQAEAALLQCTADTLADAVLITTAHAWQHQGKTLFISRKTYRIDGSGQMAITVDVEVASDTPHPARI<br>GLNCQLAQVAERVNWLGLGPQENYPDRLTAACFDRWDLPLSDMYTPYVFPSENGLRCGTRELNYGPHQWRGDFQFNISRYSQQQLMETSHRHLLHAEEGT<br>WLNIDGFHMGIGGDDSWSPSVSAEFQLSAGRYHYQLVWCQKYPYDVPDYA-
	β-gal: black; amino acid point of insertion: red; N8 yellow; linker: gray
β-gal_T45_5gs:N8:5gs	MVLQRRDWENPGVTQLNRLAAHPPFASWRNSEEARTGSSGSYGKIAALKAENAALEAKIAALKAEIAALEAGGSGGDRPSQQLRSLNGEWRFAWFPAPEAV<br>PESWLECDLPEADTVVVPSNWQMHGYDAPIYTNVTYPITVNPPFVPTENPTGCYSLTFNVDESWLQEGQTRIIFDGVNSAFHLWCNGRWVGYGQDSRLPSE<br>FDLSAFLRAGENRLAVMVLRWSDGSYLEDQDMWRMSGIFRDVSLLHKPTTQISDFHVATRFNDDFSRAVLEAEVQMCGELRDYLRVTVSLWQGETQVASGT<br>APFGGEIIDERGGYADRVTLRLNVENPKLWSAEIPNLYRAVVELHTADGTLIEAEACDVGFREVRIENGLLLLNGKPLLIRGVNRHEHHPLHGQVMDEQTMVQD<br>ILLMKQNNFNAVRCSHYPNHPLWYTLCDRYGLYVVDEANIETHGMVPMNRLTDDPRWLPAMSERVTRMVQRDRNHPSVIIWSLGNESGHGANHDALYRWIK<br>SVDPSRPVQYEGGGADTTATDIICPMYARVDEDQPFPAVPKWSIKKWLSLPGETRPLILCEYAHAMGNSLGGFAKYWQAFRQYPRLQGGFVWDWVDQSLIK<br>YDENGNPWSAYGGDFGDTPNDRQFCMNGLVFADRTPHPALTEAKHQQQFFQFRLSGQTIEVTSEYLFRHSDNELLHWMVALDGKPLASGEVPLDVAPQGK<br>QLIELPELPQPESAGQLWLTVRVVQPNATAWSEAGHISAWQQWRLAENLSVTLPAASHAIPHLTTSEMDFCIELGNKRWQFNRQSGFLSQMWIGDKKQLLTP<br>LRDQFTRAPLDNDIGVSEATRIDPNAWVERWKAAGHYQAEAALLQCTADTLADAVLITTAHAWQHQGKTLFISRKTYRIDGSGQMAITVDVEVASDTPHPARIG<br>LNCQLAQVAERVNWLGLGPQENYPDRLTAACFDRWDLPLSDMYTPYVFPSENGLRCGTRELNYGPHQWRGDFQFNISRYSQQQLMETSHRHLLHAEEGT<br>WLNIDGFHMGIGGDDSWSPSVSAEFQLSAGRYHYQLVWCQKYPYDVPDYA-
	β-gal: black; amino acid point of insertion: red; N8 yellow; linker: gray
β-gal_L55_5gs:N8:5gs	MVLQRRDWENPGVTQLNRLAAHPPFASWRNSEEARTDRPSQQLRSLGSSGSYGKIAALKAENAALEAKIAALKAEIAALEAGGSGGNGEWRFAWFPAPEAV<br>PESWLECDLPEADTVVVPSNWQMHGYDAPIYTNVTYPITVNPPFVPTENPTGCYSLTFNVDESWLQEGQTRIIFDGVNSAFHLWCNGRWVGYGQDSRLPSE<br>FDLSAFLRAGENRLAVMVLRWSDGSYLEDQDMWRMSGIFRDVSLLHKPTTQISDFHVATRFNDDFSRAVLEAEVQMCGELRDYLRVTVSLWQGETQVASGT<br>APFGGEIIDERGGYADRVTLRLNVENPKLWSAEIPNLYRAVVELHTADGTLIEAEACDVGFREVRIENGLLLLNGKPLLIRGVNRHEHHPLHGQVMDEQTMVQD<br>ILLMKQNNFNAVRCSHYPNHPLWYTLCDRYGLYVVDEANIETHGMVPMNRLTDDPRWLPAMSERVTRMVQRDRNHPSVIIWSLGNESGHGANHDALYRWIK<br>SVDPSRPVQYEGGGADTTATDIICPMYARVDEDQPFPAVPKWSIKKWLSLPGETRPLILCEYAHAMGNSLGGFAKYWQAFRQYPRLQGGFVWDWVDQSLIK<br>YDENGNPWSAYGGDFGDTPNDRQFCMNGLVFADRTPHPALTEAKHQQQFFQFRLSGQTIEVTSEYLFRHSDNELLHWMVALDGKPLASGEVPLDVAPQGK<br>QLIELPELPQPESAGQLWLTVRVVQPNATAWSEAGHISAWQQWRLAENLSVTLPAASHAIPHLTTSEMDFCIELGNKRWQFNRQSGFLSQMWIGDKKQLLTP<br>LRDQFTRAPLDNDIGVSEATRIDPNAWVERWKAAGHYQAEAALLQCTADTLADAVLITTAHAWQHQGKTLFISRKTYRIDGSGQMAITVDVEVASDTPHPARIG<br>LNCQLAQVAERVNWLGLGPQENYPDRLTAACFDRWDLPLSDMYTPYVFPSENGLRCGTRELNYGPHQWRGDFQFNISRYSQQQLMETSHRHLLHAEEGT<br>WLNIDGFHMGIGGDDSWSPSVSAEFQLSAGRYHYQLVWCQKYPYDVPDYA-
	β-gal: black; amino acid point of insertion: red; N8 yellow; linker: gray
β-gal_D223_5gs:N8:5gs	MVLQRRDWENPGVTQLNRLAAHPPFASWRNSEEARTDRPSQQLRSLNGEWRFAWFPAPEAVPESWLECDLPEADTVVVPSNWQMHGYDAPIYTNVTYPIT<br>VNPPFVPTENPTGCYSLTFNVDESWLQEGQTRIIFDGVNSAFHLWCNGRWVGYGQDSRLPSEFDLSAFLRAGENRLAVMVLRWSDGSYLEDQDMWRMSGI<br>FRDVSLLHKPTTQISDFHVATRFNDGSSGSYGKIAALKAENAALEAKIAALKAEIAALEAGGSGGSDFSRAVLEAEVQMCGELRDYLRVTVSLWQGETQVASG<br>TAPFGGEIIDERGGYADRVTLRLNVENPKLWSAEIPNLYRAVVELHTADGTLIEAEACDVGFREVRIENGLLLLNGKPLLIRGVNRHEHHPLHGQVMDEQTMVQ<br>DILLMKQNNFNAVRCSHYPNHPLWYTLCDRYGLYVVDEANIETHGMVPMNRLTDDPRWLPAMSERVTRMVQRDRNHPSVIIWSLGNESGHGANHDALYRWI<br>KSVDPSRPVQYEGGGADTTATDIICPMYARVDEDQPFPAVPKWSIKKWLSLPGETRPLILCEYAHAMGNSLGGFAKYWQAFRQYPRLQGGFVWDWVDQSLI<br>KYDENGNPWSAYGGDFGDTPNDRQFCMNGLVFADRTPHPALTEAKHQQQFFQFRLSGQTIEVTSEYLFRHSDNELLHWMVALDGKPLASGEVPLDVAPQG<br>KQLIELPELPQPESAGQLWLTVRVVQPNATAWSEAGHISAWQQWRLAENLSVTLPAASHAIPHLTTSEMDFCIELGNKRWQFNRQSGFLSQMWIGDKKQLLT<br>PLRDQFTRAPLDNDIGVSEATRIDPNAWVERWKAAGHYQAEAALLQCTADTLADAVLITTAHAWQHQGKTLFISRKTYRIDGSGQMAITVDVEVASDTPHPARI<br>GLNCQLAQVAERVNWLGLGPQENYPDRLTAACFDRWDLPLSDMYTPYVFPSENGLRCGTRELNYGPHQWRGDFQFNISRYSQQQLMETSHRHLLHAEEGT<br>WLNIDGFHMGIGGDDSWSPSVSAEFQLSAGRYHYQLVWCQKYPYDVPDYA-
	β-gal: black; amino acid point of insertion: red; N8 yellow; linker: gray
β-gal_A229_5gs:N8:5gs	MVLQRRDWENPGVTQLNRLAAHPPFASWRNSEEARTDRPSQQLRSLNGEWRFAWFPAPEAVPESWLECDLPEADTVVVPSNWQMHGYDAPIYTNVTYPIT<br>VNPPFVPTENPTGCYSLTFNVDESWLQEGQTRIIFDGVNSAFHLWCNGRWVGYGQDSRLPSEFDLSAFLRAGENRLAVMVLRWSDGSYLEDQDMWRMSGI<br>FRDVSLLHKPTTQISDFHVAGSSGSYGKIAALKAENAALEAKIAALKAEIAALEAGGSGGSTRFNDDFSRAVLEAEVQMCGELRDYLRVTVSLWQGETQVASG<br>TAPFGGEIIDERGGYADRVTLRLNVENPKLWSAEIPNLYRAVVELHTADGTLIEAEACDVGFREVRIENGLLLLNGKPLLIRGVNRHEHHPLHGQVMDEQTMVQ<br>DILLMKQNNFNAVRCSHYPNHPLWYTLCDRYGLYVVDEANIETHGMVPMNRLTDDPRWLPAMSERVTRMVQRDRNHPSVIIWSLGNESGHGANHDALYRWI<br>KSVDPSRPVQYEGGGADTTATDIICPMYARVDEDQPFPAVPKWSIKKWLSLPGETRPLILCEYAHAMGNSLGGFAKYWQAFRQYPRLQGGFVWDWVDQSLI<br>KYDENGNPWSAYGGDFGDTPNDRQFCMNGLVFADRTPHPALTEAKHQQQFFQFRLSGQTIEVTSEYLFRHSDNELLHWMVALDGKPLASGEVPLDVAPQG<br>KQLIELPELPQPESAGQLWLTVRVVQPNATAWSEAGHISAWQQWRLAENLSVTLPAASHAIPHLTTSEMDFCIELGNKRWQFNRQSGFLSQMWIGDKKQLLT<br>PLRDQFTRAPLDNDIGVSEATRIDPNAWVERWKAAGHYQAEAALLQCTADTLADAVLITTAHAWQHQGKTLFISRKTYRIDGSGQMAITVDVEVASDTPHPARI<br>GLNCQLAQVAERVNWLGLGPQENYPDRLTAACFDRWDLPLSDMYTPYVFPSENGLRCGTRELNYGPHQWRGDFQFNISRYSQQQLMETSHRHLLHAEEGT<br>WLNIDGFHMGIGGDDSWSPSVSAEFQLSAGRYHYQLVWCQKYPYDVPDYA-
	β-gal: black; amino acid point of insertion: red; N8 yellow; linker: gray

β-gal_A239_5gs: N8:5gs	MVLQRRDWENPGVTQLNRLAAHPPFASWRNSEEARTDRPSQQLRSLNGEWRFAWFPAPEAVPESWLECDLPEADTVVVPSNWQMHGYDAPIYTNVTYPIT<br>VNPPFVPTENPTGCYSLTFNVDESWLQEGQTRIIFDGVNSAFHLWCNGRWVGYGQDSRLPSEFDLSAFLRAGENRLAVMVLRWSDGSYLEDQDMWRMSGI<br>FRDVSLLHKPTTQISDFHVATRFNDDFSRAGSSGSYGKIAALKAENAALEAKIAALKAEIAALEAGGSGGSVLEAEVQMCGELRDYLRVTVSLWQGETQVASG<br>TAPFGGEIIDERGGYADRVTLRLNVENPKLWSAEIPNLYRAVVELHTADGTLIEAEACDVGFREVRIENGLLLLNGKPLLIRGVNRHEHHPLHGQVMDEQTMVQ<br>DILLMKQNNFNAVRCSHYPNHPLWYTLCDRYGLYVVDEANIETHGMVPMNRLTDDPRWLPAMSERVTRMVQRDRNHPSVIIWSLGNESGHGANHDALYRWI<br>KSVDPSRPVQYEGGGADTTATDIICPMYARVDEDQPFPAVPKWSIKKWLSLPGETRPLILCEYAHAMGNSLGGFAKYWQAFRQYPRLQGGFVWDWVDQSLI<br>KYDENGNPWSAYGGDFGDTPNDRQFCMNGLVFADRTPHPALTEAKHQQQFFQFRLSGQTIEVTSEYLFRHSDNELLHWMVALDGKPLASGEVPLDVAPQG<br>KQLIELPELPQPESAGQLWLTVRVVQPNATAWSEAGHISAWQQWRLAENLSVTLPAASHAIPHLTTSEMDFCIELGNKRWQFNRQSGFLSQMWIGDKKQLLT<br>PLRDQFTRAPLDNDIGVSEATRIDPNAWVERWKAAGHYQAEAALLQCTADTLADAVLITTAHAWQHQGKTLFISRKTYRIDGSGQMAITVDVEVASDTPHPARI<br>GLNCQLAQVAERVNWLGLGPQENYPDRLTAACFDRWDLPLSDMYTPYVFPSENGLRCGTRELNYGPHQWRGDFQFNISRYSQQQLMETSHRHLLHAEEGT<br>WLNIDGFHMGIGGDDSWSPSVSAEFQLSAGRYHYQLVWCQKYPYDVPDYA-
	β-gal: black; amino acid point of insertion: red; N8 yellow; linker: gray
β-gal_D507_5gs: N8:5gs	MVLQRRDWENPGVTQLNRLAAHPPFASWRNSEEARTDRPSQQLRSLNGEWRFAWFPAPEAVPESWLECDLPEADTVVVPSNWQMHGYDAPIYTNVTYPIT<br>VNPPFVPTENPTGCYSLTFNVDESWLQEGQTRIIFDGVNSAFHLWCNGRWVGYGQDSRLPSEFDLSAFLRAGENRLAVMVLRWSDGSYLEDQDMWRMSGI<br>FRDVSLLHKPTTQISDFHVATRFNDDFSRAVLEAEVQMCGELRDYLRVTVSLWQGETQVASGTAPFGGEIIDERGGYADRVTLRLNVENPKLWSAEIPNLYRA<br>VVELHTADGTLIEAEACDVGFREVRIENGLLLLNGKPLLIRGVNRHEHHPLHGQVMDEQTMVQDILLMKQNNFNAVRCSHYPNHPLWYTLCDRYGLYVVDEAN<br>IETHGMVPMNRLTDDPRWLPAMSERVTRMVQRDRNHPSVIIWSLGNESGHGANHDALYRWIKSVDPSRPVQYEGGGADTTATDIICPMYARVDGSSGSYGK<br>IAALKAENAALEAKIAALKAEIAALEAGGSGGSEDQPFPAVPKWSIKKWLSLPGETRPLILCEYAHAMGNSLGGFAKYWQAFRQYPRLQGGFVWDWVDQSLIK<br>YDENGNPWSAYGGDFGDTPNDRQFCMNGLVFADRTPHPALTEAKHQQQFFQFRLSGQTIEVTSEYLFRHSDNELLHWMVALDGKPLASGEVPLDVAPQGK<br>QLIELPELPQPESAGQLWLTVRVVQPNATAWSEAGHISAWQQWRLAENLSVTLPAASHAIPHLTTSEMDFCIELGNKRWQFNRQSGFLSQMWIGDKKQLLTP<br>LRDQFTRAPLDNDIGVSEATRIDPNAWVERWKAAGHYQAEAALLQCTADTLADAVLITTAHAWQHQGKTLFISRKTYRIDGSGQMAITVDVEVASDTPHPARIG<br>LNCQLAQVAERVNWLGLGPQENYPDRLTAACFDRWDLPLSDMYTPYVFPSENGLRCGTRELNYGPHQWRGDFQFNISRYSQQQLMETSHRHLLHAEEGT<br>WLNIDGFHMGIGGDDSWSPSVSAEFQLSAGRYHYQLVWCQKYPYDVPDYA-
	β-gal: black; amino acid point of insertion: red; N8 yellow; linker: gray
β-gal_P513_5gs: N8:5gs	MVLQRRDWENPGVTQLNRLAAHPPFASWRNSEEARTDRPSQQLRSLNGEWRFAWFPAPEAVPESWLECDLPEADTVVVPSNWQMHGYDAPIYTNVTYPIT<br>VNPPFVPTENPTGCYSLTFNVDESWLQEGQTRIIFDGVNSAFHLWCNGRWVGYGQDSRLPSEFDLSAFLRAGENRLAVMVLRWSDGSYLEDQDMWRMSGI<br>FRDVSLLHKPTTQISDFHVATRFNDDFSRAVLEAEVQMCGELRDYLRVTVSLWQGETQVASGTAPFGGEIIDERGGYADRVTLRLNVENPKLWSAEIPNLYRA<br>VVELHTADGTLIEAEACDVGFREVRIENGLLLLNGKPLLIRGVNRHEHHPLHGQVMDEQTMVQDILLMKQNNFNAVRCSHYPNHPLWYTLCDRYGLYVVDEAN<br>IETHGMVPMNRLTDDPRWLPAMSERVTRMVQRDRNHPSVIIWSLGNESGHGANHDALYRWIKSVDPSRPVQYEGGGADTTATDIICPMYARVDEDQPFPGS<br>SGSYGKIAALKAENAALEAKIAALKAEIAALEAGGSGGSAVPKWSIKKWLSLPGETRPLILCEYAHAMGNSLGGFAKYWQAFRQYPRLQGGFVWDWVDQSLIK<br>YDENGNPWSAYGGDFGDTPNDRQFCMNGLVFADRTPHPALTEAKHQQQFFQFRLSGQTIEVTSEYLFRHSDNELLHWMVALDGKPLASGEVPLDVAPQGK<br>QLIELPELPQPESAGQLWLTVRVVQPNATAWSEAGHISAWQQWRLAENLSVTLPAASHAIPHLTTSEMDFCIELGNKRWQFNRQSGFLSQMWIGDKKQLLTP<br>LRDQFTRAPLDNDIGVSEATRIDPNAWVERWKAAGHYQAEAALLQCTADTLADAVLITTAHAWQHQGKTLFISRKTYRIDGSGQMAITVDVEVASDTPHPARIG<br>LNCQLAQVAERVNWLGLGPQENYPDRLTAACFDRWDLPLSDMYTPYVFPSENGLRCGTRELNYGPHQWRGDFQFNISRYSQQQLMETSHRHLLHAEEGT<br>WLNIDGFHMGIGGDDSWSPSVSAEFQLSAGRYHYQLVWCQKYPYDVPDYA-
	β-gal: black; amino acid point of insertion: red; N8 yellow; linker: gray
β-gal_D610_5gs: N8:5gs	MVLQRRDWENPGVTQLNRLAAHPPFASWRNSEEARTDRPSQQLRSLNGEWRFAWFPAPEAVPESWLECDLPEADTVVVPSNWQMHGYDAPIYTNVTYPIT<br>VNPPFVPTENPTGCYSLTFNVDESWLQEGQTRIIFDGVNSAFHLWCNGRWVGYGQDSRLPSEFDLSAFLRAGENRLAVMVLRWSDGSYLEDQDMWRMSGI<br>FRDVSLLHKPTTQISDFHVATRFNDDFSRAVLEAEVQMCGELRDYLRVTVSLWQGETQVASGTAPFGGEIIDERGGYADRVTLRLNVENPKLWSAEIPNLYRA<br>VVELHTADGTLIEAEACDVGFREVRIENGLLLLNGKPLLIRGVNRHEHHPLHGQVMDEQTMVQDILLMKQNNFNAVRCSHYPNHPLWYTLCDRYGLYVVDEAN<br>IETHGMVPMNRLTDDPRWLPAMSERVTRMVQRDRNHPSVIIWSLGNESGHGANHDALYRWIKSVDPSRPVQYEGGGADTTATDIICPMYARVDEDQPFPAV<br>PKWSIKKWLSLPGETRPLILCEYAHAMGNSLGGFAKYWQAFRQYPRLQGGFVWDWVDQSLIKYDENGNPWSAYGGDFGDTPNDRQFCMNGLVFADGSSG<br>SYGKIAALKAENAALEAKIAALKAEIAALEAGGSGGSRTPHPALTEAKHQQQFFQFRLSGQTIEVTSEYLFRHSDNELLHWMVALDGKPLASGEVPLDVAPQGK<br>QLIELPELPQPESAGQLWLTVRVVQPNATAWSEAGHISAWQQWRLAENLSVTLPAASHAIPHLTTSEMDFCIELGNKRWQFNRQSGFLSQMWIGDKKQLLTP<br>LRDQFTRAPLDNDIGVSEATRIDPNAWVERWKAAGHYQAEAALLQCTADTLADAVLITTAHAWQHQGKTLFISRKTYRIDGSGQMAITVDVEVASDTPHPARIG<br>LNCQLAQVAERVNWLGLGPQENYPDRLTAACFDRWDLPLSDMYTPYVFPSENGLRCGTRELNYGPHQWRGDFQFNISRYSQQQLMETSHRHLLHAEEGT<br>WLNIDGFHMGIGGDDSWSPSVSAEFQLSAGRYHYQLVWCQKYPYDVPDYA-
	β-gal: black; amino acid point of insertion: red; N8 yellow; linker: gray
β-gal_T799_5gs: N8:5gs	MVLQRRDWENPGVTQLNRLAAHPPFASWRNSEEARTDRPSQQLRSLNGEWRFAWFPAPEAVPESWLECDLPEADTVVVPSNWQMHGYDAPIYTNVTYPIT<br>VNPPFVPTENPTGCYSLTFNVDESWLQEGQTRIIFDGVNSAFHLWCNGRWVGYGQDSRLPSEFDLSAFLRAGENRLAVMVLRWSDGSYLEDQDMWRMSGI<br>FRDVSLLHKPTTQISDFHVATRFNDDFSRAVLEAEVQMCGELRDYLRVTVSLWQGETQVASGTAPFGGEIIDERGGYADRVTLRLNVENPKLWSAEIPNLYRA<br>VVELHTADGTLIEAEACDVGFREVRIENGLLLLNGKPLLIRGVNRHEHHPLHGQVMDEQTMVQDILLMKQNNFNAVRCSHYPNHPLWYTLCDRYGLYVVDEAN<br>IETHGMVPMNRLTDDPRWLPAMSERVTRMVQRDRNHPSVIIWSLGNESGHGANHDALYRWIKSVDPSRPVQYEGGGADTTATDIICPMYARVDEDQPFPAV<br>PKWSIKKWLSLPGETRPLILCEYAHAMGNSLGGFAKYWQAFRQYPRLQGGFVWDWVDQSLIKYDENGNPWSAYGGDFGDTPNDRQFCMNGLVFADRTPH<br>PALTEAKHQQQFFQFRLSGQTIEVTSEYLFRHSDNELLHWMVALDGKPLASGEVPLDVAPQGKQLIELPELPQPESAGQLWLTVRVVQPNATAWSEAGHISA<br>WQQWRLAENLSVTLPAASHAIPHLTTSEMDFCIELGNKRWQFNRQSGFLSQMWIGDKKQLLTPLRDQFTRAPLDNDIGVSEATGSSGSYGKIAALKAENAALE<br>AKIAALKAEIAALEAGGSGGSRIDPNAWVERWKAAGHYQAEAALLQCTADTLADAVLITTAHAWQHQGKTLFISRKTYRIDGSGQMAITVDVEVASDTPHPARI<br>GLNCQLAQVAERVNWLGLGPQENYPDRLTAACFDRWDLPLSDMYTPYVFPSENGLRCGTRELNYGPHQWRGDFQFNISRYSQQQLMETSHRHLLHAEEGT<br>WLNIDGFHMGIGGDDSWSPSVSAEFQLSAGRYHYQLVWCQKYPYDVPDYA-
	β-gal: black; amino acid point of insertion: red; N8 yellow; linker: gray
β-gal_S1000_5gs: N8:5gs	MVLQRRDWENPGVTQLNRLAAHPPFASWRNSEEARTDRPSQQLRSLNGEWRFAWFPAPEAVPESWLECDLPEADTVVVPSNWQMHGYDAPIYTNVTYPIT<br>VNPPFVPTENPTGCYSLTFNVDESWLQEGQTRIIFDGVNSAFHLWCNGRWVGYGQDSRLPSEFDLSAFLRAGENRLAVMVLRWSDGSYLEDQDMWRMSGI<br>FRDVSLLHKPTTQISDFHVATRFNDDFSRAVLEAEVQMCGELRDYLRVTVSLWQGETQVASGTAPFGGEIIDERGGYADRVTLRLNVENPKLWSAEIPNLYRA<br>VVELHTADGTLIEAEACDVGFREVRIENGLLLLNGKPLLIRGVNRHEHHPLHGQVMDEQTMVQDILLMKQNNFNAVRCSHYPNHPLWYTLCDRYGLYVVDEAN<br>IETHGMVPMNRLTDDPRWLPAMSERVTRMVQRDRNHPSVIIWSLGNESGHGANHDALYRWIKSVDPSRPVQYEGGGADTTATDIICPMYARVDEDQPFPAV<br>PKWSIKKWLSLPGETRPLILCEYAHAMGNSLGGFAKYWQAFRQYPRLQGGFVWDWVDQSLIKYDENGNPWSAYGGDFGDTPNDRQFCMNGLVFADRTPH<br>PALTEAKHQQQFFQFRLSGQTIEVTSEYLFRHSDNELLHWMVALDGKPLASGEVPLDVAPQGKQLIELPELPQPESAGQLWLTVRVVQPNATAWSEAGHISA<br>WQQWRLAENLSVTLPAASHAIPHLTTSEMDFCIELGNKRWQFNRQSGFLSQMWIGDKKQLLTPLRDQFTRAPLDNDIGVSEATRIDPNAWVERWKAAGHYQ<br>AEAALLQCTADTLADAVLITTAHAWQHQGKTLFISRKTYRIDGSGQMAITVDVEVASDTPHPARIGLNCQLAQVAERVNWLGLGPQENYPDRLTAACFDRWDL<br>PLSDMYTPYVFPSENGLRCGTRELNYGPHQWRGDFQFNISRYSQQQLMETSHRHLLHAEEGTWLNIDGFHMGIGGDDSWSGSSGSYGKIAALKAENAALEA<br>KIAALKAEIAALEAGGSGGSPSVSAEFQLSAGRYHYQLVWCQKYPYDVPDYA-
	β-gal: black; amino acid point of insertion: red; N8 yellow; linker: gray
mMyD88_T66_5gs:N8:5gs	MDTYRYISAGDPRVGSGSLDSFMFSIPLVALNVGVRRRLSLFLNPRTPVAADWTLLAEEMGFEYLEIRELETGSSGSYGKIAALKAENAALEAKIAALKAEIAALE<br>AGGSGGSRPDPTRSLLDAWQGRSGASVGRLLELLALLDREDILKELKSRIEEDCQKYLGKQQNQESEKPLQVARVESSVPQTKELGGITTLDDPLGQTPELFD<br>AFICYCPNDIEFVQEMIRQLEQTDYRLKLCVSDRDVLPGTCVWSIASELIEKRCRRMVVVVSDDYLQSKECDFQTKFALSLSPGVQQKRLIPIKYKAMKKDFPSI<br>LRFITICDYTNPCTKSWFWTRLAKALSLP-

	mMyD88: black; amino acid point of insertion: red; N8 yellow; linker: gray
mMyD88_G80_5gs:N8:5gs	MDTYRYISAGDPRVGSGSLDSFMFSIPLVALNVGVRRRLSLFLNPRTPVAADWTLLAEEMGFEYLEIRELETRPDPTRSLLDAWQGGSSGSYGKIAALKAENAALEAKIAALKAEIAALEAGGSGGSRSGASVGRLLELLALLDREDILKELKSRIEEDCQKYLGKQQNQESEKPLQVARVESSVPQTKELGGITTLDDPLGQTPELFDAFICYCPNDIEFVQEMIRQLEQTDYRLKLCVSDRDVLPGTCVWSIASELIEKRCRRMVVVVSDDYLQSKECDFQTKFALSLSPGVQQKRLIPIKYKAMKKDFPSILRFITICDYTNPCTKSWFWTRLAKALSLP-
	mMyD88: black; amino acid point of insertion: red; N8 yellow; linker: gray
mMyD88_N170_5gs:N8:5gs	MDTYRYISAGDPRVGSGSLDSFMFSIPLVALNVGVRRRLSLFLNPRTPVAADWTLLAEEMGFEYLEIRELETRPDPTRSLLDAWQGRSGASVGRLLELLALLDREDILKELKSRIEEDCQKYLGKQQNQESEKPLQVARVESSVPQTKELGGITTLDDPLGQTPELFDAFICYCPNGSSGSYGKIAALKAENAALEAKIAALKAEIAALEAGGSGGSDIEFVQEMIRQLEQTDYRLKLCVSDRDVLPGTCVWSIASELIEKRCRRMVVVVSDDYLQSKECDFQTKFALSLSPGVQQKRLIPIKYKAMKKDFPSILRFITICDYTNPCTKSWFWTRLAKALSLP-
	mMyD88: black; amino acid point of insertion: red; N8 yellow; linker: gray
mMyD88_S209_5gs:N8:5gs	MDTYRYISAGDPRVGSGSLDSFMFSIPLVALNVGVRRRLSLFLNPRTPVAADWTLLAEEMGFEYLEIRELETRPDPTRSLLDAWQGRSGASVGRLLELLALLDREDILKELKSRIEEDCQKYLGKQQNQESEKPLQVARVESSVPQTKELGGITTLDDPLGQTPELFDAFICYCPNDIEFVQEMIRQLEQTDYRLKLCVSDRDVLPGTCVWSIASGSSGSYGKIAALKAENAALEAKIAALKAEIAALEAGGSGGSELIEKRCRRMVVVVSDDYLQSKECDFQTKFALSLSPGVQQKRLIPIKYKAMKKDFPSILRFITICDYTNPCTKSWFWTRLAKALSLP-
	mMyD88: black; amino acid point of insertion: red; N8 yellow; linker: gray
mMyD88_S224_5gs:N8:5gs	MDTYRYISAGDPRVGSGSLDSFMFSIPLVALNVGVRRRLSLFLNPRTPVAADWTLLAEEMGFEYLEIRELETRPDPTRSLLDAWQGRSGASVGRLLELLALLDREDILKELKSRIEEDCQKYLGKQQNQESEKPLQVARVESSVPQTKELGGITTLDDPLGQTPELFDAFICYCPNDIEFVQEMIRQLEQTDYRLKLCVSDRDVLPGTCVWSIASELIEKRCRRMVVVVSGSSGSYGKIAALKAENAALEAKIAALKAEIAALEAGGSGGSDDYLQSKECDFQTKFALSLSPGVQQKRLIPIKYKAMKKDFPSILRFITICDYTNPCTKSWFWTRLAKALSLP-
	mMyD88: black; amino acid point of insertion: red; N8 yellow; linker: gray
mIRAK1_F212_5gs:N8:5gs	MAGGPGPGEPVVPGAQHFLYEVPPWVMCRFYKVMDALEPADWCQFAALIVRDQTELRLCERSEQRTASVLWPWINRNARVADLVHILTHLQLLRARDIITAWHPPAPVVPPSTAAPRPSSISAGSEAGDWSPRKLQSSASTFLSPAFPGSQTHSESELLQVPLPVSLGPPLPSSAPSSTKSSPESPVSGLQRAHPSPFCWPFCEISQGTCNFSPGGSKIAALKAENAALEAKIAALKAEIAALEAGYGGSGGGSEELRIGEGGFGCVYRAVMRNTTYAVKRLKEEADLEWTMVKQSFLTEVEQLSRFRHPNIVDFAGYCAESGLYCLVYGFLPNGSLEDQLHLQTQACSPLSWPQRLDILLGTARAIQFLHQDSPSLIHGDIKSSNVLLDERLMPKLGDFGLARFSRFAGAKASQSSTVARTSTVRGTLAYLPEEYIKTGRLAVDTDTFSFGVVILETLAGQRAVRTQGAKTKYLKDLIEDEAEEAGVTLKSTQPTLWVGVATDAWAAPIAAQIYKKHLDSRPGPCPPQLGLALAQLACCCMHRRAKKRPPMTQVYKRLEGLQAGPPWELEVAGHGSPSPQENSYMSTTGSAQSGDEPWQPLVVTTRAPAQAAQQLQRSPNQPVESDESVPGLSATLHSWHLTPGSHPSPASFREASCTQGGTTRESSVRSSPGFQPTTMEGSPTGSSSLLSSEPPQIIINPARQKMVQKLALYEEGVLDSLQLLSSGFFPGLDLEPEKSQGPEESDEFQSYPYDVPDYA-
	mIRAK1: black; amino acid point of insertion: red; N8 yellow; linker: gray
mIRAK1_G221_5gs:N8:5gs	MAGGPGPGEPVVPGAQHFLYEVPPWVMCRFYKVMDALEPADWCQFAALIVRDQTELRLCERSEQRTASVLWPWINRNARVADLVHILTHLQLLRARDIITAWHPPAPVVPPSTAAPRPSSISAGSEAGDWSPRKLQSSASTFLSPAFPGSQTHSESELLQVPLPVSLGPPLPSSAPSSTKSSPESPVSGLQRAHPSPFCWPFCEISQGTCNFSEELRIGEGSPGGSKIAALKAENAALEAKIAALKAEIAALEAGYGGSGGGGFGCVYRAVMRNTTYAVKRLKEEADLEWTMVKQSFLTEVEQLSRFRHPNIVDFAGYCAESGLYCLVYGFLPNGSLEDQLHLQTQACSPLSWPQRLDILLGTARAIQFLHQDSPSLIHGDIKSSNVLLDERLMPKLGDFGLARFSRFAGAKASQSSTVARTSTVRGTLAYLPEEYIKTGRLAVDTDTFSFGVVILETLAGQRAVRTQGAKTKYLKDLIEDEAEEAGVTLKSTQPTLWVGVATDAWAAPIAAQIYKKHLDSRPGPCPPQLGLALAQLACCCMHRRAKKRPPMTQVYKRLEGLQAGPPWELEVAGHGSPSPQENSYMSTTGSAQSGDEPWQPLVVTTRAPAQAAQQLQRSPNQPVESDESVPGLSATLHSWHLTPGSHPSPASFREASCTQGGTTRESSVRSSPGFQPTTMEGSPTGSSSLLSSEPPQIIINPARQKMVQKLALYEEGVLDSLQLLSSGFFPGLDLEPEKSQGPEESDEFQSYPYDVPDYA-
	mIRAK1: black; amino acid point of insertion: red; N8 yellow; linker: gray
mIRAK1_R232_5gs:N8:5gs	MAGGPGPGEPVVPGAQHFLYEVPPWVMCRFYKVMDALEPADWCQFAALIVRDQTELRLCERSEQRTASVLWPWINRNARVADLVHILTHLQLLRARDIITAWHPPAPVVPPSTAAPRPSSISAGSEAGDWSPRKLQSSASTFLSPAFPGSQTHSESELLQVPLPVSLGPPLPSSAPSSTKSSPESPVSGLQRAHPSPFCWPFCEISQGTCNFSEELRIGEGGFGCVYRAVMRSPGGSKIAALKAENAALEAKIAALKAEIAALEAGYGGSGGGNTTYAVKRLKEEADLEWTMVKQSFLTEVEQLSRFRHPNIVDFAGYCAESGLYCLVYGFLPNGSLEDQLHLQTQACSPLSWPQRLDILLGTARAIQFLHQDSPSLIHGDIKSSNVLLDERLMPKLGDFGLARFSRFAGAKASQSSTVARTSTVRGTLAYLPEEYIKTGRLAVDTDTFSFGVVILETLAGQRAVRTQGAKTKYLKDLIEDEAEEAGVTLKSTQPTLWVGVATDAWAAPIAAQIYKKHLDSRPGPCPPQLGLALAQLACCCMHRRAKKRPPMTQVYKRLEGLQAGPPWELEVAGHGSPSPQENSYMSTTGSAQSGDEPWQPLVVTTRAPAQAAQQLQRSPNQPVESDESVPGLSATLHSWHLTPGSHPSPASFREASCTQGGTTRESSVRSSPGFQPTTMEGSPTGSSSLLSSEPPQIIINPARQKMVQKLALYEEGVLDSLQLLSSGFFPGLDLEPEKSQGPEESDEFQSYPYDVPDYA-
	mIRAK1: black; amino acid point of insertion: red; N8 yellow; linker: gray
mIRAK1_S281_5gs:N8:5gs	MAGGPGPGEPVVPGAQHFLYEVPPWVMCRFYKVMDALEPADWCQFAALIVRDQTELRLCERSEQRTASVLWPWINRNARVADLVHILTHLQLLRARDIITAWHPPAPVVPPSTAAPRPSSISAGSEAGDWSPRKLQSSASTFLSPAFPGSQTHSESELLQVPLPVSLGPPLPSSAPSSTKSSPESPVSGLQRAHPSPFCWPFCEISQGTCNFSEELRIGEGGFGCVYRAVMRNTTYAVKRLKEEADLEWTMVKQSFLTEVEQLSRFRHPNIVDFAGYCAESSPGGSKIAALKAENAALEAKIAALKAEIAALEAGYGGSGGGLYCLVYGFLPNGSLEDQLHLQTQACSPLSWPQRLDILLGTARAIQFLHQDSPSLIHGDIKSSNVLLDERLMPKLGDFGLARFSRFAGAKASQSSTVARTSTVRGTLAYLPEEYIKTGRLAVDTDTFSFGVVILETLAGQRAVRTQGAKTKYLKDLIEDEAEEAGVTLKSTQPTLWVGVATDAWAAPIAAQIYKKHLDSRPGPCPPQLGLALAQLACCCMHRRAKKRPPMTQVYKRLEGLQAGPPWELEVAGHGSPSPQENSYMSTTGSAQSGDEPWQPLVVTTRAPAQAAQQLQRSPNQPVESDESVPGLSATLHSWHLTPGSHPSPASFREASCTQGGTTRESSVRSSPGFQPTTMEGSPTGSSSLLSSEPPQIIINPARQKMVQKLALYEEGVLDSLQLLSSGFFPGLDLEPEKSQGPEESDEFQSYPYDVPDYA-
	mIRAK1: black; amino acid point of insertion: red; N8 yellow; linker: gray
mCherry:N7	MEQKLISEEDLMVSKGEEDNMAIIKEFMRFKVHMEGSVNGHEFEIEGEGEGRPYEGTQTAKLKVTKGGPLPFAWDILSPQFMYGSKAYVKHPADIPDYLKLSFPEGFKWERVMNFEDGGVVTVTQDSSLQDGEFIYKVKLRGTNFPSDGPVMQKKTMGWEASSERMYPEDGALKGEIKQRLKLKDGGHYDAEVKTTYKAKKPVQLPGAYNVNIKLDITSHNEDYTIVEQYERAEGRHSTGGMDELYKGGSGGGSGGSGEIAALEAKNAALKAEIAALEAKIAALKAGY*
	mCherry: black; N7: dark blue; linker: gray; myc tag:brown
mCherry:P7A	MEQKLISEEDLMVSKGEEDNMAIIKEFMRFKVHMEGSVNGHEFEIEGEGEGRPYEGTQTAKLKVTKGGPLPFAWDILSPQFMYGSKAYVKHPADIPDYLKLSFPEGFKWERVMNFEDGGVVTVTQDSSLQDGEFIYKVKLRGTNFPSDGPVMQKKTMGWEASSERMYPEDGALKGEIKQRLKLKDGGHYDAEVKTTYKAKKPVQLPGAYNVNIKLDITSHNEDYTIVEQYERAEGRHSTGGMDELYKGGSGGGSGGSGEIAALEAKNAALKAEIAALEAKNAALKAGC*
	mCherry: black; P7A: cyan; linker: gray; myc tag:brown
mCherry:P7	MEQKLISEEDLMVSKGEEDNMAIIKEFMRFKVHMEGSVNGHEFEIEGEGEGRPYEGTQTAKLKVTKGGPLPFAWDILSPQFMYGSKAYVKHPADIPDYLKLSFPEGFKWERVMNFEDGGVVTVTQDSSLQDGEFIYKVKLRGTNFPSDGPVMQKKTMGWEASSERMYPEDGALKGEIKQRLKLKDGGHYDAEVKTTYKAKKPVQLPGAYNVNIKLDITSHNEDYTIVEQYERAEGRHSTGGMDELYKGGSGGGSGGSEIQALEEKNAQLKQEIAALEEKNQALKYG*

	mCherry: black; P7: green; linker: gray; myc tag:brown
mCherry:P7SN	MEQKLISEEDLMVSKGEEDNMAIIKEFMRFKVHMEGSVNGHEFEIEGEGEGRPYEGTQTAKLKVTKGGPLPFAWDILSPQFMYGSKAYVKHPADIPDYLKLSFPEGFKWERVMNFEDGGVVTVTQDSSLQDGEFIYKVKLRGTNFPSDGPVMQKKTMGWEASSERMYPEDGALKGEIKQRLKLKDGGHYDAEVKTTYKAKKPVQLPGAYNVNIKLDITSHNEDYTIVEQYERAEGRHSTGGMDELYKGGSGGGSGGSEIQQLEEKNSQLKQEISQLEEKNQELKYG*
	mCherry: black; P7SN:pink; linker: gray; myc tag:brown
mCherry:N8	MEQKLISEEDLMVSKGEEDNMAIIKEFMRFKVHMEGSVNGHEFEIEGEGEGRPYEGTQTAKLKVTKGGPLPFAWDILSPQFMYGSKAYVKHPADIPDYLKLSFPEGFKWERVMNFEDGGVVTVTQDSSLQDGEFIYKVKLRGTNFPSDGPVMQKKTMGWEASSERMYPEDGALKGEIKQRLKLKDGGHYDAEVKTTYKAKKPVQLPGAYNVNIKLDITSHNEDYTIVEQYERAEGRHSTGGMDELYKGGSGGGSGGSYGKIAALKAENAALEAKIAALKAEIAALEAGY*
	mCherry: black; N8:yellow; linker: gray; myc tag:brown
ngGFP_C143_5gs:P7:5gs	MHHHHHHHHMVSKGEEDNMASLPATHELHIFGSINGVDFDMVGQGTGNPNDGYEELNLKSTKGDLQFSPWILVPHIGYGFHQYLPYPDGMSPFQAAMVDGSGYQVHRTMQFEDGASLTVNYRYTYEGSHIKGEAQVKGTGFPADGPVMTNCGSSGSEIQALEEKNAQLKQEIAALEEKNQALKYGGSGGSLTAADWSRSKKTYPNDKTIISTFKWSYTTGNGKRYRSTARTTYTFAKPMAANYLKNQPMYVFRKTELKHSKTELNFKEWQKAFTDVMGMDELYK*
	ngGFP: black; amino acid point of insertion: red; P7: green; linker: gray; his tag: dark blue
ngGFP_C143_5gs:P7:5gs_gs:N8	MHHHHHHHHMVSKGEEDNMASLPATHELHIFGSINGVDFDMVGQGTGNPNDGYEELNLKSTKGDLQFSPWILVPHIGYGFHQYLPYPDGMSPFQAAMVDGSGYQVHRTMQFEDGASLTVNYRYTYEGSHIKGEAQVKGTGFPADGPVMTNCGSSGSEIQALEEKNAQLKQEIAALEEKNQALKYGGSGGSLTAADWSRSKKTYPNDKTIISTFKWSYTTGNGKRYRSTARTTYTFAKPMAANYLKNQPMYVFRKTELKHSKTELNFKEWQKAFTDVMGMDELYKGSPGGSPGGSGSPGGSPGGSKIAALKAENAALEAKIAALKAEIAALEAGY*
	ngGFP: black; amino acid point of insertion: red; P7: green; linker: gray; his tag: dark blue; N8:yellow
GFP_K214_5gs:P7:5gs	MSKGEELFTGVVPILVELDGDVNGHKFSVRGEGEGDATIGKLTLKFICTTGKLPVPWPTLVTTLTYGVQCFSRYPDHMKRHDFFKSAMPEGYVQERTISFKDDGKYKTRAVVKFEGDTLVNRIELKGTDFKEDGNILGHKLEYNFNSHNVYITADKQKNGIKANFTVRHNVEDGSVQLADHYQQNTPIGDGPVLLPDNHYLSTQTVLSKDPNEKGSSGSEIQALEEKNAQLKQEIAALEEKNQALKYGGSGGSRDHMVLHEYVNAAGIT*
	ngGFP: black; amino acid point of insertion: red; P7: green; linker: gray;
GFP_K214_5gs:P7:5gs:N8	MSKGEELFTGVVPILVELDGDVNGHKFSVRGEGEGDATIGKLTLKFICTTGKLPVPWPTLVTTLTYGVQCFSRYPDHMKRHDFFKSAMPEGYVQERTISFKDDGKYKTRAVVKFEGDTLVNRIELKGTDFKEDGNILGHKLEYNFNSHNVYITADKQKNGIKANFTVRHNVEDGSVQLADHYQQNTPIGDGPVLLPDNHYLSTQTVLSKDPNEKGSSGSEIQALEEKNAQLKQEIAALEEKNQALKYGGSGGSRDHMVLHEYVNAAGITGSPGGSPGGSGSPGGSPGGSKIAALKAENAALEAKIAALKAEIAALEAGY*
	ngGFP: black; amino acid point of insertion: red; P7: green; linker: gray; N8:yellow
nLuc_5gs:P7:5gs:cLuc:AU1:gs6:SbMVs:gs6:N8 (A logic function)	MGSGEDAKNIKKGPAPFYPLEDGTAGEQLHKAMKRYALVPGTIAFTDAHIEVDITYAEYFEMSVRLAEAMKRYGLNTNHRIVVCSENSLQFFMPVLGALFIGVAVAPANDIYNERELLNSMGISQPTVVFVSKKGLQKILNVQKKLPIIQKIIIMDSKTDYQGFQSMYTFVTSHLPPGFNEYDFVPESFDRDKTIALIMNSSGSTGLPKGVALPHRTACVRFSHARDPIFGNQIIPDTAILSVVPFHHGFGMFTTLGYLICGFRVVLMYRFEEELFLRSLQDYKIQSALLVPTLFSFFAKSTLIDKYDLSNLHEIASGGAPLSKEVGEAVAKRFHLPGIRQGYGLTETTSAILITPEGDDKPGAVGKVVPFFEAKVVDLDTGKTLGVNQRGELCVRGPMIMSGYVNNPEATNALIDKDGWLHSGDIAYWDEDEHFFIVDRLKSLIKYKGYQVAPAELESILLQHPNIFDAGVAGLPDDDAGELPAAVVVLEHGKGSSGSEIQALEEKNAQLKQEIAALEEKNQALKYGGSGGSTMTEKEIVDYVASQVTTAKKLRGGVVFVDEVPKGLTGKLDARKIREILIKAKKGGKIAVNSGSGYPYDVPDYAGGSGGGESVSLQSGGSGGSKIAALKAENAALEAKIAALKAEIAALEAGY*
	nLuc: black; linker: gray; P7: green cLuc:purple; HA tag magenta; SBMVs:dark red; N8 yellow
N8:gs6:PPvs:gs6:nLuc:5gs:P7:5gs:cLuc (B logic function)	MKIAALKAENAALEAKIAALKAEIAALEAGYGGSGGSNVVVHQAGSGGGSGSGEDAKNIKKGPAPFYPLEDGTAGEQLHKAMKRYALVPGTIAFTDAHIEVDITYAEYFEMSVRLAEAMKRYGLNTNHRIVVCSENSLQFFMPVLGALFIGVAVAPANDIYNERELLNSMGISQPTVVFVSKKGLQKILNVQKKLPIIQKIIIMDSKTDYQGFQSMYTFVTSHLPPGFNEYDFVPESFDRDKTIALIMNSSGSTGLPKGVALPHRTACVRFSHARDPIFGNQIIPDTAILSVVPFHHGFGMFTTLGYLICGFRVVLMYRFEEELFLRSLQDYKIQSALLVPTLFSFFAKSTLIDKYDLSNLHEIASGGAPLSKEVGEAVAKRFHLPGIRQGYGLTETTSAILITPEGDDKPGAVGKVVPFFEAKVVDLDTGKTLGVNQRGELCVRGPMIMSGYVNNPEATNALIDKDGWLHSGDIAYWDEDEHFFIVDRLKSLIKYKGYQVAPAELESILLQHPNIFDAGVAGLPDDDAGELPAAVVVLEHGKGSSGSEIQALEEKNAQLKQEIAALEEKNQALKYGGSGGSTMTEKEIVDYVASQVTTAKKLRGGVVFVDEVPKGLTGKLDARKIREILIKAKKGGKIAVNSGSGYPYDVPDYA*
	N8 yellow; linker: gray; PPVs: blue; nLuc: black; P7: green; cLuc:purple; HA tag magenta;
nLuc:5gs:P7:5gs:cLuc:AU1:gs20:N8:SbMVs:P7A (NOT B logic function)	MGSGEDAKNIKKGPAPFYPLEDGTAGEQLHKAMKRYALVPGTIAFTDAHIEVDITYAEYFEMSVRLAEAMKRYGLNTNHRIVVCSENSLQFFMPVLGALFIGVAVAPANDIYNERELLNSMGISQPTVVFVSKKGLQKILNVQKKLPIIQKIIIMDSKTDYQGFQSMYTFVTSHLPPGFNEYDFVPESFDRDKTIALIMNSSGSTGLPKGVALPHRTACVRFSHARDPIFGNQIIPDTAILSVVPFHHGFGMFTTLGYLICGFRVVLMYRFEEELFLRSLQDYKIQSALLVPTLFSFFAKSTLIDKYDLSNLHEIASGGAPLSKEVGEAVAKRFHLPGIRQGYGLTETTSAILITPEGDDKPGAVGKVVPFFEAKVVDLDTGKTLGVNQRGELCVRGPMIMSGYVNNPEATNALIDKDGWLHSGDIAYWDEDEHFFIVDRLKSLIKYKGYQVAPAELESILLQHPNIFDAGVAGLPDDDAGELPAAVVVLEHGKGSSGSEIQALEEKNAQLKQEIAALEEKNQALKYGGSGGSTMTEKEIVDYVASQVTTAKKLRGGVVFVDEVPKGLTGKLDARKIREILIKAKKGGKIAVNSGSGYPYDVPDYAGSPGGSPGGSGSPGGSPGGSKIAALKAENAALEAKIAALKAEIAALEAGYGGSGGGESVSLQSGGSGGSGEIAALEAKNAALKAEIAALEAKNAALKAGC*
	nLuc: black; linker: gray; P7: green cLuc:purple; HA tag magenta; N8 yellow; SBMVs:dark red; P7A: cyan
N8:gs6:PPVs:gs6:nLuc:5gs:P7:5gs:cLuc:AU1:gs6:SbMVs:gs6:N8	MKIAALKAENAALEAKIAALKAEIAALEAGYGGSGGSNVVVHQAGSGGGSGSGEDAKNIKKGPAPFYPLEDGTAGEQLHKAMKRYALVPGTIAFTDAHIEVDITYAEYFEMSVRLAEAMKRYGLNTNHRIVVCSENSLQFFMPVLGALFIGVAVAPANDIYNERELLNSMGISQPTVVFVSKKGLQKILNVQKKLPIIQKIIIMDSKTDYQGFQSMYTFVTSHLPPGFNEYDFVPESFDRDKTIALIMNSSGSTGLPKGVALPHRTACVRFSHARDPIFGNQIIPDTAILSVVPFHHGFGMFTTLGYLICGFRVVLMYRFEEELFLRSLQDYKIQSALLVPTLFSFFAKSTLIDKYDLSNLHEIASGGAPLSKEVGEAVAKRFHLPGIRQGYGLTETTSAILITPEGDDKPGAVGKVVPFFEAKVVDLDTGKTLGVNQRGELCVRGPMIMSGYVNNPEATNALIDKDGWLHSGDIAYWDEDEHFFIVDRLKSLIKYKGYQVAPAELESILLQHPNIFDAGVAGLPD

(AND logic function)	DDAGELPAAVVVLEHGKGSSGSEIQALEEKNAQLKQEIAALEEKNQALKYGGSGGSTMTEKEIVDYVASQVTTAKKLRGGVVFVDEVPKGLTGKLDARKIREILIKAKKGGKIAVNSGSGYPYDVPDYAGGSGGGESVSLQSGGSGGSKIAALKAENAALEAKIAALKAEIAALEAGY*
	N8 yellow; linker: gray; PPVs: blue; nLuc: black; P7: green; cLuc:purple; HA tag magenta; SBMVs:dark
nLuc:5gs:P7:5gs:cLuc:AU1:gs:PPVs:gs:SbMVs:gs:N8 (OR logic function)	MGSGEDAKNIKKGPAPFYPLEDGTAGEQLHKAMKRYALVPGTIAFTDAHIEVDITYAEYFEMSVRLAEAMKRYGLNTNHRIVVCSENSLQFFMPVLGALFIGVAVAPANDIYNERELLNSMGISQPTVVFVSKKGLQKILNVQKKLPIIQKIIIMDSKTDYQGFQSMYTFVTSHLPPGFNEYDFVPESFDRDKTIALIMNSSGSTGLPKGVALPHRTACVRFSHARDPIFGNQIIPDTAILSVVPFHHGFGMFTTLGYLICGFRVVLMYRFEEELFLRSLQDYKIQSALLVPTLFSFFAKSTLIDKYDLSNLHEIASGGAPLSKEVGEAVAKRFHLPGIRQGYGLTETTSAILITPEGDDKPGAVGKVVPFFEAKVVDLDTGKTLGVNQRGELCVRGPMIMSGYVNNPEATNALIDKDGWLHSGDIAYWDEDEHFFIVDRLKSLIKYKGYQVAPAELESILLQHPNIFDAGVAGLPDDDAGELPAAVVVLEHGKGSSGSEIQALEEKNAQLKQEIAALEEKNQALKYGGSGGSTMTEKEIVDYVASQVTTAKKLRGGVVFVDEVPKGLTGKLDARKIREILIKAKKGGKIAVNSGSGYPYDVPDYAGSNVVVHQAGESVSLQSGSKIAALKAENAALEAKIAALKAEIAALEAGY*
	nLuc: black; linker: gray; P7: green; cLuc:purple; HA tag magenta; PPVs: blue; SBMVs:dark; N8 yellow;
N8:gs6:PPVs:gs6:nLuc:5gs:P7:5gs:cLucAU1:20gs:N8:gs6:SbMVs:gs6:P7A (A nimply B logic function)	MYGKIAALKAENAALEAKIAALKAEIAALEAGYGGSGGSNVVVHQAGSGGGGSGSGEDAKNIKKGPAPFYPLEDGTAGEQLHKAMKRYALVPGTIAFTDAHIEVDITYAEYFEMSVRLAEAMKRYGLNTNHRIVVCSENSLQFFMPVLGALFIGVAVAPANDIYNERELLNSMGISQPTVVFVSKKGLQKILNVQKKLPIIQKIIIMDSKTDYQGFQSMYTFVTSHLPPGFNEYDFVPESFDRDKTIALIMNSSGSTGLPKGVALPHRTACVRFSHARDPIFGNQIIPDTAILSVVPFHHGFGMFTTLGYLICGFRVVLMYRFEEELFLRSLQDYKIQSALLVPTLFSFFAKSTLIDKYDLSNLHEIASGGAPLSKEVGEAVAKRFHLPGIRQGYGLTETTSAILITPEGDDKPGAVGKVVPFFEAKVVDLDTGKTLGVNQRGELCVRGPMIMSGYVNNPEATNALIDKDGWLHSGDIAYWDEDEHFFIVDRLKSLIKYKGYQVAPAELESILLQHPNIFDAGVAGLPDDDAGELPAAVVVLEHGKGSSGSEIQALEEKNAQLKQEIAALEEKNQALKYGGSGGSTMTEKEIVDYVASQVTTAKKLRGGVVFVDEVPKGLTGKLDARKIREILIKAKKGGKIAVNSGSGYPYDVPDYAGSPGGSPGGSGSPGGSPGGSKIAALKAENAALEAKIAALKAEIAALEAGYGGSGGGESVSLQSGGSGGSGEIAALEAKNAALKAEIAALEAKNAALKAGC*
	N8 yellow; linker: gray; PPVs: blue; nLuc: black; P7: green; cLuc:purple; HA tag magenta; SBMVs:dark; P7A: cyan
nLuc:5gs:P7:5gs:cLuc:AU1:gs6:SbMVs:gs6:N8:6gs:PPVs:6gs:P7A (A imply B logic function)	MGSGEDAKNIKKGPAPFYPLEDGTAGEQLHKAMKRYALVPGTIAFTDAHIEVDITYAEYFEMSVRLAEAMKRYGLNTNHRIVVCSENSLQFFMPVLGALFIGVAVAPANDIYNERELLNSMGISQPTVVFVSKKGLQKILNVQKKLPIIQKIIIMDSKTDYQGFQSMYTFVTSHLPPGFNEYDFVPESFDRDKTIALIMNSSGSTGLPKGVALPHRTACVRFSHARDPIFGNQIIPDTAILSVVPFHHGFGMFTTLGYLICGFRVVLMYRFEEELFLRSLQDYKIQSALLVPTLFSFFAKSTLIDKYDLSNLHEIASGGAPLSKEVGEAVAKRFHLPGIRQGYGLTETTSAILITPEGDDKPGAVGKVVPFFEAKVVDLDTGKTLGVNQRGELCVRGPMIMSGYVNNPEATNALIDKDGWLHSGDIAYWDEDEHFFIVDRLKSLIKYKGYQVAPAELESILLQHPNIFDAGVAGLPDDDAGELPAAVVVLEHGKGSSGSEIQALEEKNAQLKQEIAALEEKNQALKYGGSGGSTMTEKEIVDYVASQVTTAKKLRGGVVFVDEVPKGLTGKLDARKIREILIKAKKGGKIAVNSGSGYPYDVPDYAGGSGGGESVSLQSGGSGGSKIAALKAENAALEAKIAALKAEIAALEAGYGGSGGGNVVVHQAGGSGGSGEIAALEAKNAALKAEIAALEAKNAALKAGC*
	nLuc: black; linker: gray; P7: green; cLuc:purple; HA tag magenta; SBMVs:dark; N8 yellow;; PPVs: blue; P7A: cyan
nLuc:5gs:P7:5gs:cLucAU1:gs20:N8:PPVs:P7A (NOT A logic function)	MGSGEDAKNIKKGPAPFYPLEDGTAGEQLHKAMKRYALVPGTIAFTDAHIEVDITYAEYFEMSVRLAEAMKRYGLNTNHRIVVCSENSLQFFMPVLGALFIGVAVAPANDIYNERELLNSMGISQPTVVFVSKKGLQKILNVQKKLPIIQKIIIMDSKTDYQGFQSMYTFVTSHLPPGFNEYDFVPESFDRDKTIALIMNSSGSTGLPKGVALPHRTACVRFSHARDPIFGNQIIPDTAILSVVPFHHGFGMFTTLGYLICGFRVVLMYRFEEELFLRSLQDYKIQSALLVPTLFSFFAKSTLIDKYDLSNLHEIASGGAPLSKEVGEAVAKRFHLPGIRQGYGLTETTSAILITPEGDDKPGAVGKVVPFFEAKVVDLDTGKTLGVNQRGELCVRGPMIMSGYVNNPEATNALIDKDGWLHSGDIAYWDEDEHFFIVDRLKSLIKYKGYQVAPAELESILLQHPNIFDAGVAGLPDDDAGELPAAVVVLEHGKGSSGSEIQALEEKNAQLKQEIAALEEKNQALKYGGSGGSTMTEKEIVDYVASQVTTAKKLRGGVVFVDEVPKGLTGKLDARKIREILIKAKKGGKIAVNSGSGYPYDVPDYAGSPGGSPGGSGSPGGSPGGSKIAALKAENAALEAKIAALKAEIAALEAGYGGSGGGNVVVHQAGGSGGSGEIAALEAKNAALKAEIAALEAKNAALKAGC*
	nLuc: black; linker: gray; P7: green cLuc:purple; HA tag magenta; N8 yellow; PPVs: blue; P7A: cyan
nLuc:5gs:P7:5gs:cLucAU1:gs6:PPVs:gs6:N8:6gs:SbMVs:6gs:P7A (B imply A logic function)	MGSGEDAKNIKKGPAPFYPLEDGTAGEQLHKAMKRYALVPGTIAFTDAHIEVDITYAEYFEMSVRLAEAMKRYGLNTNHRIVVCSENSLQFFMPVLGALFIGVAVAPANDIYNERELLNSMGISQPTVVFVSKKGLQKILNVQKKLPIIQKIIIMDSKTDYQGFQSMYTFVTSHLPPGFNEYDFVPESFDRDKTIALIMNSSGSTGLPKGVALPHRTACVRFSHARDPIFGNQIIPDTAILSVVPFHHGFGMFTTLGYLICGFRVVLMYRFEEELFLRSLQDYKIQSALLVPTLFSFFAKSTLIDKYDLSNLHEIASGGAPLSKEVGEAVAKRFHLPGIRQGYGLTETTSAILITPEGDDKPGAVGKVVPFFEAKVVDLDTGKTLGVNQRGELCVRGPMIMSGYVNNPEATNALIDKDGWLHSGDIAYWDEDEHFFIVDRLKSLIKYKGYQVAPAELESILLQHPNIFDAGVAGLPDDDAGELPAAVVVLEHGKGSSGSEIQALEEKNAQLKQEIAALEEKNQALKYGGSGGSTMTEKEIVDYVASQVTTAKKLRGGVVFVDEVPKGLTGKLDARKIREILIKAKKGGKIAVNSGSGYPYDVPDYAGGSGGGNVVVHQAGGSGGSKIAALKAENAALEAKIAALKAEIAALEAGYGGSGGGESVSLQSGGSGGSGEIAALEAKNAALKAEIAALEAKNAALKAGC*
	nLuc: black; linker: gray; P7: green; cLuc:purple; HA tag magenta; PPVs: blue; N8 yellow;; SBMVs:dark; P7A: cyan
N8:gs6:SbMVs:gs6:nLuc:5gs:P7:5gs:cLucAU1:20gs:N8:gs6:PPVs:gs6:P7A (B nimply A logic function)	MYGKIAALKAENAALEAKIAALKAEIAALEAGYGGSGGSESVSLQSGSGGGGSGSGEDAKNIKKGPAPFYPLEDGTAGEQLHKAMKRYALVPGTIAFTDAHIEVDITYAEYFEMSVRLAEAMKRYGLNTNHRIVVCSENSLQFFMPVLGALFIGVAVAPANDIYNERELLNSMGISQPTVVFVSKKGLQKILNVQKKLPIIQKIIIMDSKTDYQGFQSMYTFVTSHLPPGFNEYDFVPESFDRDKTIALIMNSSGSTGLPKGVALPHRTACVRFSHARDPIFGNQIIPDTAILSVVPFHHGFGMFTTLGYLICGFRVVLMYRFEEELFLRSLQDYKIQSALLVPTLFSFFAKSTLIDKYDLSNLHEIASGGAPLSKEVGEAVAKRFHLPGIRQGYGLTETTSAILITPEGDDKPGAVGKVVPFFEAKVVDLDTGKTLGVNQRGELCVRGPMIMSGYVNNPEATNALIDKDGWLHSGDIAYWDEDEHFFIVDRLKSLIKYKGYQVAPAELESILLQHPNIFDAGVAGLPDDDAGELPAAVVVLEHGKGSSGSEIQALEEKNAQLKQEIAALEEKNQALKYGGSGGSTMTEKEIVDYVASQVTTAKKLRGGVVFVDEVPKGLTGKLDARKIREILIKAKKGGKIAVNSGSGYPYDVPDYAGSPGGSPGGSGSPGGSPGGSKIAALKAENAALEAKIAALKAEIAALEAGYGGSGGGNVVVHQAGGSGGSGEIAALEAKNAALKAEIAALEAKNAALKAGC*
	N8 yellow; linker: gray; PPVs: blue; nLuc: black; P7: green; cLuc:purple; HA tag magenta; SBMVs:dark; P7A: cyan
Lck N266_P7	MGCGCSSHPEDDWMENIDVCENCHYPIVPLDGKGTLLIRNGSEVRDPLVTYEGSNPPASPLQDNLVIALHSYEPSHDGDLGFEKGEQLRILEQSGEWWKAQSLTTGQEGFIPFNFVAKANSLEPEPWFFKNLSRKDAERQLLAPGNTHGSFLIRESESTAGSFSLSVRDFDQNQGEVVKHYKIRNLDNGGFYISPRITFPGLHELVRHYTNASDGLCTRLSRPCQTQKPQKPWWEDEWEVPRETLKLVERLGAGQFGEVWMGYYNGSSGSEIQALEEKNAQLKQEIAALEEKNQALKYGGSGGSHTKVAVKSLKQGSMSPDAFLAEANLMKQLQHQRLVRLYAVVTQEPIYIITEYMENGSLVDFLKTPSGIKLTINKLLDMAAQIAEGMAFIEERNYIHRDLRAANILVSDTLSCKIADFGLARLIEDNEYTAREGAKFPIKWTAPEAINYGTFTIKSDVWSFGILLTEIVTHGRIPYPGMTNPEVIQNLERGYRMVRPDNCPEELYQLMRLCWKERPEDRPTFDYLRSVLEDFFTATEGQFQPQP*
	Lck: black; linker: gray P7: green; amino acid point of insertion: red
Lck G278 P7	MGCGCSSHPEDDWMENIDVCENCHYPIVPLDGKGTLLIRNGSEVRDPLVTYEGSNPPASPLQDNLVIALHSYEPSHDGDLGFEKGEQLRILEQSGEWWKAQSLTTGQEGFIPFNFVAKANSLEPEPWFFKNLSRKDAERQLLAPGNTHGSFLIRESESTAGSFSLSVRDFDQNQGEVVKHYKIRNLDNGGFYISPRITFPGLHELVRHYTNASDGLCTRLSRPCQTQKPQKPWWEDEWEVPRETLKLVERLGAGQFGEVWMGYYNGHTKVAVKSLKQGGSSGSEIQALEEKNAQLKQEIAALEEKNQALKYGGSGGSSMSPDAFLAEANLMKQLQHQRLVRLYAVVTQEPIYIITEYMENGSLVDFLKTPSGIKLTINKLLDMAAQIAEGMAFIEERNYIHRDLRAANILVSDTLSCKIADFGLARLIEDNEYTAREGAKFPIKWTAPEAINYGTFTIKSDVWSFGILLTEIVTHGRIPYPGMTNPEVIQNLERGYRMVRPDNCPEELYQLMRLCWKERPEDRPTFDYLRSVLEDFFTATEGQYQPQP*
	Lck: black; linker: gray P7: green; amino acid point of insertion: red
Lck T375 P7	MGCGCSSHPEDDWMENIDVCENCHYPIVPLDGKGTLLIRNGSEVRDPLVTYEGSNPPASPLQDNLVIALHSYEPSHDGDLGFEKGEQLRILEQSGEWWKAQSLTTGQEGFIPFNFVAKANSLEPEPWFFKNLSRKDAERQLLAPGNTHGSFLIRESESTAGSFSLSVRDFDQNQGEVVKHYKIRNLDNGGFYISPRITFPGLHELVRHYTNASDGLCTRLSRPCQTQKPQKPWWEDEWEVPRETLKLVERLGAGQFGEVWMGYYNGHTKVAVKSLKQGSMSPDAFLAEANLMKQLQHQRLVRLYAVVTQEPIYIITEYMENGSLVDFLKTPSGIKLTINKLLDMAAQIAEGMAFIEERNYIHRDLRAANILVSDTSPGGSEIQALEEKNAQLKQEIAALEEKNQALKYGGGSGGGLSCKIADFGLARLIEDNEYTAREGAKFPIKWTAPEAINYGTFTIKSDVWSFGILLTEIVTHGRIPYPGMTNPEVIQNLERGYRMVRPDNCPEELYQLMRLCWKERPEDRPTFDYLRSVLEDFFTATEGQYQPQP

	Lck: black; linker: gray P7: green; amino acid point of insertion: red
Lck Q256 P7	MGCGCSSHPEDDWMENIDVCENCHYPIVPLDGKGTLLIRNGSEVRDPLVTYEGSNPPASPLQDNLVIALHSYEPSHDGDLGFEKGEQLRILEQSGEWWKAQSLTTGQEGFIPFNFVAKANSLEPEPWFFKNLSRKDAERQLLAPGNTHGSFLIRESESTAGSFSLSVRDFDQNQGEVVKHYKIRNLDNGGFYISPRITFPGLHELVRHYTNASDGLCTRLSRPCQTQKPQKPWWEDEWEVPRETLKLVERLGAGQSPGGSEIQALEEKNAQLKQEIAALEEKNQALKYGGGSGGGFGEVWMGYYNGHTKVAVKSLKQGSMSPDAFLAEANLMKQLQHQRLVRLYAVVTQEPIYIITEYMENGSLVDFLKTPSGIKLTINKLLDMAAQIAEGMAFIEERNYIHRDLRAANILVSDTLSCKIADFGLARLIEDNEYTAREGAKFPIKWTAPEAINYGTFTIKSDVWSFGILLTEIVTHGRIPYPGMTNPEVIQNLERGYRMVRPDNCPEELYQLMRLCWKERPEDRPTFDYLRSVLEDFFTATEGQYQPQP
	Lck: black; linker: gray P7: green; amino acid point of insertion: red
CAR T N184 P7	MALPVTALLLPLALLLHAARPEQKLISEEDLDIQMTQTTSSLSASLGDRVTISCRASQDISKYLNWYQQKPDGTVKLLIYHTSRLHSGVPSRFSGSGSGTDYSLTISNLEQEDIATYFCQQGNTLPYTFGGGTKLEITGGGGSGGGGSGGGGSEVKLQESGPGLVAPSQSLSVTCTVSGVSLPDYGVSWIRQPPRKGLEWLGVIWGSETTYYNSGSSGSEIQALEEKNAQLKQEIAALEEKNQALKYGGSGGSALKSRLTIIKDNSKSQVFLKMNSLQTDDTAIYYCAKHYYYGGSYAMDYWGQGTSVTVSSTTTPAPRPPTPAPTIASQPLSLRPEACRPAAGGAVHTRGLDFACDIYIWAPLAGTCGVLLLSLVITLYCKRGRKKLLYIFKQPFMRPVQTTQEEDGCSCRFPEEEEGGCELRVKFSRSADAPAYQQGQNQLYNELNLGRREEYDVLDKRRGRDPEMGGKPRRKNPQEGLYNELQKDKMAEAYSEIGMKGERRRGKGHDGLYQGLSTATKDTYDALHMQALPPR*
	CD8 leader: blue; myc Tag:brown; linker: gray; scFV: black; P7: green; CD8 hinge: purple; cd8 TM: red; 41bb: magenta; cd3z:cyan; amino acid point of insertion: red
CAR T P7N 195	MALPVTALLLPLALLLHAARPEQKLISEEDLDIQMTQTTSSLSASLGDRVTISCRASQDISKYLNWYQQKPDGTVKLLIYHTSRLHSGVPSRFSGSGSGTDYSLTISNLEQEDIATYFCQQGNTLPYTFGGGTKLEITGGGGSGGGGSGGGGSEVKLQESGPGLVAPSQSLSVTCTVSGVSLPDYGVSWIRQPPRKGLEWLGVIWGSETTYYNSALKSRLTIIKDNGSSGSEIQALEEKNAQLKQEIAALEEKNQALKYGGSGGSSKSQVFLKMNSLQTDDTAIYYCAKHYYYGGSYAMDYWGQGTSVTVSSTTTPAPRPPTPAPTIASQPLSLRPEACRPAAGGAVHTRGLDFACDIYIWAPLAGTCGVLLLSLVITLYCKRGRKKLLYIFKQPFMRPVQTTQEEDGCSCRFPEEEEGGCELRVKFSRSADAPAYQQGQNQLYNELNLGRREEYDVLDKRRGRDPEMGGKPRRKNPQEGLYNELQKDKMAEAYSEIGMKGERRRGKGHDGLYQGLSTATKDTYDALHMQALPPR*
	CD8 leader: blue; myc Tag:brown; linker: gray; scFV: black; P7: green; CD8 hinge: purple; cd8 TM: red; 41bb: magenta; cd3z:cyan; amino acid point of insertion: red
CAR T T51 P7	MALPVTALLLPLALLLHAARPEQKLISEEDLDIQMTQTTSSLSASLGDRVTISCRASQDISKYLNWYQQKPDGTVKLLIYHTGSSGSEIQALEEKNAQLKQEIAALEEKNQALKYGGSGGSSRLHSGVPSRFSGSGSGTDYSLTISNLEQEDIATYFCQQGNTLPYTFGGGTKLEITGGGGSGGGGSGGGGSEVKLQESGPGLVAPSQSLSVTCTVSGVSLPDYGVSWIRQPPRKGLEWLGVIWGSETTYYNSALKSRLTIIKDNSKSQVFLKMNSLQTDDTAIYYCAKHYYYGGSYAMDYWGQGTSVTVSSTTTPAPRPPTPAPTIASQPLSLRPEACRPAAGGAVHTRGLDFACDIYIWAPLAGTCGVLLLSLVITLYCKRGRKKLLYIFKQPFMRPVQTTQEEDGCSCRFPEEEEGGCELRVKFSRSADAPAYQQGQNQLYNELNLGRREEYDVLDKRRGRDPEMGGKPRRKNPQEGLYNELQKDKMAEAYSEIGMKGERRRGKGHDGLYQGLSTATKDTYDALHMQALPPR*
	CD8 leader: blue; myc Tag:brown; linker: gray; scFV: black; P7: green; CD8 hinge: purple; cd8 TM: red; 41bb: magenta; cd3z:cyan; amino acid point of insertion: red
CAR T I193 P7	MALPVTALLLPLALLLHAARPEQKLISEEDLDIQMTQTTSSLSASLGDRVTISCRASQDISKYLNWYQQKPDGTVKLLIYHTSRLHSGVPSRFSGSGSGTDYSLTISNLEQEDIATYFCQQGNTLPYTFGGGTKLEITGGGGSGGGGSGGGGSEVKLQESGPGLVAPSQSLSVTCTVSGVSLPDYGVSWIRQPPRKGLEWLGVIWGSETTYYNSALKSRLTIIGSSGSEIQALEEKNAQLKQEIAALEEKNQALKYGGSGGSKDNSKSQVFLKMNSLQTDDTAIYYCAKHYYYGGSYAMDYWGQGTSVTVSSTTTPAPRPPTPAPTIASQPLSLRPEACRPAAGGAVHTRGLDFACDIYIWAPLAGTCGVLLLSLVITLYCKRGRKKLLYIFKQPFMRPVQTTQEEDGCSCRFPEEEEGGCELRVKFSRSADAPAYQQGQNQLYNELNLGRREEYDVLDKRRGRDPEMGGKPRRKNPQEGLYNELQKDKMAEAYSEIGMKGERRRGKGHDGLYQGLSTATKDTYDALHMQALPPR*
	CD8 leader: blue; myc Tag:brown; linker: gray; scFV: black; P7: green; CD8 hinge: purple; cd8 TM: red; 41bb: magenta; cd3z:cyan
CAR T S199 P7	MALPVTALLLPLALLLHAARPEQKLISEEDLDIQMTQTTSSLSASLGDRVTISCRASQDISKYLNWYQQKPDGTVKLLIYHTSRLHSGVPSRFSGSGSGTDYSLTISNLEQEDIATYFCQQGNTLPYTFGGGTKLEITGGGGSGGGGSGGGGSEVKLQESGPGLVAPSQSLSVTCTVSGVSLPDYGVSWIRQPPRKGLEWLGVIWGSETTYYNSALKSRLTIIKDNSKSGSSGSEIQALEEKNAQLKQEIAALEEKNQALKYGGSGGSQVFLKMNSLQTDDTAIYYCAKHYYYGGSYAMDYWGQGTSVTVSSTTTPAPRPPTPAPTIASQPLSLRPEACRPAAGGAVHTRGLDFACDIYIWAPLAGTCGVLLLSLVITLYCKRGRKKLLYIFKQPFMRPVQTTQEEDGCSCRFPEEEEGGCELRVKFSRSADAPAYQQGQNQLYNELNLGRREEYDVLDKRRGRDPEMGGKPRRKNPQEGLYNELQKDKMAEAYSEIGMKGERRRGKGHDGLYQGLSTATKDTYDALHMQALPPR*
	CD8 leader: blue; myc Tag:brown; linker: gray; scFV: black; P7: green; CD8 hinge: purple; cd8 TM: red; 41bb: magenta; cd3z:cyan
CAR T N5_P7N 195	MALPVTALLLPLALLLHAARPEQKLISEEDLKIAALKAEIAALEAENAALEAKIAALKAGGSSGSDIQMTQTTSSLSASLGDRVTISCRASQDISKYLNWYQQKPDGTVKLLIYHTSRLHSGVPSRFSGSGSGTDYSLTISNLEQEDIATYFCQQGNTLPYTFGGGTKLEITGGGGSGGGGSGGGGSEVKLQESGPGLVAPSQSLSVTCTVSGVSLPDYGVSWIRQPPRKGLEWLGVIWGSETTYYNSALKSRLTIIKDNGSSGSEIQALEEKNAQLKQEIAALEEKNQALKYGGSGGSSKSQVFLKMNSLQTDDTAIYYCAKHYYYGGSYAMDYWGQGTSVTVSSTTTPAPRPPTPAPTIASQPLSLRPEACRPAAGGAVHTRGLDFACDIYIWAPLAGTCGVLLLSLVITLYCKRGRKKLLYIFKQPFMRPVQTTQEEDGCSCRFPEEEEGGCELRVKFSRSADAPAYQQGQNQLYNELNLGRREEYDVLDKRRGRDPEMGGKPRRKNPQEGLYNELQKDKMAEAYSEIGMKGERRRGKGHDGLYQGLSTATKDTYDALHMQALPPR*
	CD8 leader: blue; myc Tag:brown; N5: orange linker: gray; scFV: black; P7: green; CD8 hinge: purple; cd8 TM: red; 41bb: magenta; cd3z:cyan; amino acid point of insertion: red
CAR T (Her2) N5_P7_199	MALPVTALLLPLALLLHAARPEQKLISEEDLKIAALKAEIAALEAENAALEAKIAALKAGGSSGSDIQMTQSPSSLSASVGDRVTITCRASQDVNTAVAWYQQKPGKAPKLLIYSASFLYSGVPSRFSGSRSGTDFTLTISSLQPEDFATYYCQQHYTTPPTFGQGTKVEIKRTGSTSGSGKPGSGEGSEVQLVESGGGLVQPGGSLRLSCAASGFNIKDTYIHWVRQAPGKGLEWVARIYPTNGYTRYADSVKGRFTISADTSGSSGSEIQALEEKNAQLKQEIAALEEKNQALKYGGSGGSKNTAYLQMNSLRAEDTAVYYCSRWGGDGFYAMDVWGQGTLVTVSSTTTPAPRPPTPAPTIASQPLSLRPEACRPAAGGAVHTRGLDFACDIYIWAPLAGTCGVLLLSLVITLYCKRGRKKLLYIFKQPFMRPVQTTQEEDGCSCRFPEEEEGGCELRVKFSRSADAPAYQQGQNQLYNELNLGRREEYDVLDKRRGRDPEMGGKPRRKNPQEGLYNELQKDKMAEAYSEIGMKGERRRGKGHDGLYQGLSTATKDTYDALHMQALPPR*
	CD8 leader: blue; myc Tag:brown; N5: orange linker: gray; scFV: black; P7: green; CD8 hinge: purple; cd8 TM: red; 41bb: magenta; cd3z:cyan
ngGFP:P8A	MGHHHHHHHHMVSKGEEDNMASLPATHELHIFGSINGVDFDMVGQGTGNPNDGYEELNLKSTKGDLQFSPWILVPHIGYGFHQYLPYPDGMSPFQAAMVDGSGYQVHRTMQFEDGASLTVNYRYTYEGSHIKGEAQVKGTGFPADGPVMTNSLTAADWCRSKKTYPNDKTIISTFKWSYTTGNGKRYRSTARTTYTFAKPMAANYLKNQPMYVFRKTELKHSKTELNFKEWQKAFTDVMGMDELYKGGSGGGGSGGGSGGSKIAALKAENAALEAKIAALKAENAALEAGGC*
	ngGFP:black; his tag: dark blue; linker: gray; P8A: dak yellow
ngGFP:N5:gs:N 8	MHHHHHHHHHMVSKGEEDNMASLPATHELHIFGSINGVDFDMVGQGTGNPNDGYEELNLKSTKGDLQFSPWILVPHIGYGFHQYLPYPDGMSPFQAAMVDGSGYQVHRTMQFEDGASLTVNYRYTYEGSHIKGEAQVKGTGFPADGPVMTNSLTAADWSRSKKTYPNDKTIISTFKWSYTTGNGKRYRSTARTTYTFAKPMAANYLKNQPMYVFRKTELKHSKTELNFKEWQKAFTDVMGMDELYKGGSGGGSGGSGEIAALEAKIAALKAKNAALKAEIAALEAGSPGGSPGGSGSPGGSPGGSGSPGGSPGGSGSPGGSPGGSYGKIAALKAENAALEAKIAALKAEIAALEAGY*
	ngGFP: black; his tag: dark blue; N5: purple, linker: gray; N8: yellow

Supplementary table S2

ANOVA summary	confidence interval	F	P value	P value summary	Significant diff. among means (P < 0.05)?	R squared	Dunnett's multiple comparisons test	Mean Diff,	95,00% CI of diff,	Significant?	Summary	Adjusted P Value
**Fig 1C (Luc_N8)**												
Ordinary one-way ANOVA	95	173	2,354E-12	****	Yes	0,9788	0 ng N7 vs. 1 ng N7	1,142	0,9658 to 1,318	Yes	****	<0,0001
							0 ng N7 vs. 2,5 ng N7	1,354	1,178 to 1,530	Yes	****	<0,0001
							0 ng N7 vs. 5 ng N7	1,391	1,214 to 1,567	Yes	****	<0,0001
							0 ng N7 vs. 10 ng N7	1,404	1,228 to 1,580	Yes	****	<0,0001
**Fig 1C (Luc_5gs:N8)**												
Ordinary one-way ANOVA	95	154,1	5,5E-12	****	Yes	0,9762	0 ng N7 vs. 1 ng N7	2,065	1,765 to 2,364	Yes	****	<0,0001
							0 ng N7 vs. 2,5 ng N7	2,159	1,859 to 2,459	Yes	****	<0,0001
							0 ng N7 vs. 5 ng N7	2,179	1,879 to 2,478	Yes	****	<0,0001
							0 ng N7 vs. 10 ng N7	2,209	1,909 to 2,508	Yes	****	<0,0001
**Fig 1C (Luc_10gs:N8)**												
Ordinary one-way ANOVA	95	844	< 1,0E-15	****	Yes	0,9956	0 ng N7 vs. 1 ng N7	2,826	2,669 to 2,983	Yes	****	<0,0001
							0 ng N7 vs. 2,5 ng N7	2,605	2,447 to 2,762	Yes	****	<0,0001
							0 ng N7 vs. 5 ng N7	2,559	2,401 to 2,716	Yes	****	<0,0001
							0 ng N7 vs. 10 ng N7	2,569	2,412 to 2,727	Yes	****	<0,0001
**Fig 1D**												
Ordinary one-way ANOVA	95	131,9	< 1,0E-15	****	Yes	0,9764	Luc_5gs:N8 vs. 1 ng P7SN	-0,1853	-0,5386 to 0,1681	**No**	**ns**	0,6845
							Luc_5gs:N8 vs. 2,5 ng P7SN	-0,1808	-0,5341 to 0,1726	**No**	**ns**	0,7124
							Luc_5gs:N8 vs. 5 ng P7SN	-0,0985	-0,4519 to 0,2549	**No**	**ns**	0,991
							Luc_5gs:N8 vs. 10 ng P7SN	0,0075	-0,3459 to 0,3609	**No**	**ns**	>0,9999
							Luc_5gs:N8 vs. 1 ng P7	0,1675	-0,1859 to 0,5209	**No**	**ns**	0,7913
							Luc_5gs:N8 vs. 2,5 ng P7	-0,2938	-0,6471 to 0,05963	**No**	**ns**	0,1558
							Luc_5gs:N8 vs. 5 ng P7	-0,05625	-0,4096 to 0,2971	**No**	**ns**	0,9993
							Luc_5gs:N8 vs. 10 ng P7	0,3735	0,02012 to 0,7269	Yes	*	0,0327
							Luc_5gs:N8 vs. 1 ng P7A	0,3525	-0,0008813 to 0,7059	**No**	**ns**	0,0509
							Luc_5gs:N8 vs. 2,5 ng P7A	1,204	0,8504 to 1,557	Yes	****	<0,0001
							Luc_5gs:N8 vs. 5 ng P7A	1,308	0,9544 to 1,661	Yes	****	<0,0001
							Luc_5gs:N8 vs. 10 ng P7A	1,595	1,241 to 1,948	Yes	****	<0,0001
							Luc_5gs:N8 vs. 1 ng N7	2,065	1,711 to 2,418	Yes	****	<0,0001
							Luc_5gs:N8 vs. 2,5 ng N7	2,159	1,806 to 2,512	Yes	****	<0,0001
							Luc_5gs:N8 vs. 5 ng N7	2,179	1,825 to 2,532	Yes	****	<0,0001
							Luc_5gs:N8 vs. 10 ng N7	2,209	1,855 to 2,562	Yes	****	<0,0001

ANOVA summary	confidence interval	F	P value	P value summary	Significant diff. among means (P < 0.05)?	R squared	Dunnett's multiple comparisons test	Mean Diff,	95,00% CI of diff,	Significant?	Summary	Adjusted P Value
**Fig 2B**												
Ordinary one-way ANOVA	95	55,15	2,96E-13	****	Yes	0,9415	nLuc:5gs:P7:5gs:cLuc:20gs:N8 vs. 1 ng N7	-0,6155	-1,267 to 0,03580	**No**	**ns**	0,0694
							nLuc:5gs:P7:5gs:cLuc:20gs:N8 vs. 3 ng N7	-1,406	-2,058 to -0,7550	Yes	****	<0,0001
							nLuc:5gs:P7:5gs:cLuc:20gs:N8 vs. 5 ng N7	-1,972	-2,623 to -1,320	Yes	****	<0,0001
							nLuc:5gs:P7:5gs:cLuc:20gs:N8 vs. 10 ng N7	-2,629	-3,280 to -1,977	Yes	****	<0,0001
							nLuc:5gs:P7:5gs:cLuc:20gs:N8 vs. 20 ng N7	-2,97	-3,621 to -2,318	Yes	****	<0,0001
							nLuc:5gs:P7:5gs:cLuc:20gs:N8 vs. 50 ng N7	-2,647	-3,299 to -1,996	Yes	****	<0,0001
							nLuc:5gs:P7:5gs:cLuc:20gs:N8 vs. 50 ng P4	0,02625	-0,6250 to 0,6775	**No**	**ns**	0,9999
**Fig 2C**												
Ordinary one-way ANOVA	95	96,06	3,6E-14	****	Yes	0,9648	nLuc:5gs:P7:5gs:cLuc:20gs:N8 vs. 1 µM N7	-1,314	-2,199 to -0,4279	Yes	**	0,0024
							nLuc:5gs:P7:5gs:cLuc:20gs:N8 vs. 3 µM N7	-1,928	-2,814 to -1,043	Yes	****	<0,0001
							nLuc:5gs:P7:5gs:cLuc:20gs:N8 vs. 5 µM N7	-2,583	-3,468 to -1,697	Yes	****	<0,0001
							nLuc:5gs:P7:5gs:cLuc:20gs:N8 vs. 10 µM N7	-4,409	-5,295 to -3,523	Yes	****	<0,0001
							nLuc:5gs:P7:5gs:cLuc:20gs:N8 vs. 20 µM N7	-5,892	-6,777 to -5,006	Yes	****	<0,0001
							nLuc:5gs:P7:5gs:cLuc:20gs:N8 vs. 20 µM P4	-0,017	-0,9026 to 0,8686	**No**	**ns**	>0,9999

Statistical analysis for logic functions using One-Way ANOVA with Tuckey's comparison

A function

Number of families	1
Number of comparisons per family	6
Alpha	0,05

Tukey's multiple comparisons test	Mean Diff,	95,00% CI of diff,	Significant?	Summary	Adjusted P Value	
Column A vs. Column B	-0,9358	-1,027 to -0,8449	Yes	****	<0,000000001	A-B
Column A vs. Column C	-0,001387	-0,09226 to 0,08949	No	ns	0,999964541	A-C
Column A vs. Column D	-0,9513	-1,042 to -0,8604	Yes	****	<0,000000001	A-D
Column B vs. Column C	0,9344	0,8435 to 1,025	Yes	****	<0,000000001	B-C
Column B vs. Column D	-0,01552	-0,1064 to 0,07535	No	ns	0,955828432	B-D
Column C vs. Column D	-0,9499	-1,041 to -0,8591	Yes	****	<0,000000001	C-D

Test details	Mean 1	Mean 2	Mean Diff,	SE of diff,	n1	n2	q	DF
Column A vs. Column B	0,04868	0,9845	-0,9358	0,03061	4	4	43,24	12
Column A vs. Column C	0,04868	0,05006	-0,001387	0,03061	4	4	0,0641	12
Column A vs. Column D	0,04868	1	-0,9513	0,03061	4	4	43,95	12
Column B vs. Column C	0,9845	0,05006	0,9344	0,03061	4	4	43,17	12
Column B vs. Column D	0,9845	1	-0,01552	0,03061	4	4	0,7172	12
Column C vs. Column D	0,05006	1	-0,9499	0,03061	4	4	43,89	12

B function

Number of families	1
Number of comparisons per family	6
Alpha	0,05

Tukey's multiple comparisons test	Mean Diff,	95,00% CI of diff,	Significant?	Summary	Adjusted P Value	
Column A vs. Column B	-0,013	-0,1394 to 0,1134	No	ns	0,98963051	A-B
Column A vs. Column C	-0,9831	-1,110 to -0,8567	Yes	****	<0,00000001	A-C
Column A vs. Column D	-0,9814	-1,108 to -0,8550	Yes	****	<0,00000001	A-D
Column B vs. Column C	-0,9701	-1,097 to -0,8437	Yes	****	<0,00000001	B-C
Column B vs. Column D	-0,9684	-1,095 to -0,8420	Yes	****	<0,00000001	B-D
Column C vs. Column D	0,001715	-0,1247 to 0,1282	No	ns	0,99997512	C-D

Test details	Mean 1	Mean 2	Mean Diff,	SE of diff,	n1	n2	q	DF
Column A vs. Column B	0,01688	0,02987	-0,013	0,04259	4	4	0,4315	12
Column A vs. Column C	0,01688	1	-0,9831	0,04259	4	4	32,65	12
Column A vs. Column D	0,01688	0,9983	-0,9814	0,04259	4	4	32,59	12
Column B vs. Column C	0,02987	1	-0,9701	0,04259	4	4	32,21	12
Column B vs. Column D	0,02987	0,9983	-0,9684	0,04259	4	4	32,16	12
Column C vs. Column D	1	0,9983	0,001715	0,04259	4	4	0,05696	12

AND function

Number of families	1
Number of comparisons per family	6
Alpha	0,05

Tukey's multiple comparisons test	Mean Diff,	95,00% CI of diff,	Significant?	Summary	Adjusted P Value	
Column A vs. Column B	0,0215	-0,09987 to 0,1429	No	ns	0,95115415	A-B
Column A vs. Column C	-0,01615	-0,1375 to 0,1052	No	ns	0,97814472	A-C
Column A vs. Column D	-0,9549	-1,076 to -0,8336	Yes	****	<0,00000001	A-D
Column B vs. Column C	-0,03765	-0,1590 to 0,08372	No	ns	0,79438664	B-C
Column B vs. Column D	-0,9764	-1,098 to -0,8551	Yes	****	<0,00000001	B-D
Column C vs. Column D	-0,9388	-1,060 to -0,8174	Yes	****	<0,00000001	C-D

Test details	Mean 1	Mean 2	Mean Diff,	SE of diff,	n1	n2	q	DF
Column A vs. Column B	0,04506	0,02356	0,0215	0,04088	4	4	0,7438	12
Column A vs. Column C	0,04506	0,06121	-0,01615	0,04088	4	4	0,5586	12
Column A vs. Column D	0,04506	1	-0,9549	0,04088	4	4	33,04	12
Column B vs. Column C	0,02356	0,06121	-0,03765	0,04088	4	4	1,302	12
Column B vs. Column D	0,02356	1	-0,9764	0,04088	4	4	33,78	12
Column C vs. Column D	0,06121	1	-0,9388	0,04088	4	4	32,48	12

A nimply B

Number of families	1
Number of comparisons per family	6
Alpha	0,05

Tukey's multiple comparisons test	Mean Diff,	95,00% CI of diff,	Significant?	Summary	Adjusted P Value	
Column A vs. Column B	-0,02691	-0,2258 to 0,1720	No	ns	0,97708579	A-B
Column A vs. Column C	-0,9793	-1,178 to -0,7804	Yes	****	0,00000003	A-C
Column A vs. Column D	-0,1432	-0,3422 to 0,05569	No	ns	0,19631112	A-D
Column B vs. Column C	-0,9524	-1,151 to -0,7535	Yes	****	0,00000004	B-C
Column B vs. Column D	-0,1163	-0,3153 to 0,08260	No	ns	0,34840856	B-D
Column C vs. Column D	0,8361	0,6371 to 1,035	Yes	****	0,00000016	C-D

Test details	Mean 1	Mean 2	Mean Diff,	SE of diff,	n1	n2	q	DF
Column A vs. Column B	0,02071	0,04762	-0,02691	0,067	4	4	0,568	12
Column A vs. Column C	0,02071	1	-0,9793	0,067	4	4	20,67	12
Column A vs. Column D	0,02071	0,1639	-0,1432	0,067	4	4	3,023	12
Column B vs. Column C	0,04762	1	-0,9524	0,067	4	4	20,1	12
Column B vs. Column D	0,04762	0,1639	-0,1163	0,067	4	4	2,455	12
Column C vs. Column D	1	0,1639	0,8361	0,067	4	4	17,65	12

B imply A

Number of families	1
Number of comparisons per family	6
Alpha	0,05

Tukey's multiple comparisons test	Mean Diff,	95,00% CI of diff,	Significant?	Summary	Adjusted P Value	
Column A vs. Column B	0,7013	0,5256 to 0,8770	Yes	****	0,00000029	A-B
Column A vs. Column C	-0,1965	-0,3722 to -0,02089	Yes	*	0,02704135	A-C
Column A vs. Column D	-0,1598	-0,3355 to 0,01585	No	ns	0,07902591	A-D
Column B vs. Column C	-0,8978	-1,074 to -0,7222	Yes	****	0,00000002	B-C
Column B vs. Column D	-0,8611	-1,037 to -0,6854	Yes	****	0,00000003	B-D
Column C vs. Column D	0,03674	-0,1389 to 0,2124	No	ns	0,92336026	C-D

Test details	Mean 1	Mean 2	Mean Diff,	SE of diff,	n1	n2	q	DF
Column A vs. Column B	0,8035	0,1022	0,7013	0,05917	4	4	16,76	12
Column A vs. Column C	0,8035	1	-0,1965	0,05917	4	4	4,698	12
Column A vs. Column D	0,8035	0,9633	-0,1598	0,05917	4	4	3,82	12
Column B vs. Column C	0,1022	1	-0,8978	0,05917	4	4	21,46	12
Column B vs. Column D	0,1022	0,9633	-0,8611	0,05917	4	4	20,58	12
Column C vs. Column D	1	0,9633	0,03674	0,05917	4	4	0,8783	12

A imply B

Number of families	1
Number of comparisons per family	6
Alpha	0,05

Tukey's multiple comparisons test	Mean Diff,	95,00% CI of diff,	Significant?	Summary	Adjusted P Value	
Column A vs. Column B	-0,1242	-0,3196 to 0,07127	No	ns	0,28389712	A-B
Column A vs. Column C	0,6871	0,4916 to 0,8825	Yes	****	0,00000118	A-C
Column A vs. Column D	0,02615	-0,1693 to 0,2216	No	ns	0,9777806	A-D
Column B vs. Column C	0,8112	0,6158 to 1,007	Yes	****	0,00000019	B-C
Column B vs. Column D	0,1503	-0,04511 to 0,3457	No	ns	0,15655595	B-D
Column C vs. Column D	-0,6609	-0,8563 to -0,4655	Yes	****	0,0000018	C-D

Test details	Mean 1	Mean 2	Mean Diff,	SE of diff,	n1	n2	q	DF
Column A vs. Column B	0,8758	1	-0,1242	0,06583	4	4	2,668	12
Column A vs. Column C	0,8758	0,1888	0,6871	0,06583	4	4	14,76	12
Column A vs. Column D	0,8758	0,8497	0,02615	0,06583	4	4	0,5619	12
Column B vs. Column C	1	0,1888	0,8112	0,06583	4	4	17,43	12
Column B vs. Column D	1	0,8497	0,1503	0,06583	4	4	3,229	12
Column C vs. Column D	0,1888	0,8497	-0,6609	0,06583	4	4	14,2	12

NOT A

Number of families	1
Number of comparisons per family	6
Alpha	0,05

Tukey's multiple comparisons test	Mean Diff,	95,00% CI of diff,	Significant?	Summary	Adjusted P Value	
Column A vs. Column B	-0,07943	-0,3129 to 0,1540	No	ns	0,74669468	A-B
Column A vs. Column C	0,5595	0,3261 to 0,7930	Yes	****	0,0000626	A-C
Column A vs. Column D	0,5784	0,3450 to 0,8118	Yes	****	0,00004517	A-D
Column B vs. Column C	0,639	0,4055 to 0,8724	Yes	****	0,0000166	B-C
Column B vs. Column D	0,6578	0,4244 to 0,8913	Yes	****	0,00001231	B-D
Column C vs. Column D	0,01888	-0,2145 to 0,2523	No	ns	0,99486299	C-D

Test details	Mean 1	Mean 2	Mean Diff,	SE of diff,	n1	n2	q	DF
Column A vs. Column B	0,9206	1	-0,07943	0,07862	4	4	1,429	12
Column A vs. Column C	0,9206	0,361	0,5595	0,07862	4	4	10,06	12
Column A vs. Column D	0,9206	0,3422	0,5784	0,07862	4	4	10,4	12
Column B vs. Column C	1	0,361	0,639	0,07862	4	4	11,49	12
Column B vs. Column D	1	0,3422	0,6578	0,07862	4	4	11,83	12
Column C vs. Column D	0,361	0,3422	0,01888	0,07862	4	4	0,3396	12

B nimply A

Number of families	1
Number of comparisons per family	6
Alpha	0,05

Tukey's multiple comparisons test	Mean Diff,	95,00% CI of diff,	Significant?	Summary	Adjusted P Value	
Column A vs. Column B	-0,8424	-0,9799 to -0,7049	Yes	****	<0,00000001	A-B
Column A vs. Column C	-0,2055	-0,3430 to -0,06801	Yes	**	0,00389294	A-C
Column A vs. Column D	-0,03647	-0,1740 to 0,1010	No	ns	0,85878805	A-D
Column B vs. Column C	0,6369	0,4994 to 0,7744	Yes	****	0,00000005	B-C
Column B vs. Column D	0,806	0,6685 to 0,9435	Yes	****	<0,00000001	B-D
Column C vs. Column D	0,169	0,03155 to 0,3065	Yes	*	0,01521705	C-D

Test details	Mean 1	Mean 2	Mean Diff,	SE of diff,	n1	n2	q	DF
Column A vs. Column B	0,1576	1	-0,8424	0,04631	4	4	25,72	12
Column A vs. Column C	0,1576	0,3631	-0,2055	0,04631	4	4	6,275	12
Column A vs. Column D	0,1576	0,194	-0,03647	0,04631	4	4	1,113	12
Column B vs. Column C	1	0,3631	0,6369	0,04631	4	4	19,45	12
Column B vs. Column D	1	0,194	0,806	0,04631	4	4	24,61	12
Column C vs. Column D	0,3631	0,194	0,169	0,04631	4	4	5,162	12

NOT B

Number of	1
Number of	6
Alpha	0,05

Tukey's m	Mean Diff,	95,00% CI	Significant	Summary	Adjusted P Value	
Column A	0,7655	0,6623 to	Yes	****	<0,000000	A-B
Column A	-0,0521	-0,1553 to	No	ns	0,467775	A-C
Column A	0,7588	0,6556 to	Yes	****	<0,000000	A-D
Column B	-0,8176	-0,9207 to	Yes	****	<0,000000	B-C
Column B	-0,00668	-0,1099 to	No	ns	0,997342	B-D
Column C	0,8109	0,7077 to	Yes	****	<0,000000	C-D

Test detail	Mean 1	Mean 2	Mean Diff,	SE of diff,	n1	n2	q	DF
Column A	0,9479	0,1824	0,7655	0,03476	4	4	31,14	12
Column A	0,9479	1	-0,0521	0,03476	4	4	2,12	12
Column A	0,9479	0,1891	0,7588	0,03476	4	4	30,87	12
Column B	0,1824	1	-0,8176	0,03476	4	4	33,26	12
Column B	0,1824	0,1891	-0,00668	0,03476	4	4	0,2718	12
Column C	1	0,1891	0,8109	0,03476	4	4	32,99	12

OR

Number of	1
Number of	6
Alpha	0,05

Tukey's m	Mean Diff,	95,00% CI	Significant	Summary	Adjusted P Value	
Column A	-0,5778	-0,7702 to	Yes	****	6,34E-06	A-B
Column A	-0,8652	-1,058 to	Yes	****	8E-08	A-C
Column A	-0,7892	-0,9815 to	Yes	****	2,2E-07	A-D
Column B	-0,2874	-0,4797 to	Yes	**	0,003904	B-C
Column B	-0,2113	-0,4037 to	Yes	*	0,030022	B-D
Column C	0,07603	-0,1163 to	No	ns	0,653757	C-D

Test detail	Mean 1	Mean 2	Mean Diff,	SE of diff,	n1	n2	q	DF
Column A	0,1348	0,7126	-0,5778	0,06479	4	4	12,61	12
Column A	0,1348	1	-0,8652	0,06479	4	4	18,89	12
Column A	0,1348	0,924	-0,7892	0,06479	4	4	17,23	12
Column B	0,7126	1	-0,2874	0,06479	4	4	6,273	12
Column B	0,7126	0,924	-0,2113	0,06479	4	4	4,613	12
Column C	1	0,924	0,07603	0,06479	4	4	1,66	12

NOR

Number of	1
Number of	6
Alpha	0,05

Tukey's m	Mean Diff,	95,00% CI	Significant	Summary	Adjusted P Value	
Column A	0,7903	0,6518 to	Yes	****	<0,000000	A-B
Column A	0,9824	0,8439 to	Yes	****	<0,000000	A-C
Column A	0,9845	0,8459 to	Yes	****	<0,000000	A-D
Column B	0,1921	0,05352 to	Yes	**	0,006758	B-C
Column B	0,1941	0,05558 to	Yes	**	0,006263	B-D
Column C	0,002056	-0,1365 to	No	ns	0,999967	C-D

Test detail	Mean 1	Mean 2	Mean Diff,	SE of diff,	n1	n2	q	DF
Column A	1	0,2097	0,7903	0,04666	4	4	23,95	12
Column A	1	0,01759	0,9824	0,04666	4	4	29,77	12
Column A	1	0,01553	0,9845	0,04666	4	4	29,84	12
Column B	0,2097	0,01759	0,1921	0,04666	4	4	5,821	12
Column B	0,2097	0,01553	0,1941	0,04666	4	4	5,883	12
Column C	0,01759	0,01553	0,002056	0,04666	4	4	0,06232	12

NAND

Number of	1
Number of	6
Alpha	0,05

Tukey's m	Mean Diff,	95,00% CI	Significant	Summary	Adjusted P Value	
Column A	0,2129	-0,05444 to	No	ns	0,137682	A-B
Column A	0,2342	-0,03310 to	No	ns	0,093396	A-C
Column A	0,7352	0,4678 to	Yes	****	1,58E-05	A-D
Column B	0,02135	-0,2460 to	No	ns	0,995055	B-C
Column B	0,5223	0,2549 to	Yes	***	0,000426	B-D
Column C	0,5009	0,2336 to	Yes	***	0,000615	C-D

Test detail	Mean 1	Mean 2	Mean Diff,	SE of diff,	n1	n2	q	DF
Column A	1	0,7871	0,2129	0,09005	4	4	3,344	12
Column A	1	0,7658	0,2342	0,09005	4	4	3,679	12
Column A	1	0,2648	0,7352	0,09005	4	4	11,55	12
Column B	0,7871	0,7658	0,02135	0,09005	4	4	0,3352	12
Column B	0,7871	0,2648	0,5223	0,09005	4	4	8,202	12
Column C	0,7658	0,2648	0,5009	0,09005	4	4	7,867	12

XOR

Number of	1
Number of	6
Alpha	0,05

Tukey's m	Mean Diff,	95,00% CI	Significant	Summary	Adjusted P Value	
Column A	-0,6704	-1,008 to	Yes	***	0,000366	A-B
Column A	-0,6818	-1,019 to	Yes	***	0,000314	A-C
Column A	-0,2562	-0,5936 to	No	ns	0,163969	A-D
Column B	-0,01135	-0,3488 to	No	ns	0,999622	B-C
Column B	0,4142	0,07676 to	Yes	*	0,015372	B-D
Column C	0,4256	0,08811 to	Yes	*	0,012907	C-D

Test detail	Mean 1	Mean 2	Mean Diff,	SE of diff,	n1	n2	q	DF
Column A	0,3182	0,9887	-0,6704	0,1137	4	4	8,342	12
Column A	0,3182	1	-0,6818	0,1137	4	4	8,483	12
Column A	0,3182	0,5744	-0,2562	0,1137	4	4	3,188	12
Column B	0,9887	1	-0,01135	0,1137	4	4	0,1412	12
Column B	0,9887	0,5744	0,4142	0,1137	4	4	5,154	12
Column C	1	0,5744	0,4256	0,1137	4	4	5,295	12

XNOR

Number of	1
Number of	6
Alpha	0,05

Tukey's m	Mean Diff,	95,00% CI	Significant	Summary	Adjusted P Value	
Column A	0,5367	0,3343 to	Yes	****	2,29E-05	A-B
Column A	0,5478	0,3454 to	Yes	****	1,86E-05	A-C
Column A	-0,2521	-0,4545 to	Yes	*	0,013994	A-D
Column B	0,01108	-0,1913 to	No	ns	0,998382	B-C
Column B	-0,7888	-0,9912 to	Yes	****	3,8E-07	B-D
Column C	-0,7999	-1,002 to	Yes	****	3,3E-07	C-D

Test detail	Mean 1	Mean 2	Mean Diff,	SE of diff,	n1	n2	q	DF
Column A	0,6756	0,1389	0,5367	0,06817	4	4	11,13	12
Column A	0,6756	0,1278	0,5478	0,06817	4	4	11,36	12
Column A	0,6756	0,9277	-0,2521	0,06817	4	4	5,23	12
Column B	0,1389	0,1278	0,01108	0,06817	4	4	0,2299	12
Column B	0,1389	0,9277	-0,7888	0,06817	4	4	16,36	12
Column C	0,1278	0,9277	-0,7999	0,06817	4	4	16,59	12

ANOVA summary	confidence interval	F	P value	P value summary	Significant diff. among means (P < 0.05)?	R squared	Dunnett's multiple comparisons test	Mean Diff,	95,00% CI of diff,	Significant?	Summary	Adjusted P Value
**Fig 4A**												
Ordinary one-way ANOVA	95	1858	< 1,0E-15	****	Yes	0,998	ß-gal_A239 [10ng] vs. N7 10ng	614479	589143 to 639814	Yes	****	<0,0001
							ß-gal_A239 [10ng] vs. N7 30ng	633544	608209 to 658879	Yes	****	<0,0001
							ß-gal_A239 [10ng] vs. N7 50ng	639803	614468 to 665138	Yes	****	<0,0001
							ß-gal_A239 [10ng] vs. N7 90ng	642712	617376 to 668047	Yes	****	<0,0001
**Fig 4B**												
Ordinary one-way ANOVA	95	99,37	<0,0001	****	Yes	0,9683	TEVp_G27_P7 (150ng) vs. N8 5ng	3,332	1,570 to 5,094	Yes	***	0,0006
							TEVp_G27_P7 (150ng) vs. N8 10ng	6,827	5,179 to 8,475	Yes	****	<0,0001
							TEVp_G27_P7 (150ng) vs. N8 20ng	9,279	7,631 to 10,93	Yes	****	<0,0001
							TEVp_G27_P7 (150ng) vs. N8 50ng	10,23	8,583 to 11,88	Yes	****	<0,0001
**Fig 4C**												
Ordinary one-way ANOVA	95	83,3	<0,0001	****	Yes	0,95968	TEVp_G27_P7_N8 (150ng) vs. N7 5ng	-0,0815	-2,182 to 2,019	**No**	**ns**	0,9999
							TEVp_G27_P7_N8 (150ng) vs. N7 10ng	-3,578	-5,523 to -1,633	Yes	***	0,0006
							TEVp_G27_P7_N8 (150ng) vs. N7 20ng	-6,56	-8,504 to -4,615	Yes	****	<0,0001
							TEVp_G27_P7_N8 (150ng) vs. N7 50ng	-11,06	-13,00 to -9,111	Yes	****	<0,0001
**Fig 4D**												
Ordinary one-way ANOVA	95	26,24	1,27785E-06	****	Yes	0,875	Lck(Y505F)_N266_P7 (10ng) vs. N8 5ng	4,049	1,080 to 7,019	Yes	**	0,0071
							Lck(Y505F)_N266_P7 (10ng) vs. N8 10ng	6,918	3,948 to 9,887	Yes	****	<0,0001
							Lck(Y505F)_N266_P7 (10ng) vs. N8 30ng	8,85	5,881 to 11,82	Yes	****	<0,0001
							Lck(Y505F)_N266_P7 (10ng) vs. N8 50ng	9,671	6,701 to 12,64	Yes	****	<0,0001
**Fig 4E**												
Ordinary one-way ANOVA	95	331,2	2E-14	****	Yes	0,9888	mIRAK1_F212 (10ng) vs. N7 10ng	1,975	1,786 to 2,164	Yes	****	<0,0001
							mIRAK1_F212 (10ng) vs. N7 30ng	1,963	1,774 to 2,152	Yes	****	<0,0001
							mIRAK1_F212 (10ng) vs. N7 50ng	1,993	1,803 to 2,182	Yes	****	<0,0001
							mIRAK1_F212 (10ng) vs. N7 90ng	2,041	1,852 to 2,230	Yes	****	<0,0001
**Fig 4F**												
Ordinary one-way ANOVA	95	208	6,09E-13	****	Yes	0,9823	mMyD88_N170 (10ng) vs. N7 10ng	12,03	10,43 to 13,62	Yes	****	<0,0001
							mMyD88_N170 (10ng) vs. N7 30ng	13,12	11,52 to 14,71	Yes	****	<0,0001
							mMyD88_N170 (10ng) vs. N7 50ng	13,63	12,04 to 15,23	Yes	****	<0,0001
							mMyD88_N170 (10ng) vs. N7 90ng	14,11	12,51 to 15,70	Yes	****	<0,0001
**Fig 4G**												
Ordinary one-way ANOVA	95	1095	< 1,0E-15	****	Yes	0,9966	dCas9_R535_N8 vs. N7 10ng	8,313	7,800 to 8,825	Yes	****	<0,0001
							dCas9_R535_N8 vs. N7 30ng	9,716	9,203 to 10,23	Yes	****	<0,0001
							dCas9_R535_N8 vs. N7 50ng	10,14	9,629 to 10,65	Yes	****	<0,0001
							dCas9_R535_N8 vs. N7 90ng	10,48	9,965 to 10,99	Yes	****	<0,0001
**Fig 4H**												
Ordinary one-way ANOVA	95	107,6	7,4542E-11	****	Yes	0,9663	N8_dCas9_R535_P7_VPR (1ng) vs. N7_NLS 1ng	-0,71	-0,9803 to -0,4397	Yes	****	<0,0001
							N8_dCas9_R535_P7_VPR (1ng) vs. N7_NLS 2,5ng	-1,222	-1,493 to -0,9520	Yes	****	<0,0001
							N8_dCas9_R535_P7_VPR (1ng) vs. N7_NLS 5ng	-1,593	-1,864 to -1,323	Yes	****	<0,0001
							N8_dCas9_R535_P7_VPR (1ng) vs. N7_NLS 10ng	-1,805	-2,076 to -1,535	Yes	****	<0,0001
**Fig 4I**												
Ordinary one-way ANOVA	95	188,4	1,26E-12	****	Yes	0,9805	TALA_T496_N6_N8 (30ng), N7:VP16 (30ng) vs. N5_NLS 10ng	7,748	5,766 to 9,730	Yes	****	<0,0001
							TALA_T496_N6_N8 (30ng), N7:VP16 (30ng) vs. N5_NLS 50ng	12,96	10,97 to 14,94	Yes	****	<0,0001
							TALA_T496_N6_N8 (30ng), N7:VP16 (30ng) vs. N5_NLS 90ng	15,05	13,07 to 17,03	Yes	****	<0,0001
							TALA_T496_N6_N8 (30ng), N7:VP16 (30ng) vs. N5_NLS 155ng	17,89	15,91 to 19,87	Yes	****	<0,0001
**Fig 4J**												
Ordinary one-way ANOVA	95	74	1,0849E-09	****	Yes	0,9518	N5_TALA_N6_N8 (1ng), N7:VP16 (10ng) vs. N6_NLS 1ng	-0,5653	-0,8034 to -0,3271	Yes	****	<0,0001
							N5_TALA_N6_N8 (1ng), N7:VP16 (10ng) vs. N6_NLS 3ng	-1,06	-1,298 to -0,8221	Yes	****	<0,0001
							N5_TALA_N6_N8 (1ng), N7:VP16 (10ng) vs. N6_NLS 5ng	-1,145	-1,383 to -0,9064	Yes	****	<0,0001
							N5_TALA_N6_N8 (1ng), N7:VP16 (10ng) vs. N6_NLS 10ng	-1,297	-1,535 to -1,059	Yes	****	<0,0001
**Fig 4K**												
Ordinary one-way ANOVA	95	33,97	2,55147E-09	****	Yes	0,9379	N5:scFV(S196_P7):41BB:cd3z vs. NeonGreen_N5 P8A 1,2µg	194	119,5 to 268,4	Yes	****	<0,0001
							N5:scFV(S196_P7):41BB:cd3z vs. NeonGreen_N5 P8A 3µg	267	192,6 to 341,4	Yes	****	<0,0001
							N5:scFV(S196_P7):41BB:cd3z vs. NeonGreen_N5 P8A 5,9µg	277,2	202,7 to 351,6	Yes	****	<0,0001
							N5:scFV(S196_P7):41BB:cd3z vs. NeonGreen_N5 P8A 12µg	285,1	210,6 to 359,5	Yes	****	<0,0001
							N5:scFV(S196_P7):41BB:cd3z vs. NeonGreen_N5 P8A 30µg	315,2	240,8 to 389,7	Yes	****	<0,0001
							N5:scFV(S196_P7):41BB:cd3z vs. NeonGreen_N5 P8A 60µg	331,8	257,3 to 406,2	Yes	****	<0,0001
							N5:scFV(S196_P7):41BB:cd3z vs. NeonGreen_N5 P8A 88µg	306	231,6 to 380,4	Yes	****	<0,0001
							N5:scFV(S196_P7):41BB:cd3z vs. NeonGreen_N5 P8A 120µg	325,6	251,2 to 400,1	Yes	****	<0,0001

ANOVA summary	confidence interval	F	P value	P value summary	Significant diff. among means (P < 0.05)?	R squared	Dunnett's multiple comparisons test	Mean Diff,	95,00% CI of diff,	Significant?	Summary	Adjusted P Value
**5A**												
Ordinary one-way ANOVA	95	97,8	1,2885E-10	****	Yes	0,9767	scFV(S196_P7):41BB:cd3z vs. NeonGreen_N5 P8A 5,5µg	74,63	26,62 to 122,6	Yes	**	<0,0001
							scFV(S196_P7):41BB:cd3z vs. NeonGreen_N5 P8A 11µg	83,3	35,28 to 131,3	Yes	***	<0,0001
							scFV(S196_P7):41BB:cd3z vs. NeonGreen_N5 P8A 22µg	101,4	53,42 to 149,4	Yes	***	<0,0001
							scFV(S196_P7):41BB:cd3z vs. NeonGreen_N5 P8A 44µg	134,5	86,48 to 182,5	Yes	****	<0,0001
							scFV(S196_P7):41BB:cd3z vs. NeonGreen_N5 P8A 88µg	221,2	173,2 to 269,2	Yes	****	<0,0001
**5B**												
Ordinary one-way ANOVA	95	33,97	2,5515E-09	****	Yes	0,9379	N5:scFV(S196_P7):41BB:cd3z vs. NeonGreen_N5 P8A 1,2µg	194	119,5 to 268,4	Yes	****	<0,0001
							N5:scFV(S196_P7):41BB:cd3z vs. NeonGreen_N5 P8A 3µg	267	192,6 to 341,4	Yes	****	<0,0001
							N5:scFV(S196_P7):41BB:cd3z vs. NeonGreen_N5 P8A 5,9µg	277,2	202,7 to 351,6	Yes	****	<0,0001
							N5:scFV(S196_P7):41BB:cd3z vs. NeonGreen_N5 P8A 12µg	285,1	210,6 to 359,5	Yes	****	<0,0001
							N5:scFV(S196_P7):41BB:cd3z vs. NeonGreen_N5 P8A 30µg	315,2	240,8 to 389,7	Yes	****	<0,0001
							N5:scFV(S196_P7):41BB:cd3z vs. NeonGreen_N5 P8A 60µg	331,8	257,3 to 406,2	Yes	****	<0,0001
							N5:scFV(S196_P7):41BB:cd3z vs. NeonGreen_N5 P8A 88µg	306	231,6 to 380,4	Yes	****	<0,0001
							N5:scFV(S196_P7):41BB:cd3z vs. NeonGreen_N5 P8A 120µg	325,6	251,2 to 400,1	Yes	****	<0,0001
**5C**												
Ordinary one-way ANOVA	95	114,4	4,4079E-11	****	Yes	0,98	N5:scFV Her2 (199_P7):41BB:cd3z vs. NeonGreen_N5 P8A 5,5µg	30,67	-1,939 to 63,27	**No**	ns	<0,0001
							N5:scFV Her2 (199_P7):41BB:cd3z vs. NeonGreen_N5 P8A 11µg	110,5	77,89 to 143,1	Yes	****	<0,0001
							N5:scFV Her2 (199_P7):41BB:cd3z vs. NeonGreen_N5 P8A 22µg	147,5	114,9 to 180,1	Yes	****	<0,0001
							N5:scFV Her2 (199_P7):41BB:cd3z vs. NeonGreen_N5 P8A 44µg	176,3	143,7 to 208,9	Yes	****	<0,0001
							N5:scFV Her2 (199_P7):41BB:cd3z vs. NeonGreen_N5 P8A 88µg	190,8	158,2 to 223,4	Yes	****	<0,0001

Supplementary Table S3

	Name	insert_name	works	%WT_activity	**max_fold_decrease _with_peptide**	ligand
Lck	N266	P7	Y	N/A	3,80	N8
	G278	P7	Y	N/A	1,70	N8
	T375	P7	Y	N/A	1,90	N8
	Q256	P7	Y	N/A	1,90	N8
CAR_T	N184	P7	Y			N8
	N195	P7	Y	54,00	3,00	N8
	T51	P7	N	0,00	N/A	N8
	I193	P7	N/A	20,00	N/A	N8
	S199	P7	N/A	28,00	N/A	N8
TEVp	G27	P7	Y	82,00	5,10	N8
	L72	P7	N	4,00	1,50	N8
	I77	P7	Y	47,00	5,20	N8
	G79	P7	Y	92,00	6,40	N8
	K147	P7	N	1,00	1,10	N8
	F172	P7	N	2,00	1,30	N8
	T175	P7	N	1,00	2,10	N8
	K184	P7	N	1,00	1,70	N8
	G212	P7	N	3,00	1,40	N8
TALEA	T496	N6	Y	65,00	3,00	N5
dCas	S55	N8	N	0,10	1,09	N7
	D147	N8	N	4,50	7,10	N7
	R535	N8	Y	43,30	5,71	N7
	G1104	N8	N	0,10	1,43	N7
	K1153	N8	Y	53,20	2,18	N7
Bgal	L25	N8	N	7,40	0,72	N7
	A35	N8	N	0,10	1,03	N7
	T45	N8	Y	35,70	4,31	N7
	L55	N8	N	46,10	0,89	N7
	D233	N8	Y	123,10	7,39	N7
	A229	N8	N	3,70	2,21	N7
	A239	N8	Y	17,40	42,18	N7
	D507	N8	N	0,10	1,05	N7
	P513	N8	N	18,80	1,48	N7
	D610	N8	N	1,00	13,04	N7
	T799	N8	N	67,10	0,75	N7
	S1000	N8	N	1,60	12,08	N7
MyD88	T66	N8	N	8,90	3,62	N7
	G80	N8	Y	43,30	9,16	N7
	N170	N8	Y	367,20	12,17	N7
	S209	N8	Y	406,00	12,76	N7
	S224	N8	Y	378,10	12,48	N7
mIRAK1	F212	N8	Y	171,80	24,53	N7
	G221	N8	Y	222,80	34,28	N7
	R232	N8	Y	137,90	22,18	N7
	S281	N8	N	17,90	2,64	N7
ngGFP	C143	P7	Y			N8
	K214	P7	N			N8
fLuc	K493	P7	Y			N8
	E428	P7	N	0,00		N8
	T202	P7	N	0,00		N8
	K493	N8	Y	0,00	91,60	N7
	K493	5gs:N8	Y	0,00	34,50	N7
	K493	10gs:N8	Y	0,00	3,90	N7
