# Supplementary material for: Designed allosteric protein logic
Source: Cell Discov. 2024 Jan 16;10:8. doi: 10.1038/s41421-023-00635-y (PMC10791696; doi:10.1038/s41421-023-00635-y)
Supplement: Supplementary file 3 — Supplementary table and file legend [file 41421_2023_635_MOESM3_ESM.docx]

Supplementary table titles

Supplementary Table S1. Amino acid sequences of constructs were used in this study. Related to methods.

Supplementary Table S2. Detailed statistical analysis. Related to Figs. [1](#MEP_L_fig1)–[5](#MEP_L_fig5).

Supplementary Table S3. List of all tested INSRTR variants denoting activity and high success rate.

Supplementary File S1. Descriptions of features calculated for the inserter model. Values for all the descriptions for the 47 constructs used to train the model.
